# Exploring Alkyl Ester Salts of L-Amino Acid Derivatives of Ibuprofen: Physicochemical Characterization and Transdermal Potential

**DOI:** 10.3390/molecules28227523

**Published:** 2023-11-10

**Authors:** Kordian Witkowski, Anna Nowak, Wiktoria Duchnik, Łukasz Kucharski, Łukasz Struk, Paula Ossowicz-Rupniewska

**Affiliations:** 1 Department of Chemical Organic Technology and Polymeric Materials, Faculty of Chemical Technology and Engineering, West Pomeranian University of Technology in Szczecin, Piastów Ave. 42, 71-065 Szczecin, Poland; 2 Department of Cosmetic and Pharmaceutical Chemistry, Pomeranian Medical University in Szczecin, Powstańców Wielkopolskich Ave. 72, 70-111 Szczecin, Poland; 3 Department of Organic and Physical Chemistry, Faculty of Chemical Technology and Engineering, West Pomeranian University of Technology in Szczecin, Al. Piastów 42, 71-065 Szczecin, Poland; lukasz.struk@zut.edu.pl

**Keywords:** ibuprofen, transdermal drug delivery system, chemical modification of an active pharmaceutical agent, nonsteroidal anti-inflammatory drug, permeability, bioavailability

## Abstract

This research presents novel ibuprofen derivatives in the form of alkyl ester salts of L-amino acids with potential analgesic, anti-inflammatory, and antipyretic properties for potential use in transdermal therapeutic systems. New derivatives of (*RS*)-2-[4-(2-methylpropyl)phenyl]propionic acid were synthesized using hydrochlorides of alkyl esters (ethyl, propyl, isopropyl, butyl, *sec*-butyl, *tert*-butyl, and pentyl) of L-glutamine. These were further transformed into alkyl esters of L-amino acid ibuprofenates through neutralization and protonation reactions. Characterization involved spectroscopic methods, including nuclear magnetic resonance and Fourier-transform infrared spectroscopy. Various physicochemical properties were investigated, such as UV–Vis spectroscopy, polarimetric analysis, thermogravimetric analysis, differential scanning calorimetry, X-ray diffraction, water solubility, octanol/water partition coefficient, and permeability through pig skin using Franz diffusion cells. The research confirmed the ionic structure of the obtained hydrochlorides of alkyl esters of L-amino acids and ibuprofenates of alkyl esters of L-glutamic acid. It revealed significant correlations between ester chain length and thermal stability, crystallinity, phase transition temperatures, lipophilicity, water solubility, skin permeability, and skin accumulation of these compounds. Compared to the parent ibuprofen, the synthesized derivatives exhibited higher water solubility, lower lipophilicity, and enhanced skin permeability. This study introduces promising ibuprofen derivatives with improved physicochemical properties, highlighting their potential for transdermal therapeutic applications. The findings shed light on the structure–activity relationships of these derivatives, offering insights into their enhanced solubility and skin permeation, which could lead to more effective topical treatments for pain and inflammation.

## 1. Introduction

In a study by Janus et al., it was observed, through in vitro permeability assessments using pig skin, that the ibuprofen racemate, when administered at a concentration of 0.01 g·cm^−3^, exhibited a skin penetration rate of merely 6% [1]. It is well-established that alterations in the compound’s structure can effectively modulate the solubility and permeability characteristics of ibuprofen.

The most straightforward modification involves the conversion of a carboxylic acid into its corresponding salt using a base, a process exemplified by the synthesis of ibuprofen sodium salt. Legg et al. conducted a study to investigate variations in absorption kinetics among different forms of ibuprofen, including ibuprofen sodium (IBUNa) and ibuprofen racemate (rac-IBU). Their research, involving 71 healthy adult participants, revealed that when administered on an empty stomach, IBUNa exhibited an approximately 30% higher maximum concentration in the body (C_max_) compared to rac-IBU. Furthermore, the time to reach C_max_ (T_max_) was notably earlier for IBUNa, with a difference of approximately 1.0–1.5 h [2]. Additionally, there exist other known ibuprofen salts, such as potassium, calcium, magnesium, aluminum, zinc, and copper [3,4,5,6,7].

The exploration of ion pair formation has been undertaken to enhance membrane permeability, thereby increasing the bioavailability of hydrophilic ionized molecules. According to this concept, when oppositely charged molecules interact, they form an ion pair, effectively neutralizing their overall electric charge. This transformation results in heightened lipophilicity of the ion pair compared to the individual ions, facilitating greater molecule penetration through various membranes, such as those found in the intestines, skin, or synthetic systems [8,9,10].

Sarveiya et al. investigated the impact of alkylamine counterions, including ethylamine, diethylamine, triethylamine, and ethylenediamine, on the permeation of ibuprofen through a polydimethylsiloxane membrane, employing propylene glycol as a solvent. The control sample, sodium ibuprofenate, exhibited log P values of approximately 0.92 and a 3.09 μg·cm^−2^ permeability. Their study revealed that the introduction of a counterion significantly influenced the partition coefficient and permeability of the drug through the membrane. Triethylammonium ibuprofenate demonstrated a permeation value of 48.14 μg·cm^−2^ and a log P of 1.18, demonstrating the potential to enhance salt flow through lipophilic membranes using the ion pair approach. The degree of enhancement depends on factors such as lipophilicity, the extent of ion pairing, and the reduction in charge in the drug molecule [8].

Another example of research aimed at increasing the solubility parameters of active pharmaceutical ingredients (API) in water comes from H. S. Alghurabi, who examined the influence of amino acid counterions, specifically lysine and arginine, on ibuprofen’s solubility. This work resulted in amino acid salts of ibuprofen with significantly increased solubility at room temperature. Notably, the introduction of this modification increased solubility by 1371 and 242 times, reaching 85.0 ± 8.9 mg·cm^−3^ and 14.0 ± 3.7 mg·cm^−3^ for arginine and lysine derivatives, respectively. The authors attribute this to the higher pKa value of arginine, owing to the guanidine moiety in its molecule, compared to lysine, which features an amino group at the end of its chain [11].

In our previous study, ibuprofenate salts of L-valine esters were synthesized and evaluated for changes in solubility, partition coefficient, and permeability through pig skin. Our team successfully synthesized ethyl, isopropyl, propyl, butyl, pentyl, and hexyl esters of L-valine. The research revealed that the synthesis of such salts positively impacted the bioavailability of ibuprofen, with these compounds displaying improved solubility in water and phosphate buffers at pH 5.4 and 7.4. Notably, the propyl and isopropyl esters exhibited the highest permeability (677.41 and 604.51 μg IBU·cm^−2^, respectively) compared to ibuprofen (302.84 μg IBU·cm^−2^). The study’s authors also noted the release of L-valine alongside ibuprofen, highlighting its potential positive effects on bodily functions, including reduced fatigue and participation in pantothenic acid (vitamin B5) synthesis, which supports wound treatment [1,12].

One of our latest studies described ibuprofenate esters of isopropyl L-amino acids, resulting in 14 such compounds through a three-step reaction. The solvent-free method was employed for synthesis, effectively reducing the presence of volatile organic compounds and minimizing process costs. Furthermore, this reaction virtually eliminates by-products, resulting in a highly pure end product [13]. The ibuprofen salts obtained significantly increased the solubility of the active compound in both water (25 °C) and PBS (pH = 7.4, 32 °C). Moreover, all derivatives showed 2.95 to 16.52 times greater permeability of the active substance compared to ibuprofen. The highest permeability was achieved with the ibuprofenate ester of isopropyl L-lysine, which also exhibited an increased transport rate and the highest accumulated mass of penetrated ibuprofen. It was also demonstrated that the amino acid derivatives of ibuprofen obtained, showed 3.3 to 6.7 times greater accumulation in the skin. These results suggest the potential application of the obtained compounds as alternatives to poorly soluble NSAIDs for skin applications [13].

Ibuprofenates of alkyl amino acid esters, a relatively niche area of research, have garnered attention for their potential pharmaceutical applications, particularly in improving drug delivery and enhancing the therapeutic effects of ibuprofen. Ibuprofenates of alkyl amino acid esters have been explored as potential prodrugs to enhance the transdermal delivery of ibuprofen. These compounds are designed to improve the drug’s solubility, permeability through the skin, and overall bioavailability. Such formulations aim to provide more efficient and controlled drug release. One of the primary advantages of these derivatives is their ability to increase the solubility of ibuprofen. This can be particularly useful for developing formulations with higher drug concentrations, which may be beneficial for specific therapeutic applications.

This publication is a continuation of research on amino acid alkyl ester ibuprofenates to increase the active substance’s bioavailability. In this research, we will obtain and characterize the obtained derivatives of alkyl esters of glutamic acid.

## 2. Results and Discussion

In our previous research, we explored the impact of varying amino acid alkyl ester chain lengths on the properties of synthesized L-valine alkyl ester ibuprofenates and examined the influence of different amino acids in amino acid isopropyl ester ibuprofenates. A diverse set of amino acids, including glycine, L-alanine, L-valine, L-isoleucine, L-leucine, L-serine, L-threonine, L-cysteine, L-methionine, L-aspartic acid, L-lysine, L-phenylalanine, and L-proline, were employed in these investigations. Our findings revealed that various structural changes in the molecule impact skin permeability and solubility in water and buffer solutions, biodegradability, and lipophilicity [1,13,14].

Our preceding studies demonstrated that not all amino acid alkyl ester hydrochlorides (precursors for ibuprofen derivatives) can be obtained using trimethylsilyl chloride as the chlorinating agent [13]. In this publication, we employed chlorotri(methyl)silane as the chlorinating agent and focused on two specific amino acids: glutamine and glutamic acid. Notably, we observed that when an acid amide, L-glutamine, was used as the amino acid substrate, it led to the formation of acidic L-amino acid alkyl ester hydrochlorides. This observation provides evidence that the amide moiety undergoes acidic hydrolysis in response to the chlorinating agent’s influence. The same phenomenon was observed using asparagine and aspartic acid. Furthermore, the hydroxyl group, replacing the amino group, underwent esterification by alcohol, resulting in the esterification of both carboxyl groups of the amino acid. A similar reaction pattern was observed when utilizing L-glutamic acid for synthesis, where both carboxyl groups of the amino acid underwent esterification.

When used to synthesize *tert*-butyl alcohol, obtaining the expected alkyl ester hydrochloride was impossible. Probably due to steric reasons and the geometry of the alcohol molecule, it was not possible to obtain an ester. As is known, tertiary alcohols are less reactive than primary or secondary alcohols, so the esterification process may be more difficult when using them. In order to obtain *tert*-butyl ester hydrochlorides, more aggressive reaction conditions or other synthesis methods would have to be used.

Following a three-step reaction sequence, L-glutamic acid alkyl ester ibuprofenates were successfully synthesized, with yields ranging from 73% to 91%. In the final step of this process, the corresponding L-glutamic acid alkyl ester was combined with ibuprofen in the presence of chloroform.

The identity of the obtained compounds, i.e., amino acid alkyl ester hydrochlorides and amino acid alkyl ester ibuprofenates, was confirmed on the basis of analyses of ^1^H, ^13^C NMR, and FTIR spectra. Individual spectra and the interpretation of the spectra, chemical shifts of the signals, their multiplicity, integration, coupling constants (for proton spectra), and chemical shifts of the signals (for carbon spectra) are presented in the Supplementary Materials (Figures S1–S34). Integration in the ^1^H NMR spectra was performed with respect to the proton located on the α carbon derived from the amino acid (proton designation 4), which occurs at a shift of approximately 4.5 ppm.

The formation of amino acid alkyl ester hydrochlorides was confirmed by detecting a signal in the ^1^H NMR spectrum, indicating ammonium group protonation and the desired ionic compound formation. It was noticed that the effect of revealing the signal coming from the ammonium group increased with the increase in the length of the alkyl chain. Additionally, the signal was more intense in compounds with branched chains (isopropyl, *sec*-butyl) than in their straight-chain counterparts.

Based on the analysis of ^1^H NMR spectra, it was confirmed that in the case of the syntheses of ibuprofenate alkyl esters of L-amino acids, one of the ester bonds was hydrolyzed, which led to obtaining a salt with one carboxyl group of the amino acid esterified. Additionally, in the ^1^H NMR spectra, signals from protons in the ammonium group were not visible.

The obtained compounds exhibited characteristic absorption bands in the range of 1230 cm^−1^, 1460 cm^−1,^ and 1740 cm^−1^, corresponding to C-O, C-N, and C=O stretching vibrations, characteristic of α-amino acids. Additionally, the bands at 2800–3000 cm^−1^ indicated overlapping C-H and N-H stretching vibrations. The appearance of a symmetrical vibration band at around 1380 cm^−1^ confirmed the ionic structure of ibuprofen salts. Although an asymmetric vibration band around 1600 cm^−1^ was suggested, it was partially obscured by C=O stretching vibrations. These vibrations, along with the characteristic functional groups, confirmed the identity and ionic structure of the obtained hydrochlorides and ibuprofenates of alkyl esters of L-amino acids.

In the UV–Vis spectroscopy analysis, the obtained L-amino acid alkyl ester hydrochlorides showed one characteristic absorption band, showing the maximum absorption at a wavelength in the range of 210.4–220.6 nm. However, in the case of L-glutamic acid alkyl ester ibuprofenates, two absorption bands are visible, with their highest values at 223.2–230.0 nm and 259.2–264.0 nm, respectively. It is worth mentioning that ibuprofen shows maximum absorbance at a wavelength of 260 nm.

**Figure 1:**
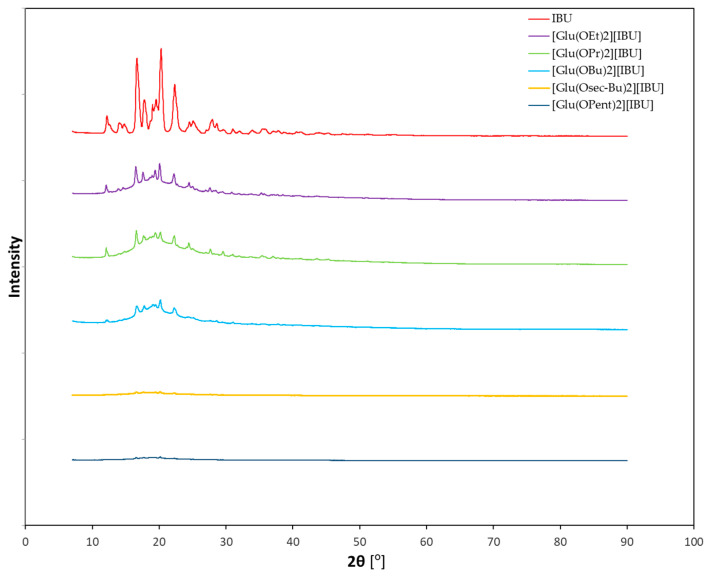
X-ray diffraction patterns of ibuprofen and ibuprofenates of L-glutamic acid alkyl esters.

Figure 1 shows the diffractograms of ibuprofenates of L-glutamic acid alkyl esters, while the individual diffractograms of hydrochlorides and ibuprofenates are presented in the Supplementary Materials (Figures S35–S44). It was noticed that the diffractograms of the obtained ibuprofen salts differ significantly from the diffractogram of the standard—ibuprofen. As the alkyl chain of the ester increases, the intensity of the reflections decreases, indicating that the crystalline character disappears and the derivatives with a longer chain are amorphous.

The results of the optical, specific, and molar rotation of the obtained L-glutamic acid alkyl ester hydrochlorides and ibuprofenates are summarized in Table 1. All obtained and L-glutamic acid alkyl ester hydrochlorides and ibuprofenates rotate the plane of polarized light to the right (+), with the hydrochlorides showing higher optical rotation values than their ibuprofenate counterparts. The highest value was demonstrated by [Glu(OiPr)_2_][HCl]. Amino acid alkyl ester hydrochlorides show a much higher rotation angle than the amino acids from which they are obtained. A racemic mixture of ibuprofen was used for the syntheses, which is why this substrate could not rotate polarized light. The obtained ibuprofenates have a specific rotation of light within +2° (except for the isopropyl derivative +9.8°).

TG, DTG, and C-DTA curves are provided in the Supplementary Materials (Figures S45–S57). Table 2 lists the temperatures that characterize the stability of the obtained amino acid alkyl ester hydrochlorides and ibuprofenates.

All compounds showed thermal stability up to over 160 °C. It was noticed that as the alkyl chain of the ester increases, the thermal stability of the compounds increases, which confirms the literature reports [1]. Additionally, compounds with branched alkyl chains, such as isopropyl and *sec*-butyl chains, further increased thermal stability compared to their n-propyl and n-butyl isomers. In the case of ibuprofenates, during heating, the fastest decomposition was [Glu(OEt)][IBU], which was characterized by the onset of decomposition at a temperature of 194.2 °C, while [Glu(OiPr)][IBU] decomposed only at 222.9 °C, which was the highest value among all ibuprofen salts tested. As in the case of hydrochlorides, the first derivative of mass loss over time was determined, thanks to which the temperatures of the fastest decomposition of the sample were determined. The highest temperature is 247.7 °C—[Glu(OBu)][IBU], and the lowest is 228.3 °C—[Glu(OEt)][IBU].

DSC analyses determined the temperatures of phase transformations occurring due to temperature changes in the sample. Individual thermograms are listed in the Supplementary Materials (Figures S58 and S59), and the values of phase transformation temperatures and their enthalpy energies are listed in Table 2. Hydrochlorides of alkyl esters of L-glutamic acid melted at temperatures lower than the above-mentioned derivatives of L-aspartic acid. Melting points were >100 °C. All hydrochlorides had glass transition temperatures from approximately −45 to −17 °C. No crystallization temperatures were determined for ibuprofenates of L-glutamic acid alkyl esters, while melting points were determined for ethyl, propyl, and butyl derivatives (50.43, 59.02, 71.05 °C, respectively). Glass transition temperatures of approximately −50 °C were determined for all ibuprofenates. Moreover, it has been noted that some L-glutamic acid alkyl ester ibuprofenates have significantly lower melting points than the corresponding L-glutamic acid alkyl ester hydrochlorides. For example, [Glu(OEt)_2_][HCl] melts at a temperature of about 101 °C, and [Glu(OEt)][IBU] melts at half that temperature—about 50 °C. Interestingly, in the case of [Glu(OBu)_2_][HCl], it is the opposite—the hydrochloride has a melting point of about 64 °C, and ibuprofenate [Glu(OBu)][IBU]—71 °C.

All L-glutamic acid alkyl ester ibuprofenates showed a positive log P value, although lower than ibuprofen, which confirms that the introduction of the L-amino acid alkyl ester cation reduces the log P value. The obtained salts can be classified as medium-lipophilic compounds. Ibuprofen has the highest lipophilic character (log P = 3.37), and [Glu(OEt)][IBU] has the lowest (log P = 2.37). The partition coefficient and water solubility parameters of the obtained L-glutamic acid alkyl ester ibuprofenates are listed in Table 3. The solubility of the compound in g·dm^−3^ and the solubility in terms of the active substance (ibuprofen) in g IBU·dm^−3^ are presented.

The lipophilicity of the obtained compounds can be arranged in ascending order: [Glu(OEt)][IBU] < [Glu(OPr][IBU] < [Glu(OPent)][IBU] < [Glu(OiPr)][IBU] < [Glu (OBu)][IBU] < [Glu(O*sec*-Bu)][IBU] < [IBU]. It can be seen that with the increase in the alkyl chain length, with the exception of [Glu(OPent)][IBU]), lipophilicity increases. The salt of L-glutamic acid alkyl ester with the longest alkyl chain showed a lipophilicity value lower than the n-butyl derivative. This is quite an unusual trend. Branching of the alkyl chain increases the lipophilicity of the compound—the same tendency in the case of derivatives with a chain length of C3 and C4.

The solubility of the compounds per active mass of ibuprofen was higher than the standard (ibuprofen). Ibuprofenate, which showed the highest solubility in water at 25 °C ([Glu(OiPr)][IBU]—0.203 ± 0.006 g IBU·dm^−3^), dissolved approximately three times better than ibuprofen. However, the compound that achieved the lowest solubility value ([Glu(O*sec*-Bu)][IBU]—0.093 ± 0.012 g IBU·dm^−3^) was characterized by a solubility 1.24 times higher than the standard. The solubility of compounds can be arranged in ascending order as follows: IBU < [Glu(O*sec*-Bu)][IBU] < [Glu(OEt)][IBU] < [Glu(OPent)][IBU] < Glu(OBu)][IBU] < [Glu(OPr)][IBU] < [Glu(OiPr)][IBU]. There is no typical relationship between the length of the alkyl chain, according to which the longer the chain, the lower the solubility of the compound in water.

**Figure 2:**
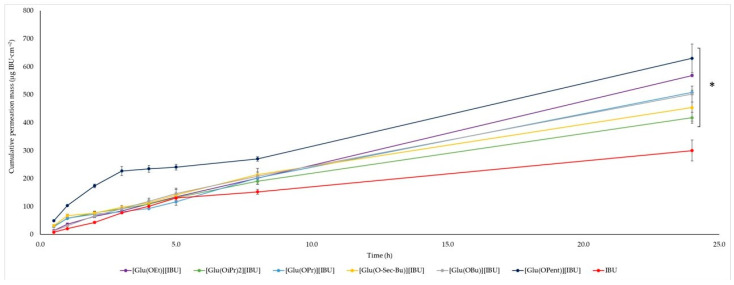
Ibuprofen and L-glutamic acid alkyl ester ibuprofenates’ permeation profiles. Values are the means with standard deviation; *n* = 3. For * *p* < 0.001 versus the control (pure ibuprofen).

**Figure 3:**
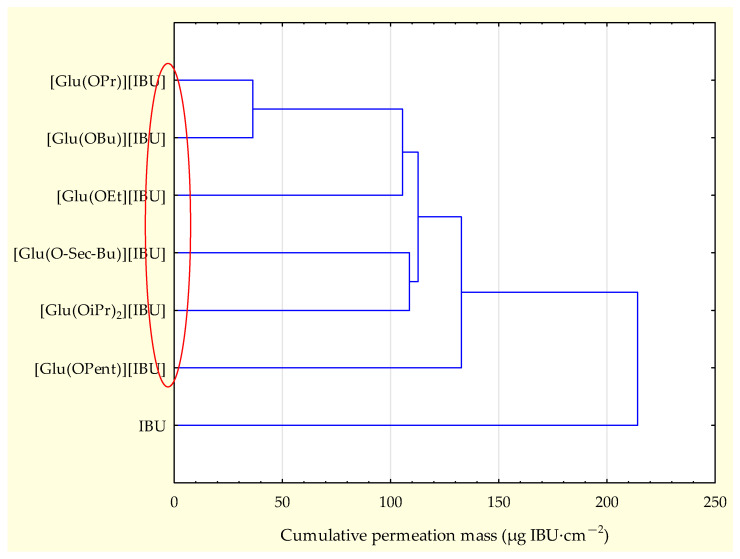
Cluster analysis graph for the cumulative mass of ibuprofen and its derivatives after 24 h study. In the cluster analysis graph, the compounds form one separate group characterized by similar statistical permeability (red circle).

**Figure 4:**
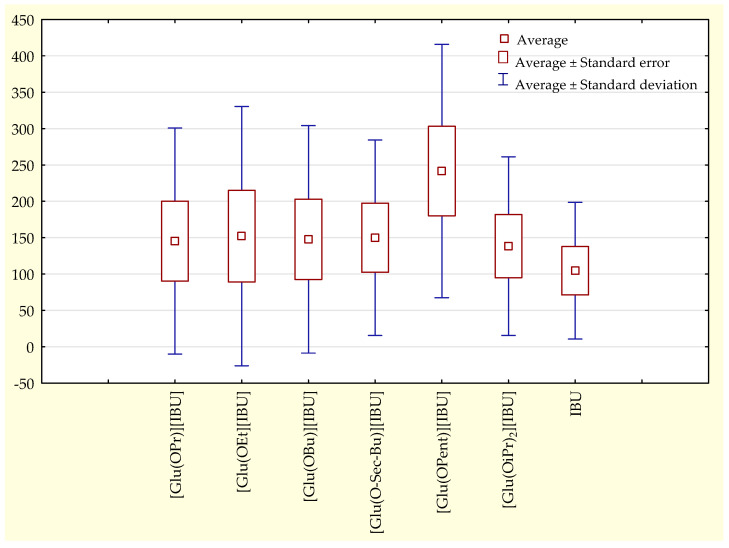
The box plot for the cumulative mass of ibuprofen and its derivatives throughout the 24-h study.

The permeability profiles for ibuprofen and its salts are shown in Figure 2 and Table 4—the cumulative mass and permeability parameters of ibuprofen and L-glutamic acid alkyl ester ibuprofenates after 24 h of testing. The compound [Glu(OPent)][IBU], showed the highest skin permeability value after 24 h of testing (630.443 ± 51.226 μg IBU∙cm^−2^), and the lowest was the parent ibuprofen (300.183 ± 37.443 μg IBU∙cm^−2^). Permeability values after 24 h of permeation testing for the obtained compounds can be ordered from the highest to the lowest, respectively: [Glu(OPent)][IBU] > [Glu(OEt)][IBU] > [Glu(OPr)][IBU] > [Glu (O*sec*-Bu)][IBU] > [Glu(OBu)][IBU] > [Glu(OiPr)][IBU] > IBU. The accumulated mass of all analyzed derivatives after 24 h of penetration was significantly higher compared to pure IBU. This is confirmed by the cluster analysis test, where all derivatives form a separate cluster (Figure 3). The compound [Glu(OPent)][IBU], permeated to the highest extent throughout the period of the study, as shown in the box plot (Figure 4).

The steady-state permeation values of [Glu(OPr)][IBU], [Glu(OiPr)][IBU], and [Glu(O*sec*-Bu)][IBU] were similar to those of ibuprofen, [Glu(OPent)][IBU] showed the highest J_ss_ and K_p_ values (57.098 and 5.267, respectively), the lowest values were shown by the isopropyl derivative (26.451 and 2.686). The delay time, diffusion coefficient, and skin partition coefficient were calculated only for IBU and [Glu(OBu)][IBU]. In other cases, cross-talk profiles (no delay time) made calculations impossible. In this case, a steady state was not obtained. Therefore, the steady-state slope could not be determined on the graph, and the intercepts in the trend line equations of these compounds showed positive values. The fact that the delay time parameter was not calculated indicates that most of the L-glutamic acid alkyl ester ibuprofenates obtained penetrate the skin immediately, without any delay.

**Figure 5:**
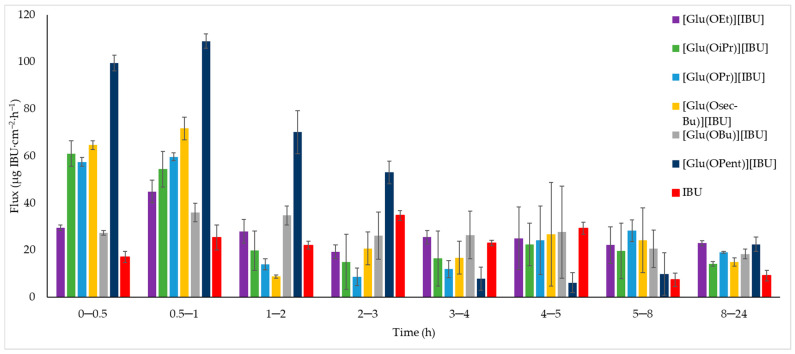
The permeation rate of ibuprofen and L-glutamic acid alkyl ester ibuprofenates during the 24 h permeation; α = 0.05 (mean ± SD, *n* = 3).

The permeation rates were also determined, which are visible in Figure 5. The graph shows the amount of ibuprofen mass per hour calculated between measurement points. It can be seen that the permeation rate increases for all compounds from the point 0.0–0.5 to 0.5–1 and then decreases. Within the first two hours, the highest value is reached by [Glu(OPent)][IBU], twice as high as ibuprofen, and the lowest among ibuprofenates is [Glu(OiPr)][IBU] (417.994 μg IBU∙cm^−2^), which penetrated almost 40% better than ibuprofen (300.183 μg IBU∙cm^−2^). The permeation rate of compounds from the point 0.0–1 to 1.0–2.0 drops dramatically, with [Glu(OPent)][IBU] maintaining the highest value among the rest. After 24 h, the highest value of the permeation rate was demonstrated by [Glu(OEt)][IBU] (difference of approximately 0.5 μg∙h^−1^ compared to [Glu(OPent)][IBU]. The rest of the compounds achieved similar values, and the lowest value shows [IBU].

**Figure 6:**
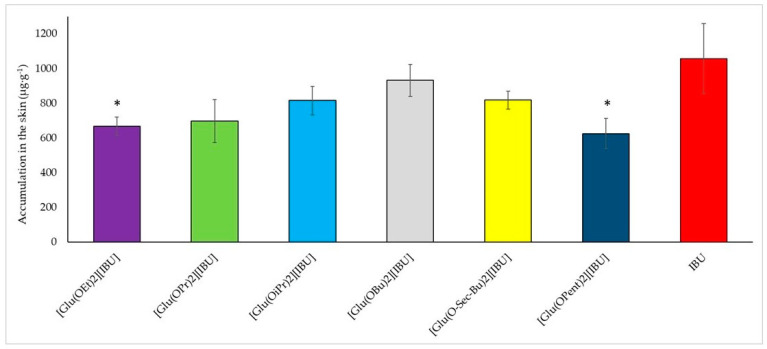
Accumulation in the skin of ibuprofen and L-glutamic acid alkyl ester ibuprofenates during the 24 h permeation; α = 0.05 (mean ± SD, *n* = 3). For * *p* < 0.001 versus the control (pure ibuprofen).

In addition to penetrating, the compounds can accumulate in the skin. Figure 6 shows a graph of the accumulation of the compound in pig skin depending on the type of compound used for testing. The lowest accumulation was recorded for the compound [Glu(OPent)][IBU] (625.895 ± 87.275 µg IBU·g^−1^ pig skin), and the highest for ibuprofen (1057.468 ± 200.589 µg IBU·g^−1^ pig skin). The highest value for the obtained derivative was recorded for the compound [Glu(OBu)][IBU] (931.823 ± 93.449 µg IBU·g^−1^ pig skin), which is equal to approximately 90% of the accumulated mass for ibuprofen. A visible relationship indicates that as the length of the alkyl chain increases, the accumulation in the skin increases and reaches a maximum at the butyl chain. It was also noticed that the accumulation in the skin decreases as the permeability increases. Compounds with the highest rate of penetration through pig skin, such as [Glu(OPent)][IBU] and [Glu(OEt)][IBU], achieved the lowest accumulation values in the skin. IBU, which had the lowest penetration rate among the tested compounds, was also characterized by the highest accumulation in the skin.

## 3. Materials and Methods

### 3.1. Materials

All reagents used were commercially available compounds that were used without prior purification. (*RS*)-ibuprofen (99%) was purchased from Acros Organics (Geel, Belgium), L-glutamic acid (>99%), and L-glutamine (>99%) were purchased from Carl Roth (Karlsruhe, Germany). Chlorotrimethylsilane (>99%) (TMSCl) was purchased from Sigma-Aldrich (Steinheim am Albuchem, Germany). Ethanol (EtOH), propan-1-ol (PrOH), butan-1-ol (BuOH), butan-2-ol (*sec*-BuOH), 2-methylpropan-2-ol (t-BuOH), pentan-1-ol (PentOH), chloroform, diethyl ether were of high purity, provided by Chempur (Gliwice, Poland). Ammonia solution 25% (NH_3_·H_2_O) of analytical grade was purchased from StanLab (Lublin, Poland). Deuterated chloroform (CDCl_3_) (99.8%) (+0.03 TMS) was purchased from Eurisotop (Cheshire, UK).

### 3.2. Methods

#### 3.2.1. Synthesis of Amino Acid Alkyl Ester Ibuprofenates

**Figure 7:**
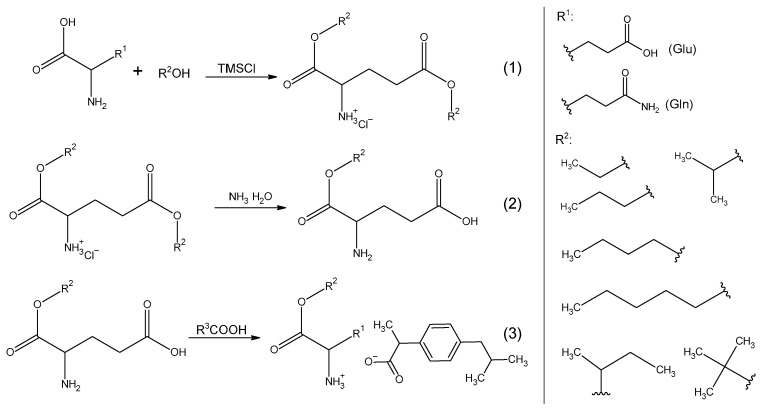
Method for the synthesis of amino acid alkyl ester ibuprofenates.

Amino acid alkyl ester ibuprofenates were obtained as a result of a three-step reaction (Figure 7), which included the esterification and hydrochlorination reaction of the amino acid (Reaction 1), the reaction of neutralization of the amino acid alkyl ester hydrochloride (Reaction 2), and the reaction of protonation of the amino acid alkyl ester using (*RS*)-2-[4-(2-methylpropyl)phenyl]propionic acid (ibuprofen) (Reaction 3). L-amino acids were used for the synthesis: L-glutamic acid and L-glutamine. Amino acids were esterified with alcohols: ethyl, propyl, isopropyl, butyl, *sec*-butyl, *tert*-butyl, and pentyl.

L-amino acid alkyl ester hydrochlorides (Reaction 1) were synthesized at 60 °C under reflux for 24 h. Then, the unreacted alcohol was distilled off on an evaporator at 60 °C under reduced pressure. The product was washed with diethyl ether, dissolved in chloroform, and filtered under reduced pressure. The filtrate was distilled under reduced pressure (60 °C, 10 mBar), and then the obtained product was washed with diethyl ether. The obtained hydrochloride was dried in a vacuum oven at 60 °C and a pressure of 5 mBar for 24 h.

In the next step (Reaction 2), the amino acid alkyl ester hydrochloride was neutralized using a 25% ammonia solution in a 1:3 ratio. Then, 1 cm^3^ of water was added and extracted thrice with diethyl ether (3 × 5 cm^3^). The ether layer was separated from the aqueous layer and dried using anhydrous sodium sulfate. The solvent was distilled off under reduced pressure at 35 °C. Due to the low stability of amino acid alkyl esters, the protonation reaction was immediately carried out after their preparation.

The amino acid alkyl ester ibuprofenates were obtained by reacting the appropriate L-amino acid alkyl ester with (*RS*)-2-[4-(2-methylpropyl)phenyl]propanoic acid. For this purpose, an accurately weighed amino acid alkyl ester was added to an equimolar amount of ibuprofen. The reactions were carried out for approximately 10 min at room temperature in the presence of 5 mL of chloroform. The solvent was then distilled under reduced pressure at 40 °C, and the obtained ibuprofenate was dried in a vacuum dryer at a temperature of 60 °C and a pressure of 5 mBar for 24 h.

#### 3.2.2. Nuclear Magnetic Resonance Spectroscopy (NMR)

NMR spectra of the obtained intermediates and products were examined on a Bruker DPX-400 spectrometer (Billerica, MA, USA) at a frequency of 400 MHz for proton spectra (^1^H NMR) and 100 MHz for carbon spectra (^13^C NMR). The compounds were dissolved in deuterated chloroform (CDCl_3_). TMS (tetramethylsilane) was used as the chemical shift standard. Samples with a concentration of approximately 42 mg cm^−3^ were analyzed.

#### 3.2.3. Fourier Transform Infrared Spectroscopy (FTIR)

Absorption measurements in the infrared range were performed using the Multiple Attenuated Total Reflectance method using a Nicolet 380 Thermo Electron Corporation spectrometer (Waltham, MA, USA). The spectra were recorded in the Omnic 7.3 computer program with an integrated spectrometer. The measurement range was 4000–400 cm^−1^.

#### 3.2.4. UV–Vis Spectroscopic Analysis

Absorption measurements in the UV–Vis range (200–1100 nm) were performed using the Spectroquant Pharo 300 Spectrophotometer from Merk (Darmstadt, Germany). The positions of the absorption bands of the analyzed compounds were recorded for solutions with a concentration of approximately 5 g·dm^−3^ in anhydrous ethanol.

#### 3.2.5. Polarimetric Analysis

Optical rotation measurements were made using an automatic Autopol IV polarimeter from Rudolph Research Analytical (Hackettstown, NJ, USA). The rotation was measured at a wavelength of 589 nm at a temperature of 20 °C. Samples were prepared by dissolving 50 mg of the test compound in 10 cm^3^ of anhydrous ethanol. The sample was placed in a 100 mm long cell.

Specific rotation was calculated from Equation (1):

(1) $$[\alpha {]}_{ʎ}^{T}=\frac{\alpha_{ʎ}^{T}}{c\cdot l}$$

where:

$\alpha_{ʎ}^{T}$—optical rotation at temperature T and wavelength ʎ (°Arc)
$[\alpha {]}_{ʎ}^{T}$—specific rotation at temperature T and wavelength ʎ (°Arc)
c—concentration (g·cm^−3^)
l—cell length (dm^3^).

The specific molar rotation was calculated from Equation (2):

(2) $${\left[M\right]}_{\lambda }^{T}=[\alpha {]}_{ʎ}^{T}\frac{M}{100}$$

where:

M—the molar mass of the compound (g·mol).

#### 3.2.6. Thermogravimetric Analysis (TG)

Thermal stability was assessed using thermogravimetric studies performed on a Netzsch TG 209 F1 Libra thermomicrobalance (Selb, Germany). The test was carried out in an oxidizing environment, with the flow of nitrogen (protective gas): 10 cm^3^·min^−1^, air: 25 cm^3^·min^−1^ in the temperature range from 25 to 1000 °C. Samples weighing approximately 5 mg were heated at 10 °C·min^−1^. The decomposition onset temperature (T_onset_) was calculated using the intersection of the tangents of the TG curve. The fastest decomposition temperatures (T_max_) were calculated using the first TG curve (DTG curve) derivative.

#### 3.2.7. Differential Scanning Calorimetry (DSC)

The phase transition temperatures of the obtained compounds were determined on a TA Instruments device, model Q-100 DSC (New Castle, DE, USA). The sample was placed in an aluminum crucible with a fluted lid. The analysis was performed in a nitrogen atmosphere. The sample was heated from −90 °C to a temperature 10 °C lower than the sample decomposition onset temperature (determined individually for each compound, determined based on TG analysis) with an increase of 10°·min^−1^.

#### 3.2.8. X-ray Diffraction (XRD)

X-ray diffraction (XRD) was performed using an AERIS PANalytical X-ray diffractometer with Cu-K radiation.

#### 3.2.9. Partition Coefficient n-octanol/Water

For L-glutamic acid alkyl ester ibuprofenates, the partition coefficient was determined using n-octanol/water extraction. For this purpose, 10 mg of the substance, weighed with an accuracy of 0.01 mg, was mixed with 5 cm^3^ of water saturated with n-octanol and 5 cm^3^ of n-octanol saturated with water. The mixture was stirred vigorously at 25 °C for 3 h and then centrifuged at 7500 rpm·min^−1^ at 25 °C for 10 min to separate the phases. The phases were then separated. The concentration of the compound was determined in the aqueous layer using the HPLC method (standard curve method).

The partition coefficient log P was calculated based on Equation (3):

(3) $$\log P={\log c}_{oct}-{\log c}_{w}$$

where:

c_w_ and c_oct_ are the concentrations (mg·dm^−3^) of the solute in the aqueous and octanol layers.

The concentration of the compound dissolved in octanol was calculated from Equation (4):

(4) $$c_{oct}=c_{0}-c_{w}$$

where:

c_0_ is the concentration of the compound calculated based on sample weights prepared for analysis.

**Figure 8:**
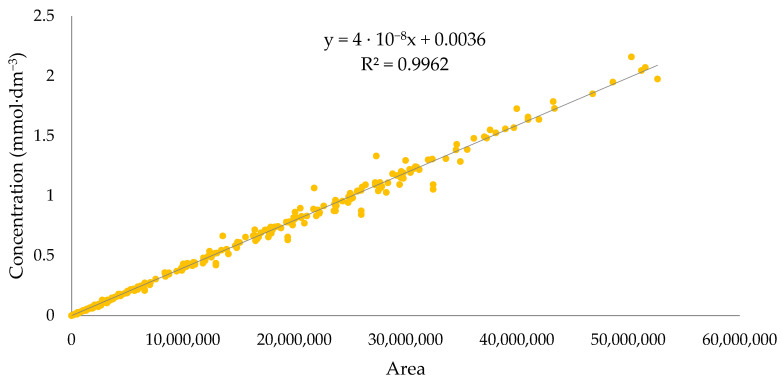
Calibration curve for ibuprofenate alkyl esters.

The concentration of the substance in the aqueous layer was determined using the SHIMADZU Nexera-i LC-2040C 3D High Plus high-performance liquid chromatography method with a DAD/FLD detector. A mixture of 50% acetonitrile and 50% water was used as the moving fluid under isocratic conditions at a 1 cm^3^·min^−1^ flow rate. A Kinetex^®^ 2.6 µm F5 100 Å column heated to 35 °C with dimensions of 150 × 4.6 mm^2^ from Phenomenex was used. The spectra were recorded at a wavelength of 210 nm. The injection volume for these samples was 50 mm^3^. Each measurement was performed three times, and the results were averaged. The concentration of ibuprofen and its salts was calculated from peak area measurements using the calibration curve method (Figure 8). The results were analyzed using LabSolutions/LC Solution System.

#### 3.2.10. Solubility in Water

Water solubility was determined by obtaining saturated solutions of the obtained ibuprofenates. For this purpose, the appropriate amount of ibuprofen salt was weighed in a vial, poured with deionized water, and stirred vigorously on a magnetic stirrer at 25 °C for 24 h. After this time, the mixture was filtered using a syringe filter made of MCE (mixed cellulose esters) with a pore diameter 0.45 µm. Then, the filtrate was diluted one hundred times with acetonitrile: water solution (1:1). The concentration of the compound was determined by the HPLC method described in Section 3.2.9.

#### 3.2.11. Permeability of the Active Substance through Pig Skin—In Vitro Study

Tests for the penetration of the active substance through pig skin were performed using Franz diffusion chambers (Phoenix DB-6, ABL&E-JASCO, Vienna, Austria) with diffusion areas of 1 cm^2^ according to the modified method [15].

Pig skin was used as a membrane in the studies because it has similar structure and permeability parameters to human skin [16,17]. Pig skin obtained from the belly was purchased from a local slaughterhouse. The outer layer, 0.5 mm thick, was then removed using a dermatome and cut into 2 cm × 2 cm pieces. Before use in the study, the skin was soaked in buffered saline (PBS) solution at pH 7.4.

Skin impedance assessment was performed using an LCR meter 4080 (Voltcraft LCR 4080, Conrad Electronic, Hirschau, Germany), which was operated in parallel mode at an alternating frequency of 120 Hz (error for kΩ values < 0.5%). The measurements were made by placing the tips of the measurement probes in the donor and acceptor chambers, separated by a skin sample. Both chambers were filled with PBS buffer pH 7.4. Skin samples with an impedance > 3 kΩ were used for testing, which corresponds to the electrical resistance of human skin [15,18].

The acceptor chamber with a capacity of 10 cm^3^ was filled with PBS solution (pH 7.4). A total of 1 cm^3^ of a solution of ibuprofen or L-amino acid alkyl ester ibuprofenate with a concentration of 1% based on the active substance (ibuprofen) in 70% ethanol was placed in the acceptor chamber.

The experiment was conducted for 24 h at 37 °C. After 0.5 h, 1 h, 2 h, 3 h, 4 h, 5 h, 8 h, and 24 h from the start of the permeation experiment, 0.5 cm^3^ samples of the acceptor fluid were taken, respectively, and refilled with fresh PBS solution. The content of ibuprofen or its salts in the acceptor liquid was determined by HPLC. Based on this concentration, the cumulative mass expressed as ibuprofen was calculated (µg IBU·cm^−2^) [3,19,20,21,22].

The process of substance absorption through the layers of the epidermis can be described by Fick’s first law of diffusion [21,22,23,24]. The constant flow coefficient (in µg IBU·cm^−2^·h^−1^) through the pig skin into the acceptor fluid was determined as the slope of the plot of accumulated mass in the acceptor fluid as a function of time.

The permeation parameters, i.e., the flux of ibuprofen and its derivatives through the skin (J_ss_), the permeability coefficient (K_p_), and the time required to achieve steady-state permeation (lag time—L_T_), were determined from the permeation profile using Equation (5):

(5) $$A=J_{ss}(t-L_{T})$$

where:

A—accumulated mass of the active ingredient [µg IBU·cm^−2^],
J_ss_—steady-state flux [µg·cm^−2^·h^−1^]
t—time [h]
L_T_—lag time [h].

Steady-state flow was estimated from the slope of the linear part of the plot of accumulated mass in the acceptor phase over time. The lag time (L_T_) was determined based on the x-intercept of the linear part of the graph of accumulated mass in the acceptor phase over time. It was then used to calculate the permeability coefficient K_p_ using Equation (6):

(6) $$K_{p}=\frac{J_{ss}}{C}$$

where:

C—concentration in the donor phase (µg·cm^−3^).

The diffusion coefficient (D) was calculated based on Equation (7):

(7) $$D=\frac{I^{2}}{6\cdot L_{T}}$$

where:

I—diffusion path, skin thickness (mm).

The skin partition coefficient (K_m_) was calculated based on Equation (8):

(8) $$K_{m}=\frac{K_{p}\cdot I}{D}$$

#### 3.2.12. Accumulation in the Skin—In Vitro Study

After completing the permeation test, the skin was removed from the Franz chamber, then washed with 0.5% sodium lauryl sulfate solution and dried with a paper towel. After being thoroughly air-dried, the leather was cut into pieces and extracted with 2 cm^3^ of methanol for 24 h at 4 °C. Then, the samples were homogenized using a homogenizer (IKA^®^T18 digital ULTRA TURRAX, Staufen, Germany) for 3 min and centrifuged at 3500 rpm·min^−1^ for 5 min. After centrifugation, the solution was analyzed by HPLC. The accumulation of IBU and its salts in pig skin was calculated by dividing the amount of substance remaining in the samples by the weight of the skin sample and it was expressed as the weight of IBU per gram of skin (µg IBU·g^−1^).

## 4. Conclusions

This study systematically investigated the impact of introducing L-glutamic acid alkyl ester moieties into the ibuprofen structure, shedding light on their physicochemical properties and skin permeability behavior. The obtained L-glutamic acid alkyl ester ibuprofenates exhibited a decrease in lipophilicity, as evidenced by the lower octanol/water partition coefficients ranging from 2.374 to 3.191 compared to ibuprofen. This reduction in lipophilicity was attributed to the incorporation of the amino acid alkyl ester cation, indicating that these compounds can be classified as medium-lipophilic substances.

Furthermore, the ibuprofen salts displayed significantly enhanced solubility in water compared to the parent ibuprofen. Among them, [Glu(OiPr)][IBU] exhibited the highest solubility, approximately three times higher than that of ibuprofen. This enhanced solubility is important for pharmaceutical applications, as it can potentially lead to improved bioavailability and efficacy.

Additionally, the skin permeability studies revealed that the L-glutamic acid alkyl ester ibuprofenates exhibited higher permeation rates through pig skin compared to ibuprofen. The compound [Glu(OPent)][IBU], demonstrated the highest skin penetration value after 24 h, indicating its potential as a promising transdermal delivery system. Interestingly, it was observed that compounds with higher permeability rates also showed lower accumulation in the skin. This finding suggests that increased permeability might lead to reduced skin retention, which could be advantageous for minimizing localized side effects.

Furthermore, the study highlighted a relationship between the length of the alkyl chain and skin accumulation. As the length of the alkyl chain increased, the accumulation in the skin also increased until it reached a maximum at the butyl chain, after which it decreased. This observation provides valuable insights for the design of transdermal drug delivery systems, allowing for the optimization of skin permeability and reduced skin retention.

In summary, the incorporation of L-glutamic acid alkyl ester moieties into the ibuprofen structure not only enhanced the water solubility of the compound but also significantly improved its skin permeability properties. These findings have important implications for the development of novel transdermal drug delivery systems, offering potential advancements in the field of pharmaceutical sciences. Further research could focus on exploring the therapeutic applications of these ibuprofen salts and their efficacy in vivo, paving the way for innovative and efficient drug delivery strategies.

## Figures and Tables

**Table 1:** Optical, specific, and molar rotation of L-glutamic acid, hydrochlorides, and ibuprofenates of L-glutamic acid alkyl esters.

Compound	Molar Mass (g·mol^−1^)	Concentration (g·dm^−3^)	Optical Rotation $\alpha_{\lambda }^{T}$ (Arc°)	Specific Rotation ${\left[\alpha \right]}_{\lambda }^{T}$	Molar Rotation ${\left[M\right]}_{\lambda }^{T}$
Glu	147.13	50.1	+0.057	+11.377	+16.739
[Glu(OEt)_2_][HCl]	239.70	48.6	+0.106	+20.990	+50.313
[Glu(OPr)_2_][HCl]	267.75	50.2	+0.102	+20.319	+54.404
[Glu(OiPr)_2_][HCl]	267.75	54.8	+0.130	+23.723	+63.518
[Glu(OBu)_2_][HCl]	295.80	47.6	+0.081	+15.842	+46.861
[Glu(O*sec*-Bu)_2_][HCl]	295.80	49.5	+0.100	+20.040	+59.278
[Glu(OPent)_2_][HCl]	323.86	49.9	+0.087	+17.435	+56.465
[Glu(OEt)][IBU]	409.51	52.4	+0.012	+2.099	+8.696
[Glu(OPr)][IBU]	437.56	52.6	+0.010	+1.901	+8.318
[Glu(OiPr)][IBU]	437.56	52.0	+0.005	+9.804	+42.898
[Glu(OBu)_2_][IBU]	465.61	51.7	+0.012	+1.998	+9.303
[Glu(O*sec*-Bu)_2_][IBU]	465.61	53.3	+0.006	+1.126	+5.243
[Glu(OPent)_2_][IBU]	493.67	58.0	+0.006	+1.186	+5.855

**Table 2:** Thermal stability, melting points, and glass transition for hydrochlorides and ibuprofenates of L-glutamic acid alkyl esters.

Compound	T_onset_ (°C)	T_max_ (°C)	T_m_ (°C) (ΔH_m_ (J·g^−1^))	T_g_ (°C)
[Glu(OEt)_2_][HCl]	168.3	193.3	100.90 (61.300)	−25.07
[Glu(Opr)_2_][HCl]	170.5	193.4	91.12 (68.596)	−34.59
[Glu(OiPr)_2_][HCl]	174.4	202.5	45.72 (11.887)	−22.13
[Glu(OBu)_2_][HCl]	172.8	192.1	63.47 (44.402)	−41.14
[Glu(O*sec*-Bu)_2_][HCl]	181.5	207.5	51.69 (29.590)	−38.75
[Glu(OPent)_2_][HCl]	169.5	189.6	69.24 (54.587)	−44.82
[Glu(OEt)][IBU]	194.2	228.3	50.43 (16.867)	−49.89
[Glu(OPr)][IBU]	199.7	230.8	59.02 (33.058)	−48.94
[Glu(OiPr)][IBU]	222.9	239.7	-	−47.53
[Glu(OBu)][IBU]	206.8	247.7	71.05 (1.983)	−52.49
[Glu(O*sec*-Bu)][IBU]	204.2	241.9	-	−48.42
[Glu(OPent)][IBU]	206.9	245.1	-	−53.54

T_onset_, the onset temperature of the thermal degradation; T_max_, the temperature of the fastest decomposition; T_m_, melting point; ΔH_m_, melting enthalpy; T_g_, glass transition temperature.

**Table 3:** Partition coefficient n-octanol/water and solubility of ibuprofen and L-glutamic acid alkyl ester ibuprofenates in water at 25 °C.

Compound	log P	Solubility in Water	
		(g·dm^−3^)	(g IBU·dm^−3^)
IBU	3.370 ± 0.000	0.075 ± 0.001	0.075 ± 0.001
[Glu(OEt)][IBU]	2.374 ± 0.001	0.185 ± 0.003	0.100 ± 0.001
[Glu(OPr)][IBU]	2.380 ± 0.003	0.230 ± 0.007	0.120 ± 0.004
[Glu(OiPr)][IBU]	2.860 ± 0.007	0.390 ± 0.011	0.203 ± 0.006
[Glu(OBu)][IBU]	3.000 ± 0.010	0.217 ± 0.007	0.109 ± 0.004
[Glu(O*sec*-Bu)][IBU]	3.191 ± 0.007	0.185 ± 0.024	0.093 ± 0.012
[Glu(OPent)][IBU]	2.459 ± 0.002	0.208 ± 0.024	0.101 ± 0.012

**Table 4:** Cumulated mass and skin permeation parameters for ibuprofen and L-glutamic acid alkyl ester ibuprofenates; *n* = 3.

Compound	CMP (g IBU·dm^−3^)	J_ss_, (µg IBU·cm^−2^ ·h^−1^)	K_p_·10^4^, (cm·h^−1^)	L_T_, (h)	D·10^4^, (cm^2^·h^−1^)	K_m_	Q%_24h_
IBU	0.075 ± 0.001	26.539 ± 0.320	2.669 ± 0.032	0.153 ± 0.029	27.926 ± 5.802	0.005 ± 0.001	0.302 ± 0.038
[Glu(OEt)][IBU]	0.100 ± 0.001	30.855 ± 6.617	3.267 ± 0.372	0.029 ± 0.050	- *	- *	0.566 ± 0.005
[Glu(OPr)][IBU]	0.120 ± 0.004	26.451 ± 2.253	2.686 ± 0.131	- *	- *	- *	0.506 ± 0.008
[Glu(OiPr)][IBU]	0.203 ± 0.006	23.298 ± 3.459	2.082 ± 0.309	- *	- *	- *	0.373 ± 0.0175
[Glu(OBu)][IBU]	0.109 ± 0.004	32.282 ± 7.809	3.162 ± 0.328	0.102 ± 0.069	71.033 ± 69.953	0.004 ± 0.003	0.452 ± 0.026
[Glu(Osec-Bu)][IBU]	0.093 ± 0.012	22.296 ± 1.398	2.291 ± 0.232	- *	-*	- *	0.456 ± 0.048
[Glu(OPent)][IBU]	0.101 ± 0.012	57.098 ± 29.761	5.267 ± 2.745	- *	- *	- *	0.582 ± 0.047

CPM—cumulative permeation mass; J_SS_—steady-state flux; K_P_—permeability coefficient; L_T_—lag time; D—diffusion coefficient; K_m_—skin partition coefficient; Q%_24h_—the percentage of the applied dose; * these parameters could not be calculated because the value of the intercept from the permeation curve equation needed for the calculations was ≤0.

## Data Availability

Data is contained within the article or Supplementary Material.

## Supplementary Materials

The following supporting information can be downloaded at: https://www.mdpi.com/article/10.3390/molecules28227523/s1, Figure S1. ^1^H NMR spectrum of [Glu(OEt)_2_][HCl]; Figure S2: ^13^C NMR spectrum of [Glu(OEt)_2_][HCl]; Figure S3: ^1^H NMR spectrum of [Glu(OPr)_2_][HCl]; Figure S4: ^13^C NMR spectrum of [Glu(OPr)_2_][HCl]; Figure S5: ^1^H NMR spectrum of [Glu(OiPr)_2_][HCl]; Figure S6: ^13^C NMR spectrum of [Glu(OiPr)_2_][HCl]; Figure S7: ^1^H NMR spectrum of [Glu(OBu)_2_][HCl]; Figure S8: ^13^C NMR spectrum of [Glu(OBu)_2_][HCl]; Figure S9: ^1^H NMR spectrum of [Glu(O*sec*-Bu)_2_][HCl]; Figure S10: ^13^C NMR spectrum of [Glu(O*sec*-Bu)_2_][HCl]; Figure S11: ^1^H NMR spectrum of [Glu(OPent)_2_][HCl]; Figure S12: ^13^C NMR spectrum of [Glu(OPent)_2_][HCl]; Figure S13: ^1^H NMR spectrum of [Glu(OEt)][IBU]; Figure S14: ^13^C NMR spectrum of [Glu(OEt)][IBU]; Figure S15: ^1^H NMR spectrum of [Glu(OPr)][IBU]; Figure S16: ^13^C NMR spectrum of [Glu(OPr)][IBU]; Figure S17: ^1^H NMR spectrum of [Glu(OiPr)][IBU]; Figure S18: ^13^C NMR spectrum of [Glu(OiPr)][IBU]; Figure S19: ^1^H NMR spectrum of [Glu(OBu)][IBU]; Figure S20: ^13^C NMR spectrum of [Glu(OBu)][IBU]; Figure S21: ^1^H NMR spectrum of [Glu(O*sec*-Bu)][IBU]; Figure S22: ^13^C NMR spectrum of [Glu(O*sec*-Bu)][IBU]; Figure S23: ^1^H NMR spectrum of [Glu(OPent)][IBU]; Figure S24: ^13^C NMR spectrum of [Glu(OPent)][IBU]; Figure S25: FTIR spectrum of [Glu(OEt)_2_][HCl]; Figure S26: FTIR spectrum of [Glu(OPr)_2_][HCl]; Figure S27. FTIR spectrum of [Glu(OBu)_2_][HCl]; Figure S28: FTIR spectrum of [Glu(O*sec*-Bu)_2_][HCl]; Figure S29: FTIR spectrum of [Glu(OPent)_2_][HCl]; Figure S30. FTIR spectrum of [Glu(OEt)][IBU]; Figure S31: FTIR spectrum of [Glu(OPr)][IBU]; Figure S32: FTIR spectrum of [Glu(OBu)][IBU]. Figure S33: FTIR spectrum of [Glu(O*sec*-Bu)][IBU]; Figure S34: FTIR spectrum of [Glu(OPent)][IBU]; Figure S35: XRD pattern of [Glu(OEt)_2_][HCl]; Figure S36: XRD pattern of [Glu(OPr)_2_][HCl]; Figure S37: XRD pattern of [Glu(OBu)_2_][HCl]; Figure S38: XRD pattern of [Glu(O*sec*-Bu)_2_][HCl]; Figure S39: XRD pattern of [Glu(OPent)_2_][HCl]; Figure S40: XRD pattern of [Glu(OEt)][IBU]; Figure S41: XRD pattern of [Glu(OPr)][IBU]; Figure S42: XRD pattern of [Glu(OBu)][IBU]; Figure S43: XRD pattern of [Glu(O*sec*-Bu)][IBU; Figure S44: XRD pattern of [Glu(OPent)][IBU}; Figure S45: TG, DTG, c-DTA curves of [Glu(OEt)_2_][HCl]; Figure S46: TG, DTG, c-DTA curves of [Glu(OPr)_2_][HCl]; Figure S47: TG, DTG, c-DTA curves of [Glu(OiPr)_2_][HCl]; Figure S48: TG, DTG, c-DTA curves of [Glu(OBu)_2_][HCl]; Figure S49: TG, DTG, c-DTA curves of [Glu(OBu)_2_][HCl]; Figure S50: TG, DTG, c-DTA curves of [Glu(O*sec*-Bu)_2_][HCl]; Figure S51: TG, DTG, c-DTA curves of [Glu(OPent)_2_][HCl]; Figure S52: TG, DTG, c-DTA curves of [Glu(OEt)][IBU]; Figure S53: TG, DTG, c-DTA curves of [Glu(OEt)][IBU]; Figure S54: TG, DTG, c-DTA curves of [Glu(OiPr)][IBU]; Figure S55: TG, DTG, c-DTA curves of [Glu(OBu)][IBU]; Figure S56: TG, DTG, c-DTA curves of [Glu(O*sec*-Bu)][IBU]; Figure S57: TG, DTG, c-DTA curves of [Glu(OPent)][IBU];Figure S58: Summary of DSC thermograms for L-glutamic acid alkyl ester hydrochlorides [Glu(OR)_2_][HCl]; Figure S59: Summary of DSC thermograms for L-glutamic acid alkyl ester ibuprofenates [Glu(OR)][IBU].
