# Supplementary material for: Exploring Alkyl Ester Salts of L-Amino Acid Derivatives of Ibuprofen: Physicochemical Characterization and Transdermal Potential
Source: Molecules. 2023 Nov 10;28(22):7523. doi: 10.3390/molecules28227523 (PMC10673250; doi:10.3390/molecules28227523)
Supplement: Supplementary file 1 [file molecules-28-07523-s001.zip › molecules-2699496-supplementary.pdf]

molecules

MDPI

Supplementary Materials

Exploring Alkyl Ester Salts of L-Amino Acid Derivatives of Ibuprofen: Physicochemical Characterization and Transdermal Potential

Kordian Witkowski [1], Anna Nowak [2], Wiktoria Duchnik [2], Łukasz Kucharski [2], Łukasz Struk [3] and Paula Ossowicz-Rupniewska [1,*]

[1] Department of Chemical Organic Technology and Polymeric Materials, Faculty of Chemical Technology and Engineering, West Pomeranian University of Technology in Szczecin, Piastów Ave. 42, 71-065 Szczecin, Poland
[2] Department of Cosmetic and Pharmaceutical Chemistry, Pomeranian Medical University in Szczecin, Powstańców Wielkopolskich Ave. 72, 70-111 Szczecin, Poland
[3] Department of Organic and Physical Chemistry, Faculty of Chemical Technology and Engineering, West Pomeranian University of Technology in Szczecin, Al. Piastów 42, 71-065 Szczecin, Poland; lukasz.struk@zut.edu.pl
* Correspondence: possowicz@zut.edu.pl; Tel.: +48-4494801

NMR signal assignments of all new compounds - glutamic acid alkyl ester hydrochlorides and ibuprofenates

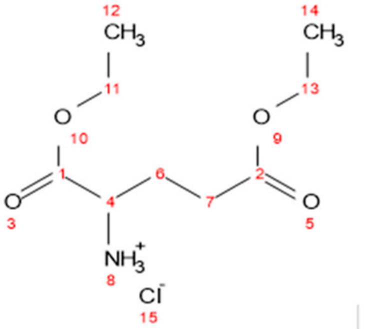

[Glu(OEt)₂][HCl]

^{1}H NMR (400 MHz, $CDCl_3$) δ 8.63 (s, 3H, H8), 4.34 – 4.06 (m, 5H, H4, H11, H13), 2.77 – 2.57 (m, 2H, H7), 2.40 (d, *J* = 7.1 Hz, 2H, H6), 1.28 (dt, *J* = 24.2, 7.1 Hz, 6H, H12, H14).
^{13}C NMR (100 MHz, $CDCl_3$) δ 175.65 (C1), 169.26 (C2), 62.73 (C11), 60.82 (C13), 52.60 (C4), 30.37 (C6), 30.00 (C7), 14.15 (C12), 14.01 (C14).

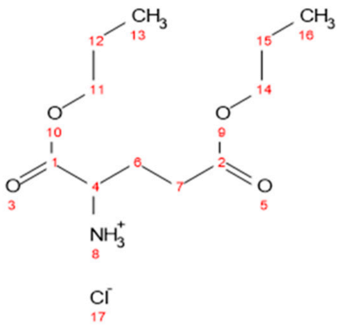

[Glu(OPr)₂][HCl]

^{1}H NMR (400 MHz, $CDCl_3$)δ 8.85 (s, 3H, H8), 4.28 (t, J = 6.5 Hz, 1H, H4), 4.17 (t, J = 6.7 Hz, 2H, H11), 4.07 – 3.98 (m, 2H, H14), 2.68 (ddt, J = 46.8, 17.1, 7.5 Hz, 2H, H6), 2.41 (dt, J = 11.8, 7.5 Hz, 2H, H7), 1.67 (dh, J = 25.3, 7.2 Hz, 4H, H12, H15), 0.94 (dt, J = 10.7, 7.4 Hz, 6H, H13, H16).
^{13}C NMR (100 MHz, $CDCl_3$) δ 172.36 (C1), 169.02 (C2), 68.24 (C11), 66.49 (C14), 52.56 (C4), 29.94 (C6), 25.49 (C7), 21.89 (C12), 21.76(C15), 10.38 (C13), 10.30 (C16).

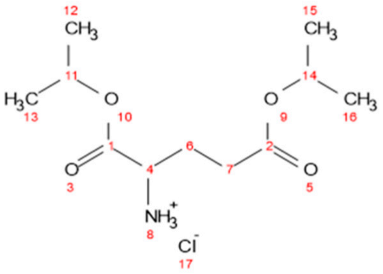

[Glu(OiPr)$_2$][HCl]

^{1}H NMR (400 MHz, $CDCl_3$) δ 8.70 (s, 3H, H8), 5.15 – 5.06 (m, 1H, H11), 5.03 – 4.94 (m, 1H, H4), 4.24 (t, J = 6.4 Hz, 1H, H14), 2.72 – 2.51 (m, 2H, H7), 2.38 (q, J = 7.2 Hz, 2H, H6), 1.32 – 1.21 (m, 12H, H12, H13, H15, H16).

^{13}C NMR (100 MHz, $CDCl_3$) δ 171.90 (C2), 168.53 (C1), 70.94 (C11), 68.34(C14), 52.65 (C4), 30.26 (C7), 25.44 (C6), 21.80 (C12, C13), 21.69 (C15), 21.68 (C16).

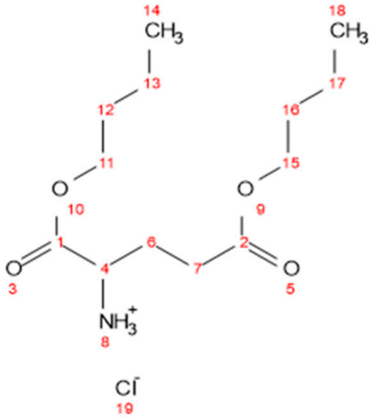

[Glu(OBu)$_2$][HCl]

^{1}H NMR (400 MHz, $CDCl_3$) δ 8.80 (s, 3H, H8), 4.29 (t, J = 6.5 Hz, 1H, H4), 4.20 (td, J = 6.8, 2.1 Hz, 2H, H11), 4.06 (td, J = 6.7, 1.3 Hz, 2H, H15), 2.78 – 2.55 (m, 2H, H7), 2.46 – 2.35 (m, 2H, H6), 1.69 – 1.55 (m, 4H, H12, H16), 1.44 – 1.30 (m, 4H, H13, H17), 0.93 (td, J = 7.4, 2.9 Hz, 6H, H14, H18).

^{13}C NMR (100 MHz, $CDCl_3$) δ 172.36 (C1), 169.15 (C2), 77.27 (C11), 66.53 (C15), 64.73 (C4), 52.58 (C7), 30.57 (C6), 30.34 (C12), 29.96 (C16), 19.09 (C13), 19.01 (C17), 13.71 (C14), 13.65 (C18).

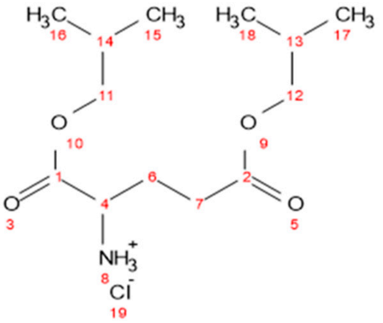

[Glu(O-sec-Bu)$_2$][HCl]

^{1}H NMR (400 MHz, $CDCl_3$) δ 8.79 (s, 1H, H6), 5.02 – 4.89 (m, 1H, H11), 4.82 (p, J = 6.4 Hz, 1H, H15), 4.24 (t, J = 6.7 Hz, 1H, H4), 2.76 – 2.52 (m, 2H, H, H6), 2.38 (q, J = 7.3 Hz, 2H, H7), 1.73 – 1.45 (m, 4H, H13, H17), 1.33 – 1.13 (m, 6H, H12, H16), 0.98 – 0.82 (m, 6H, H14, H18).

^{13}C NMR (100 MHz, $CDCl_3$) δ 171.95 (C1), 168.66 (C2), 77.27(C11), 75.42 (C15), 72.87(C4), 52.69 (C4), 30.21 (C6), 28.55 (C7), 25.58 (C17), 19.33 (C12), 19.19 (C14), 9.62 (C16), 9.58 (C18).

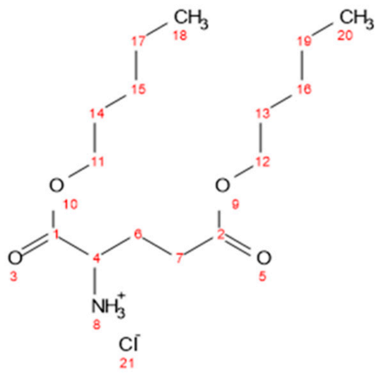

[Glu(OPent)$_2$][HCl]

^{1}H NMR (400 MHz, $CDCl_3$) δ 8.82 (s, 3H, H8), 4.30 – 4.03 (m, 5H, H4, H11, H16), 2.77 – 2.57 (m, 2H, H6), 2.41 (q, J = 7.8 Hz, 2H, H7), 1.64 (dq, J = 21.2, 7.0 Hz, 4H, H12, H17), 1.32 (dh, J = 11.3, 5.8 Hz, 8H, H13, H14, H18, H19), 0.94 – 0.86 (m, 6H, H15, H20).

^{13}C NMR (100 MHz, $CDCl_3$) δ 172.34 (C2), 169.13 (C1), 66.81 (C11), 65.03 (C12), 52.56 (C4), 29.95 (C7), 28.23 (C6), 28.02 (C14), 28.00 (C13), 27.86 (C15), 25.45 (C16), 22.32 (C17), 22.25 (C19), 13.96 (C18), 13.93 (C20).

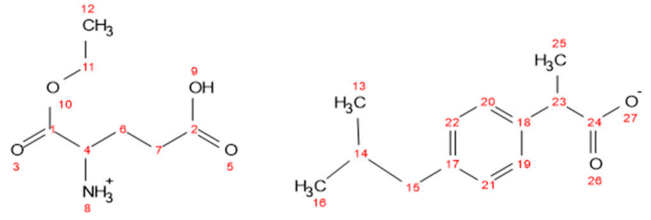

[Glu(OEt)][IBU]

^{1}H NMR (400 MHz, $CDCl_3$) δ 7.26 – 7.19 (m, 2H, H19, H20), 7.13 – 7.06 (m, 2H, H21, H22), 4.28 – 4.15 (m, 3H, H4, H11), 3.70 (q, J = 7.1 Hz, 1H, H23), 2.51 – 2.33 (m, 5H, H6, H7, H15), 2.26 – 2.15 (m, 1H, H15), 1.90 – 1.78 (m, 1H, H14), 1.49 (d, J = 7.2 Hz, 3H, H25), 1.28 (t, J = 7.1 Hz, 3H, H12), 0.89 (d, J = 6.6 Hz, 6H, H13, H16).

^{13}C NMR (100 MHz, $CDCl_3$) δ 179.54 (C24), 179.38 (C2), 171.86 (C1), 140.64 (C18), 137.50 (C17), 129.35 (C19, C20), 127.29 (C21, C22), 61.74 (C11), 55.86 (C4), 45.07 (C15), 45.06 (C15), 45.03 (C23), 30.19 (C7), 29.41 (C14), 24.65 (C6), 22.42 (C13, C16), 18.28 (C25), 14.12 (C12).

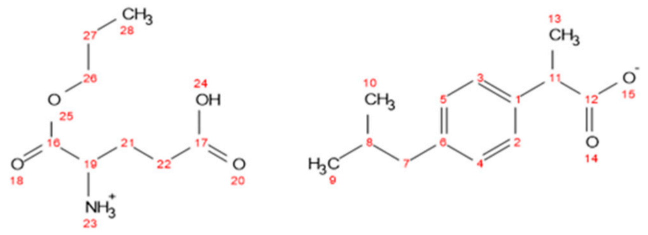

[Glu(OPr)][IBU]

^{1}H NMR (400 MHz, $CDCl_3$) δ 7.22 (s, 2H, H2, H3), 7.10 (d, J = 8.1 Hz, 2H, H4, H5), 4.26 (dd, J = 8.3, 5.0 Hz, 1H, H19), 4.12 (t, J = 6.7 Hz, 2H, H26), 3.71 (q, J = 7.2 Hz, 1H, H11), 2.48 – 2.37 (m, 5H, H22, H21, H7), 2.25 – 2.17 (m, 1H, H21), 1.88 –1.81 (m, 1H, H8), 1.71 – 1.63 (m, 2H, H27), 1.49 (d, J = 7.2 Hz, 3H, H13), 0.95 (t, J = 7.4 Hz, 3H, H28), 0.89 (d, J = 6.6 Hz, 6H, H9, H10).

^{13}C NMR (100 MHz, $CDCl_3$) δ 178.58 (C17), 178.17 (C12), 170.86 (C16), 139.66 (C1), 136.37 (C6), 128.33 (C2, C3), 126.26 (C4, C5), 66.26 (C26), 54.84 (C19), 44.04 (C7), 43.97 (C11), 29.17 (C22), 28.39 (C8), 23.70 (C21), 21.40 (C9, C10), 20.87 (C27), 17.24 (C13), 9.29 (C28).

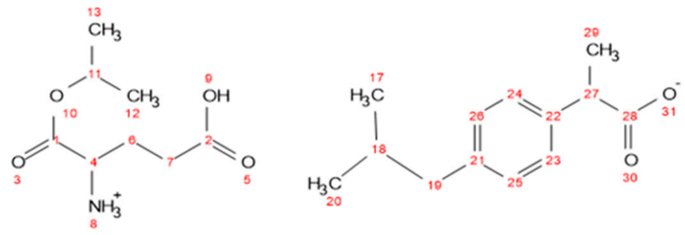

[Glu(iPr)][IBU]

^{1}H NMR (400 MHz, $CDCl_3$) δ 7.23 (d, J = 8.0 Hz, 2H, H23, H24), 7.09 (d, J = 7.9 Hz, 2H, H25, H26), 5.13 – 4.98 (m, 1H, H4), 4.26 – 4.14 (m, 1H, H11), 3.70 (q, J = 7.2 Hz, 1H, H18), 2.49 – 2.31 (m, 5H, H6, H7, H19), 2.23 – 2.13 (m, 1H, H6), 1.92– 1.77 (m, 1H, H27), 1.49 (d, J = 7.2 Hz, 3H, H29), 1.28 – 1.21 (m, 6H, H17, H20), 0.89 (d, J = 6.6 Hz, 6H, H12, H13).

^{13}C NMR (100 MHz, $CDCl_3$) δ 179.26 (C28), 171.34 (C2), 140.63 (C1), 137.53 (C21, C22), 129.34 (C25, C26), 127.29 (C23, C24), 69.45 (C11), 55.96 (C4), 45.06 (C27), 45.03 (C19), 30.19(C7), 29.43 (C18), 24.66 (C6), 22.42 (C17, C20), 21.70 (C12), 21.69 (C13), 18.29 (C29).

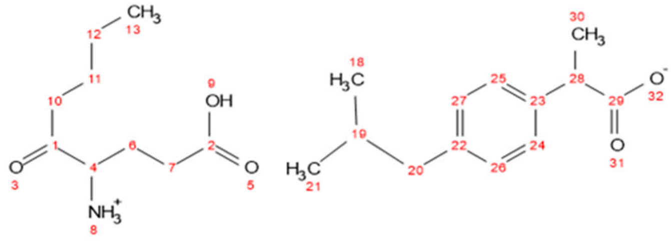

[Glu(OBu)][IBU]

^{1}H NMR (400 MHz, $CDCl_3$) δ 7.23 (d, J = 8.1 Hz, 2H, H24, H25), 7.09 (d, J = 8.1 Hz, 2H, H26, H27), 4.25 (dd, J = 8.1, 5.0 Hz, 1H, H4), 4.16 (t, J = 6.7 Hz, 2H, H11), 3.70 (q, J = 7.1 Hz, 1H, H28), 2.47 – 2.42 (d, m, 3H, H20, H6), 2.42 – 2.33 (m, 2H, H6, H7), 2.24 – 2.16

(m, 1H, H7), 1.88 – 1.79(m, 1H, H30), 1.67– 1.59 (m, 2H, H11), 1.49 (d, J = 7.2 Hz, 3H, H30), 1.41 – 1.34 (m, 2H, H12), 0.94 (t, J = 7.4 Hz, 3H, H13), 0.89 (d, J = 6.6 Hz, 6H, H18, H21).

^{13}C NMR (100 MHz, $CDCl_3$) δ 178.60 (C29), 178.19 (C2), 170.88 (C1), 139.68 (C21), 136.39 (C22), 128.35 (C26, C27), 126.28 (C24, C25), 66.27 (C11), 54.84 (C4), 44.04 (C28), 44.01 (C20), 43.97 (C7), 29.18 (C6), 28.39 (C19), 23.70 (C18), 21.40 (C21), 20.87 (C12), 17.24 (C30), 9.29 (C13).

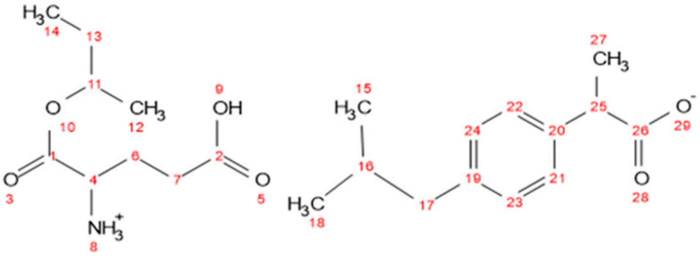

[Glu(O-sec-Bu)][IBU]

^{1}H NMR (400 MHz, $CDCl_3$) δ 7.23 (d, J = 8.1 Hz, 2H, H21, H22), 7.09 (d, J = 8.1 Hz, 2H, H23, H24), 4.89 (p, J = 6.3 Hz, 1H, H4), 4.23 (dd, J = 8.2, 5.0 Hz, 1H, H11), 3.70 (q, J = 7.1 Hz, 1H, H16), 2.47 – 2.42 (m, 3H, H6, H17), 2.42 – 2.32 (m, 2H, H6, H7), 2.22 – 2.14 (m, 1H, H7), 1.84 (dt, J = 13.5, 6.8 Hz, 1H, H11), 1.64 – 1.54 (m, 2H, H13), 1.49 (d, J = 7.2 Hz, 3H, H27), 1.23 (dd, J = 6.3, 1.5 Hz, 3H, H12), 0.90 (dq, J = 7.4, 2.4 Hz, 9H, H14, H15, H18).

^{13}C NMR (100 MHz, $CDCl_3$) δ 179.47 (C26), 179.25 (C2), 171.48 (C1), 140.62(C19,), 137.49 (C20), 129.33 (C21, C22), 73.98 (C23), 73.96 (C24), 56.04 (C11), 45.05 (C4), 36.21 (C25), 30.19 (C17), 29.42 (C7), 28.64 (C16), 24.79 (C6), 24.70 (C13), 22.41 (C18), 19.39 (C15), 19.31 (C12), 18.28 (C27), 9.64 (C14).

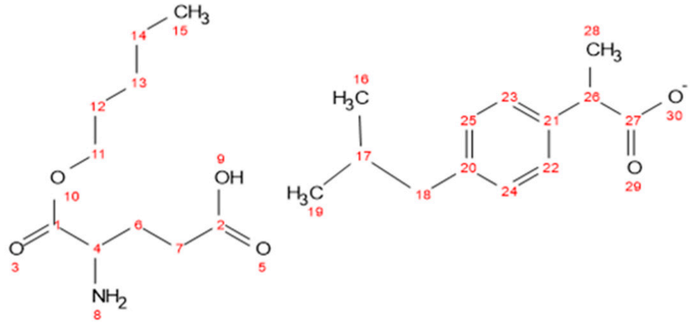

[(Glu(OPent)][IBU]

^{1}H NMR (400 MHz, $CDCl_3$) δ 7.26 – 7.19 (m, 2H, H22, H23), 7.14 – 7.06 (m, 2H, H24, H25), 4.30 – 4.20 (m, 1H, H4), 4.15 (t, J = 6.7 Hz, 2H, H11), 3.70 (q, J = 7.1 Hz, 1H, H26), 2.52 – 2.31 (m, 5H, H6, H7, H18), 2.26 – 2.15 (m, 1H, H6), 1.91 – 1.77 (m, 1H, H17), 1.71 – 1.60 (m, 2H,H12), 1.48 (s, 3H, H28), 1.38 – 1.28 (m, 4H, H13, H14), 0.90 (dd, J = 6.9, 2.6 Hz, 9H, H15, H16, H19).

^{13}C NMR (100 MHz, $CDCl^3$) δ 179.52 (C2), 179.26 (C27), 171.92 (C1), 140.64 (C21), 137.50 (C20), 129.35 (C22, C23), 127.29 (C24, C25), 65.87 (C11), 55.86 (C4), 45.08 (C18), 45.06 (C26), 45.02 (C7), 30.19 (C6), 29.41, 28.17, 27.93, 24.72, 22.42, 22.27, 18.29 (C14), 18.28 (C28), 13.95 (C15).

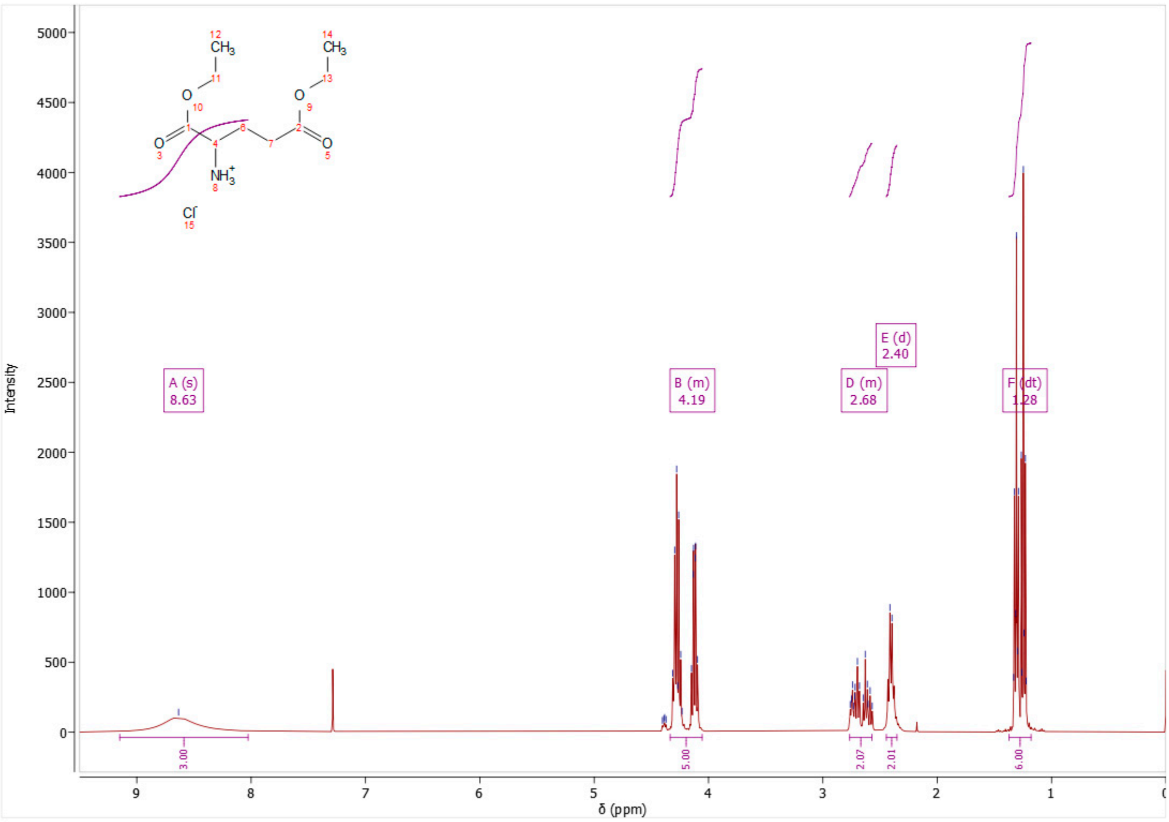

Figure S1. ^{1}H NMR spectrum of [Glu(OEt)2][HCl]

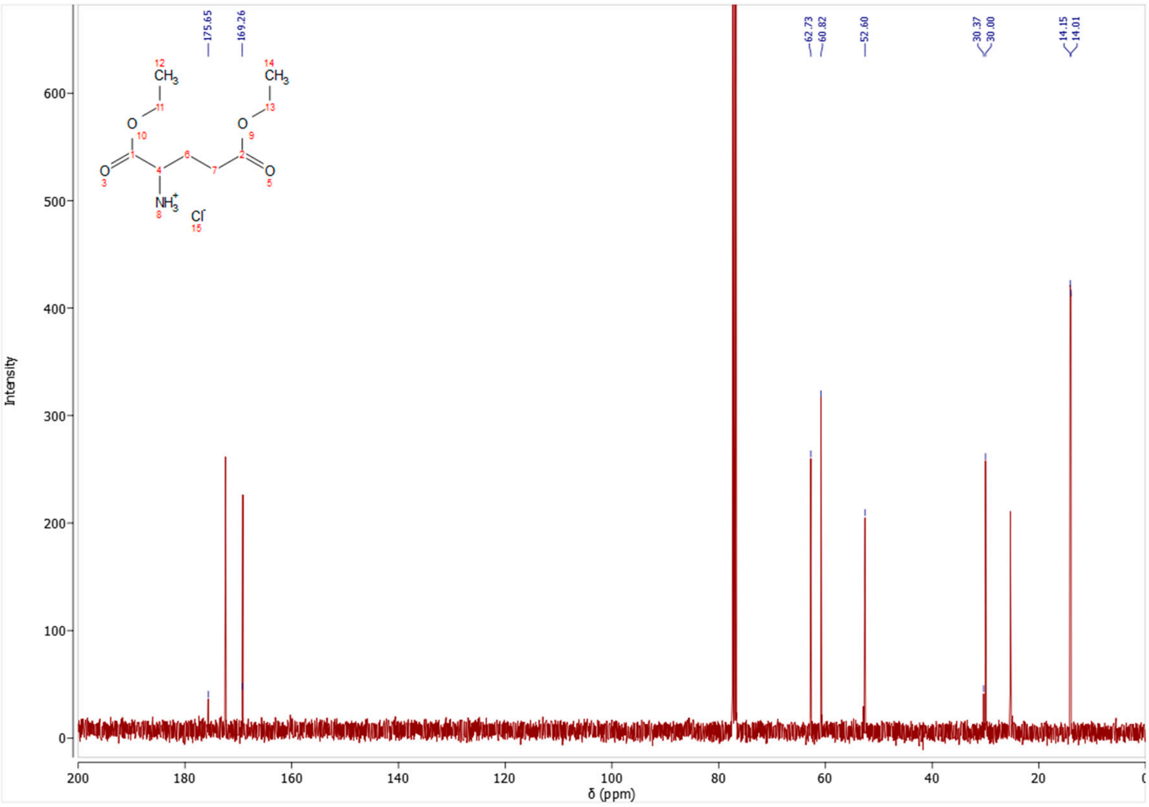

Figure S2. ^{13}C NMR spectrum of [Glu(OEt)2][HCl].

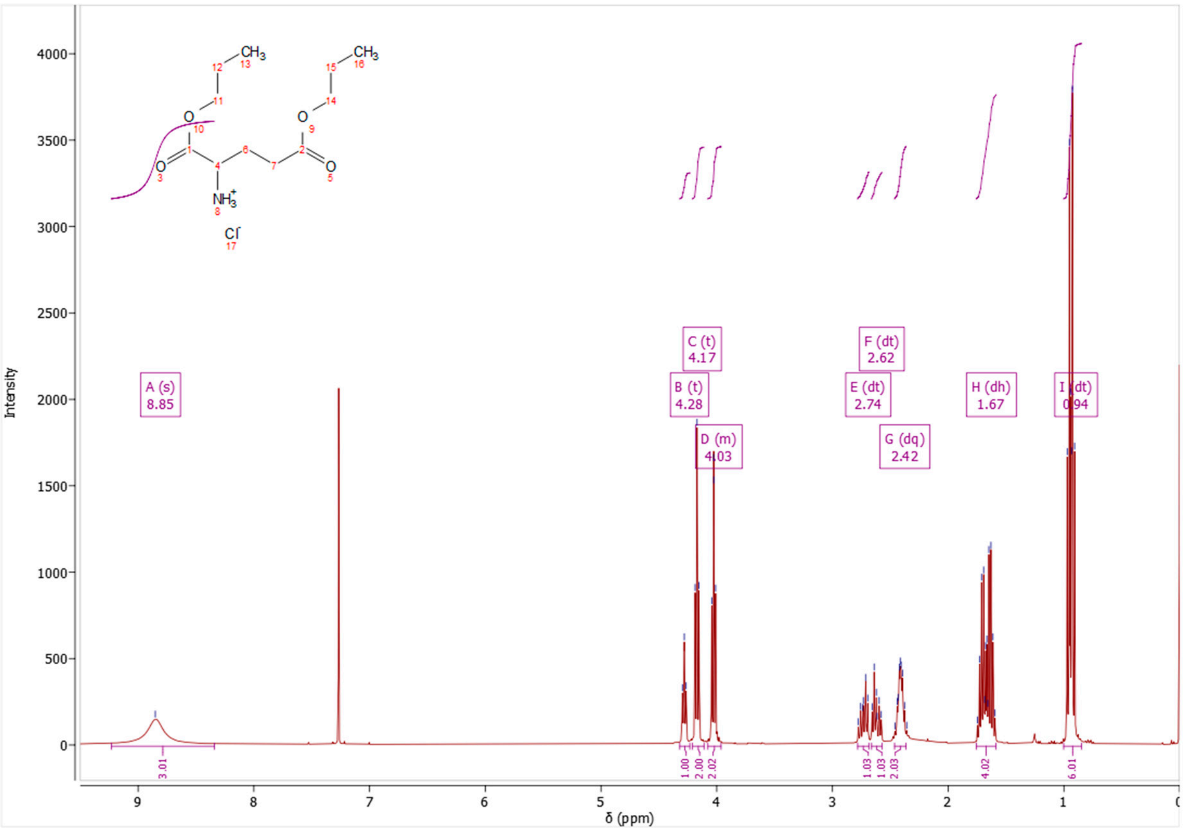

Figure S3. ^{1}H NMR spectrum of [Glu(OPr)$_2$][HCl].

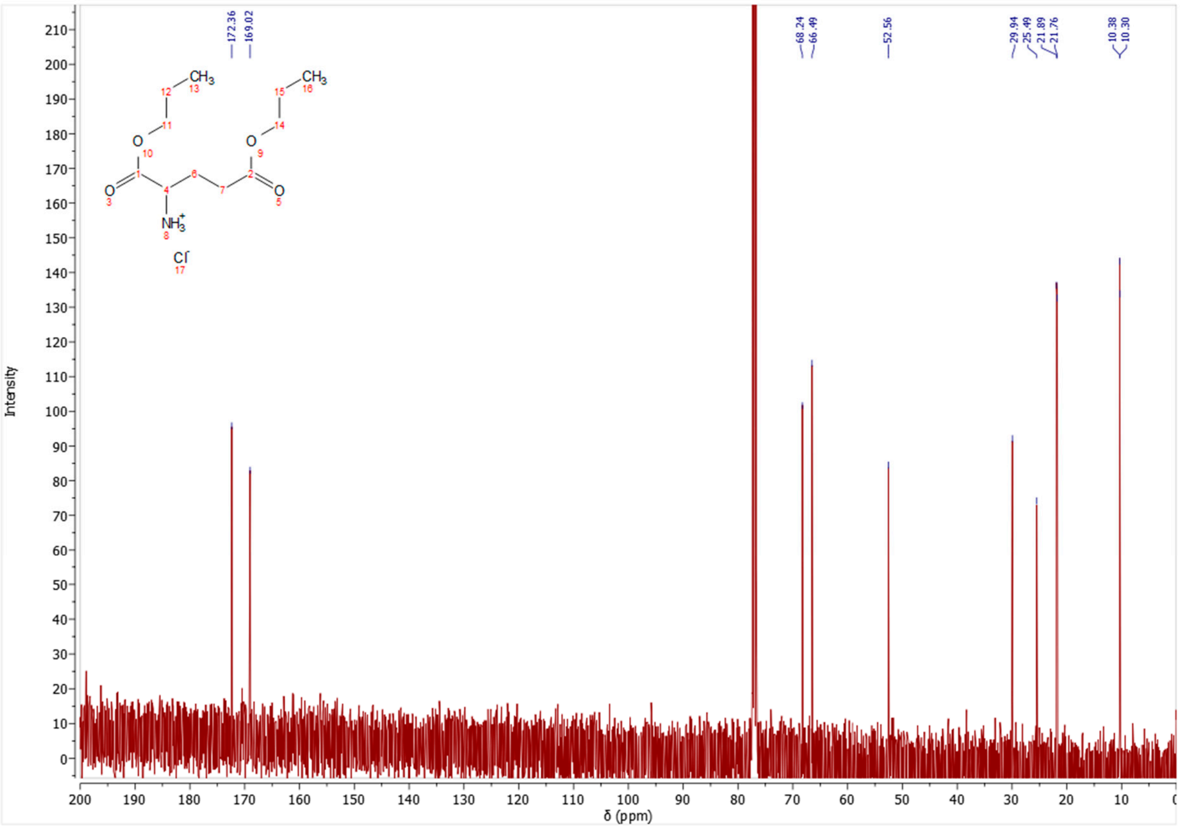

Figure S4. ^{13}C NMR spectrum of [Glu(OPr)$_2$][HCl].

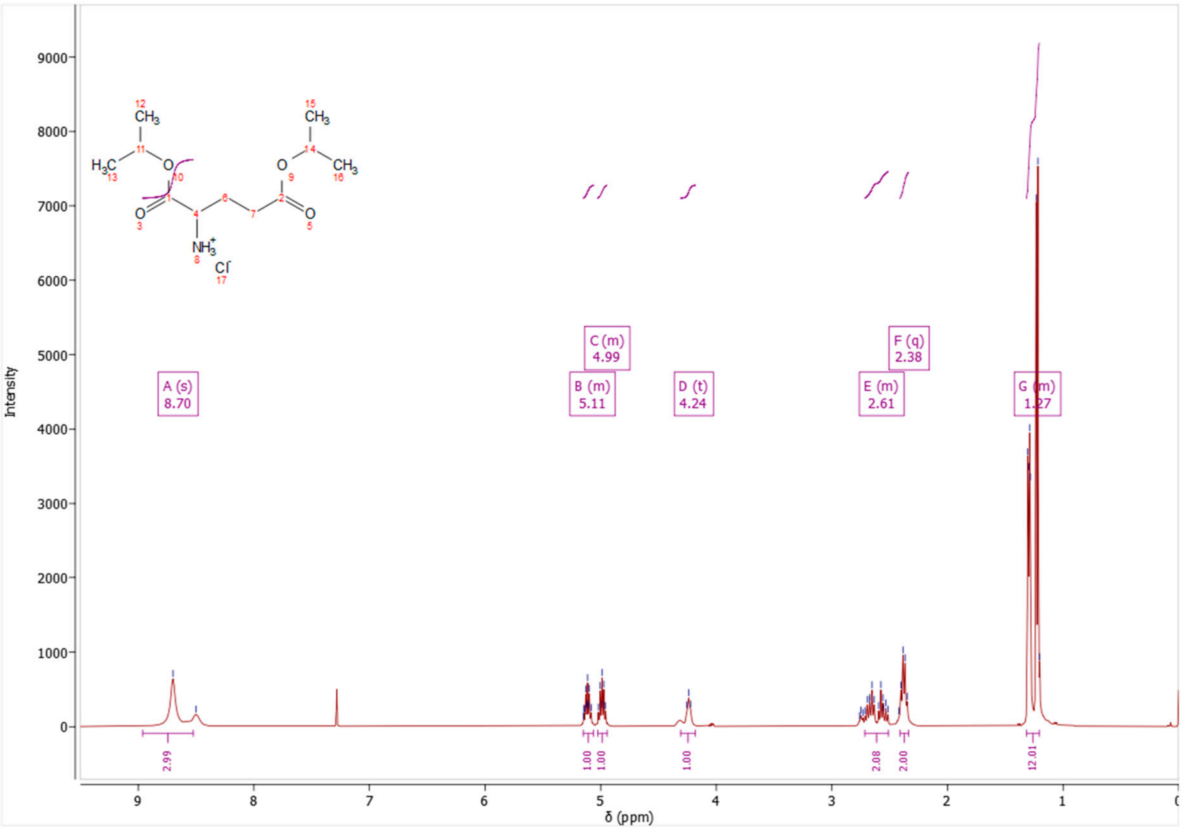

Figure S5. ^{1}H NMR spectrum of $[Glu(OiPr)_2][HCl]$.

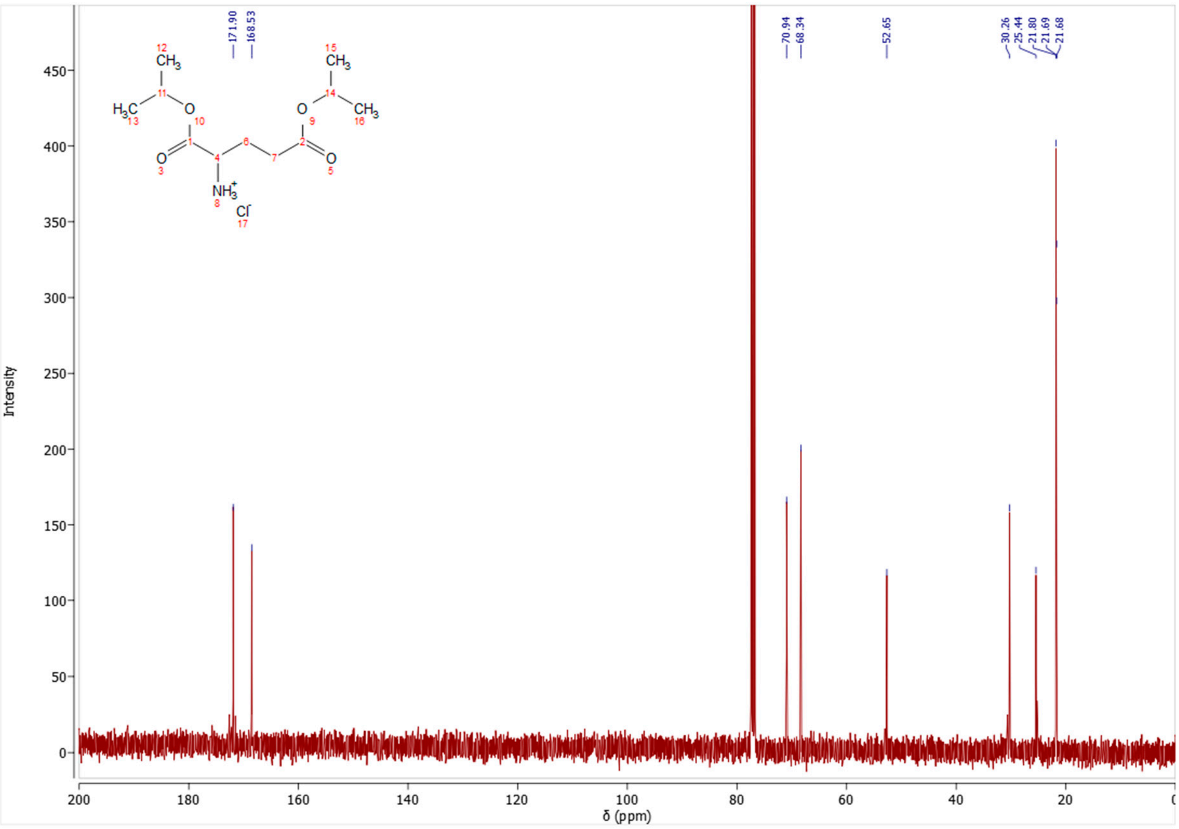

Figure S6. ^{13}C NMR spectrum of $[Glu(OiPr)_2][HCl]$.

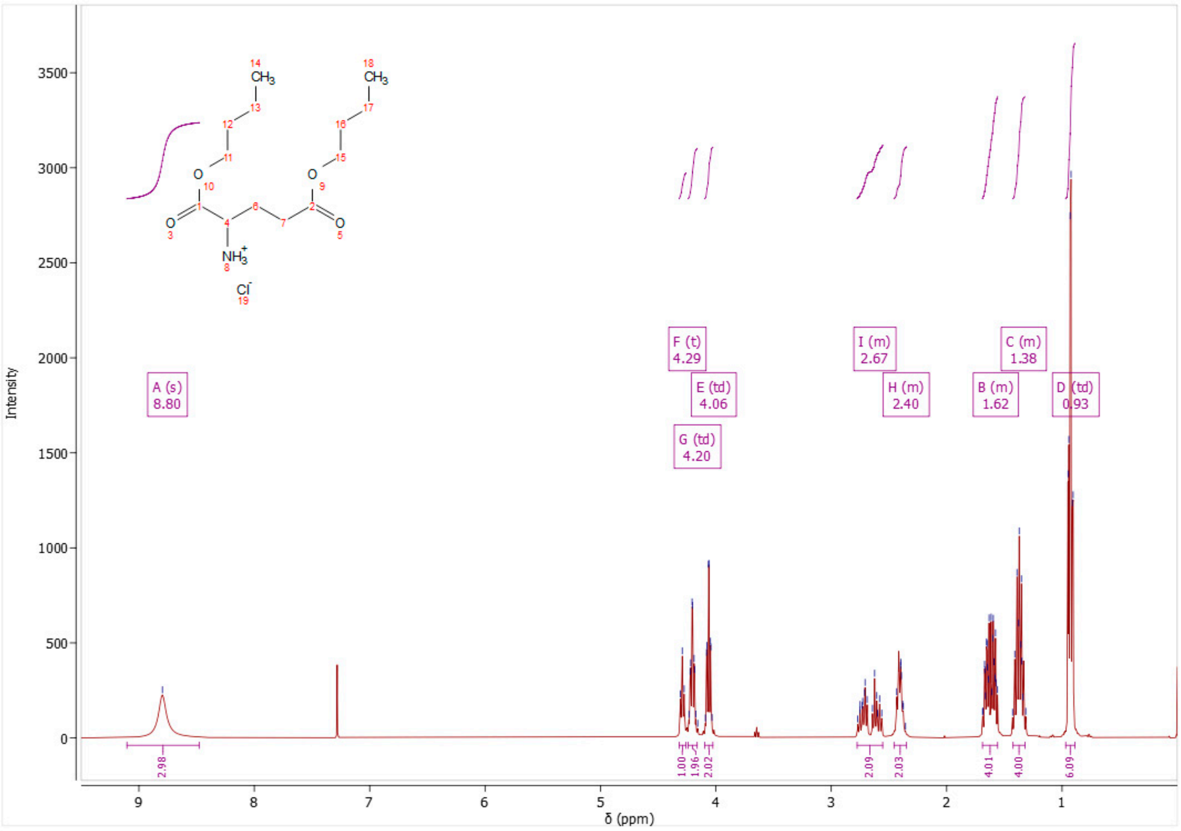

Figure S7. ^{1}H NMR spectrum of [Glu(OBu)$_2$][HCl].

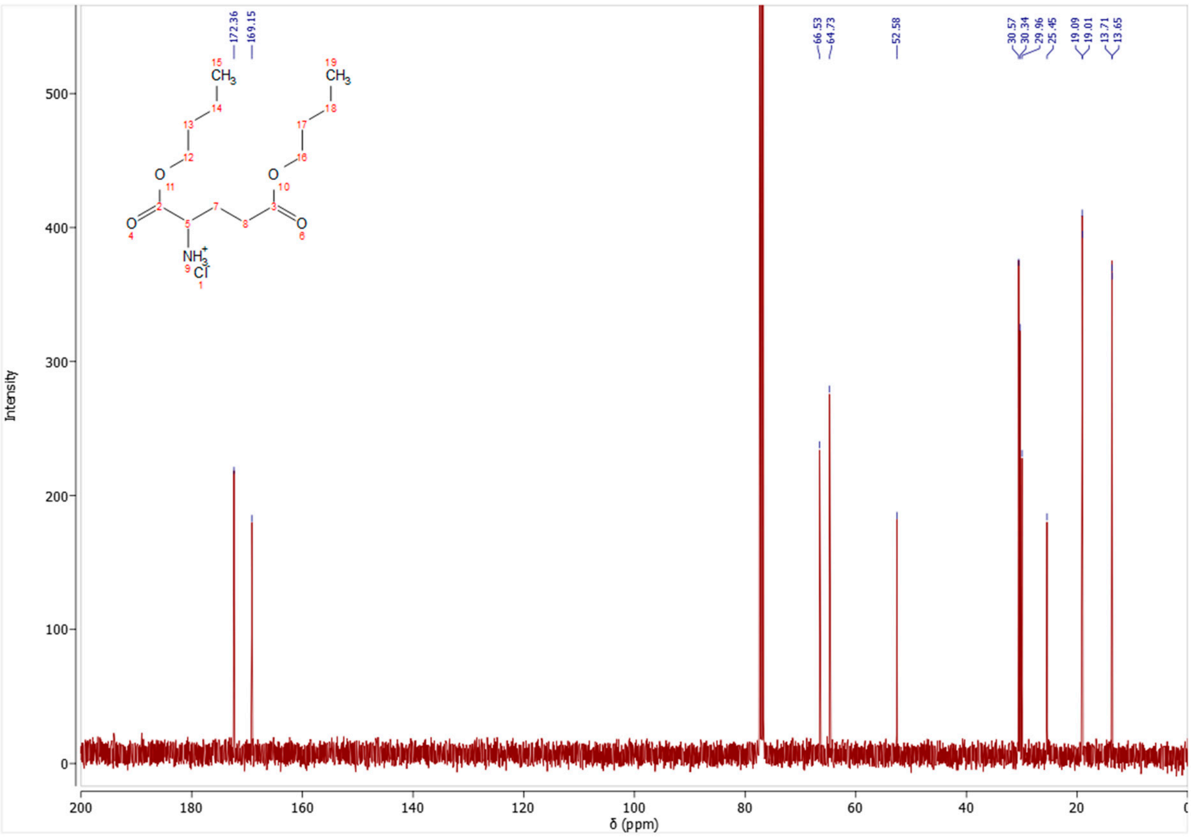

Figure S8. ^{13}C NMR spectrum of [Glu(OBu)$_2$][HCl].

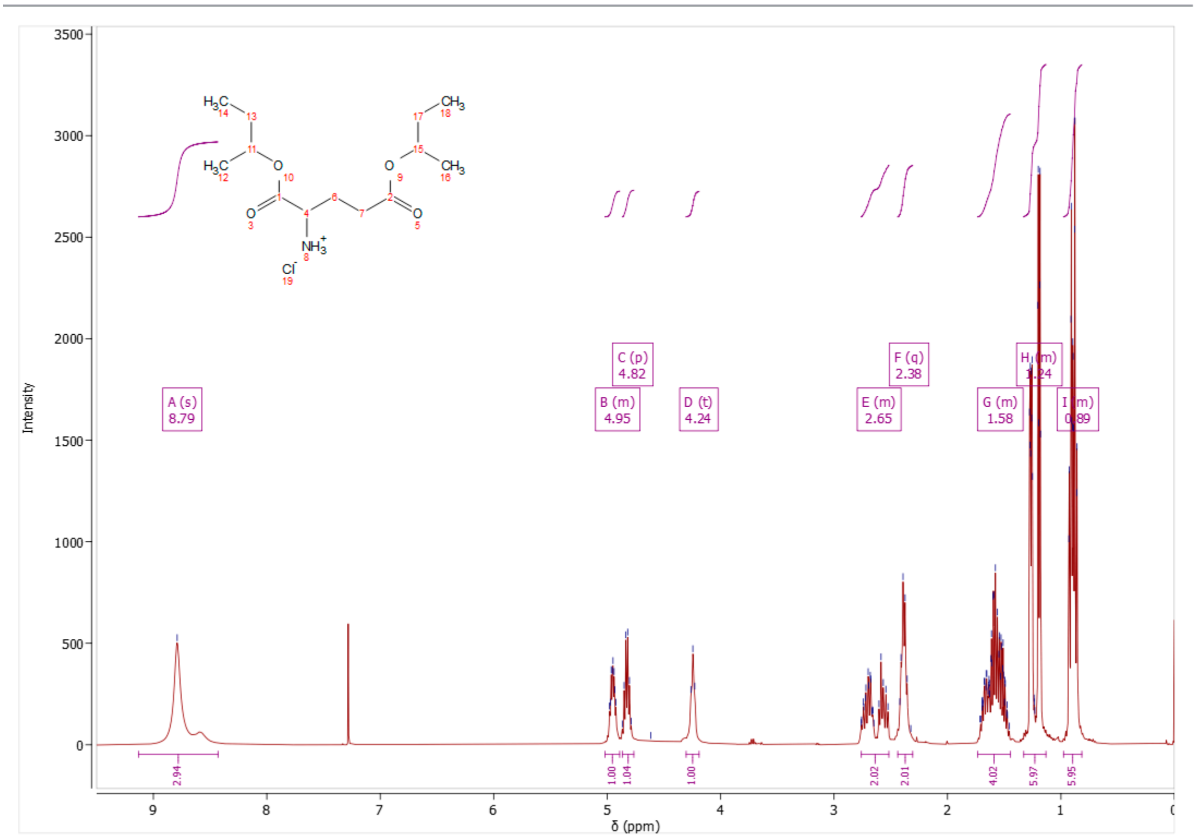

Figure S9. ^{1}H NMR spectrum of [Glu(Osec-Bu)$_2$][HCl].

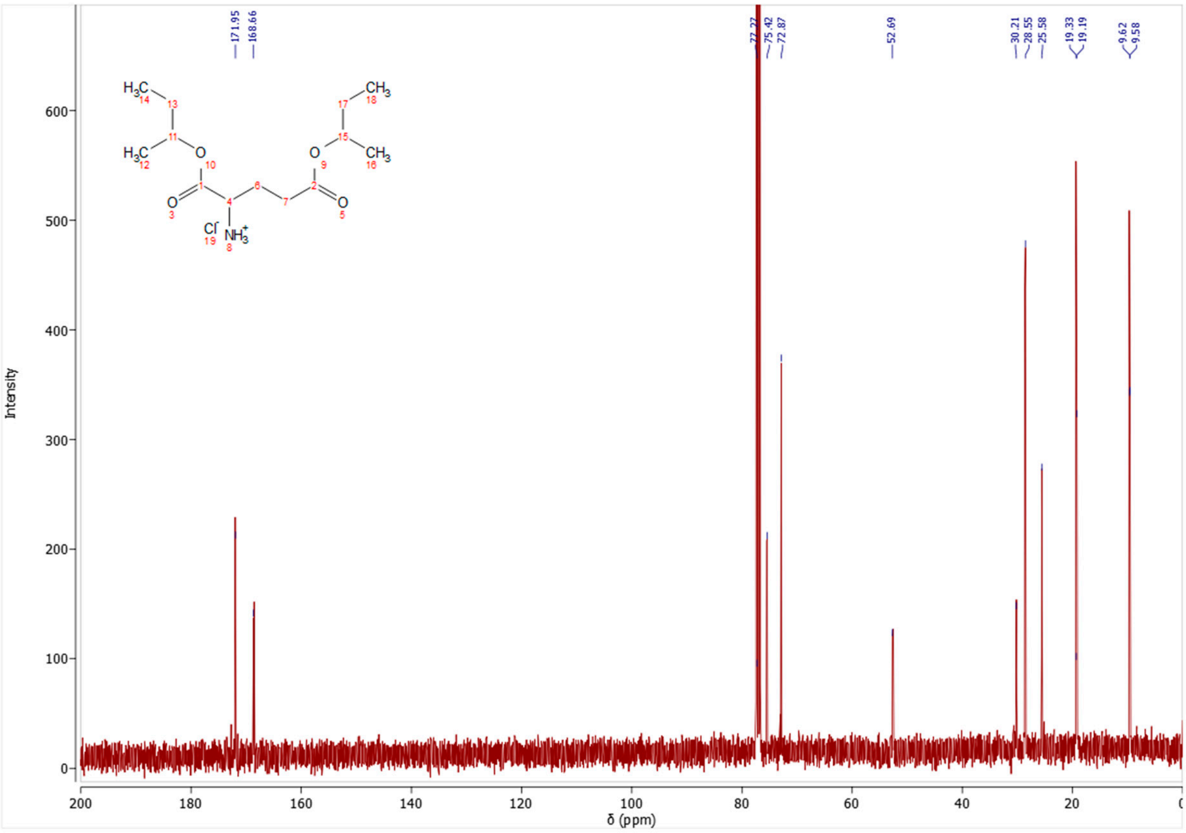

Figure S10. ^{13}C NMR spectrum of [Glu(Osec-Bu)$_2$][HCl].

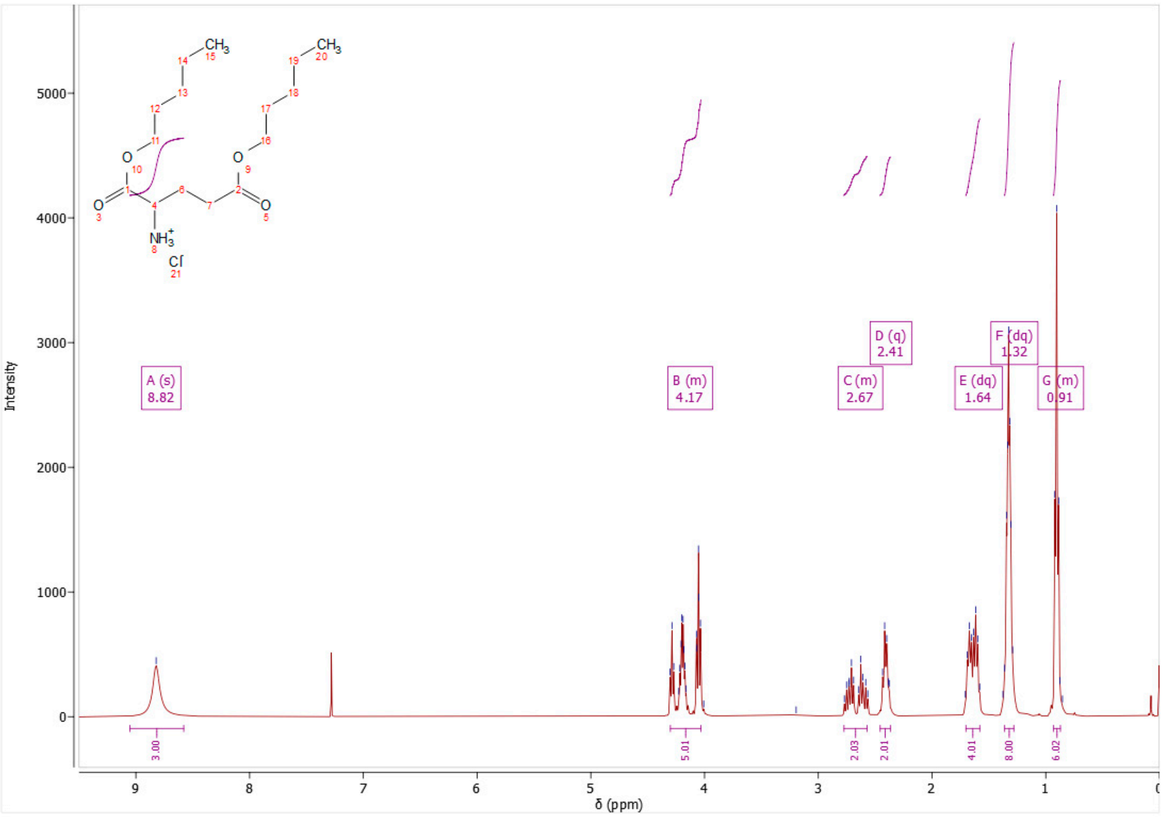

Figure S11. ^{1}H NMR spectrum of [Glu(OPent)$_2$][HCl].

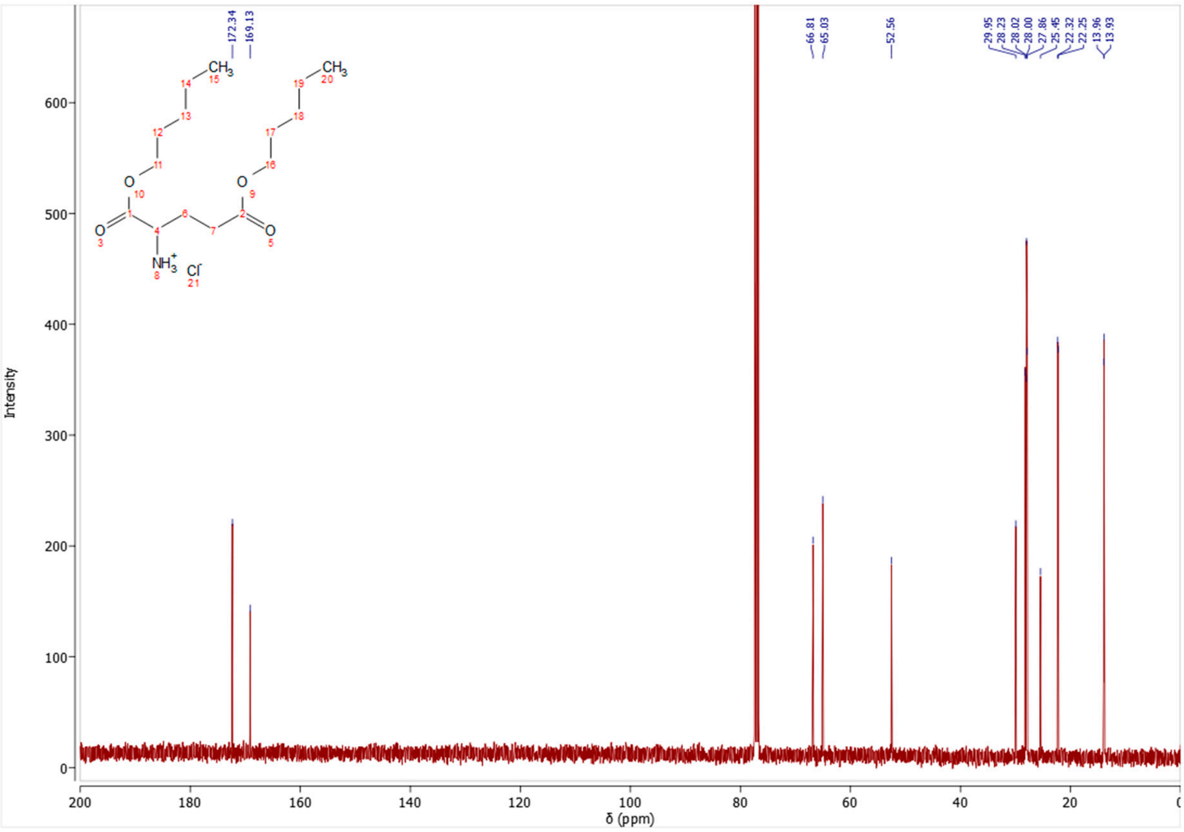

Figure S12. ^{13}C NMR spectrum of [Glu(OPent)$_2$][HCl].

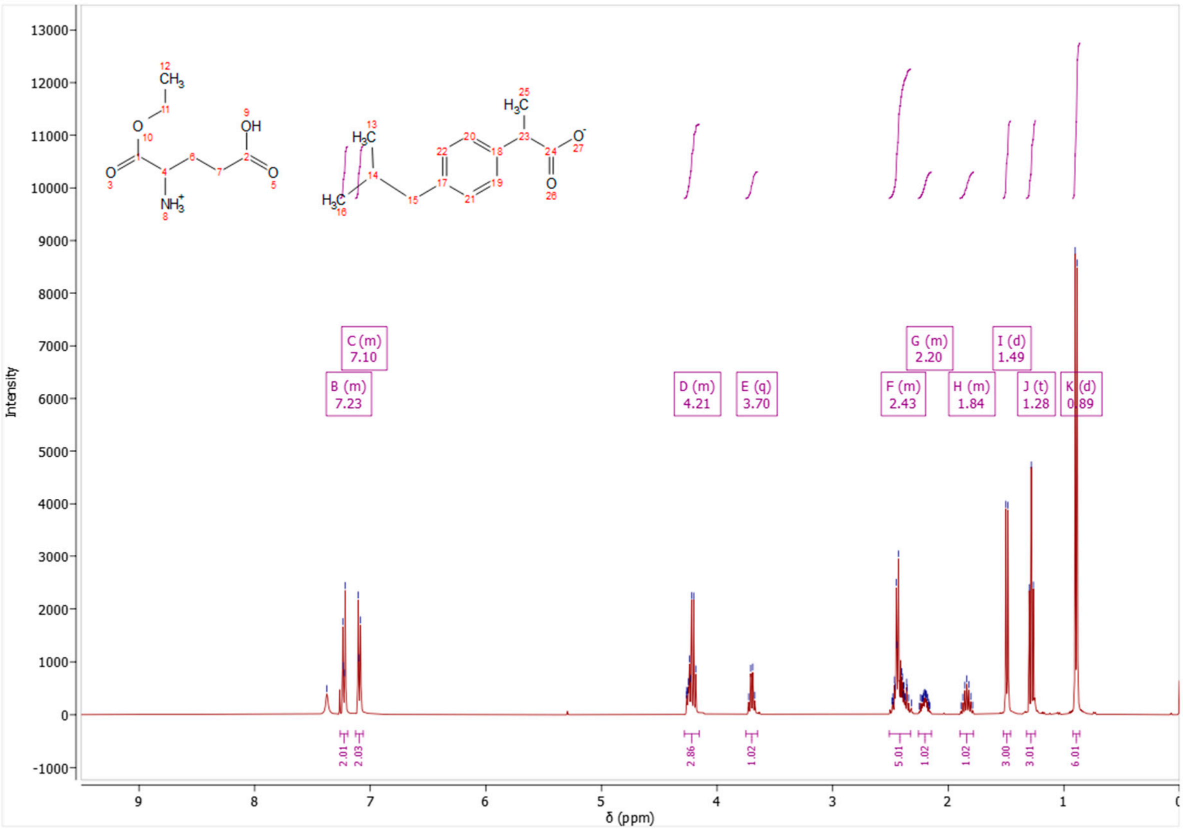

Figure S13. ^{1}H NMR spectrum of [Glu(OEt)][IBU].

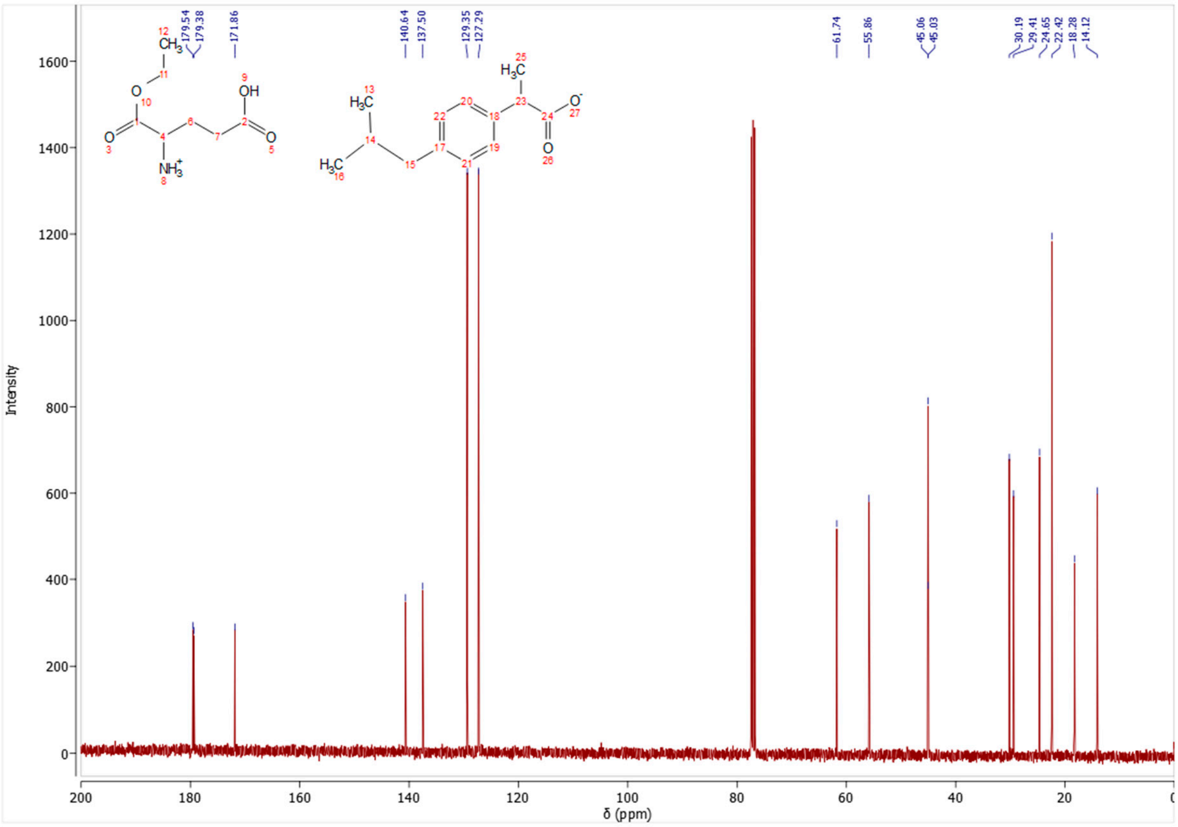

Figure S14. ^{13}C NMR spectrum of [Glu(OEt)][IBU].

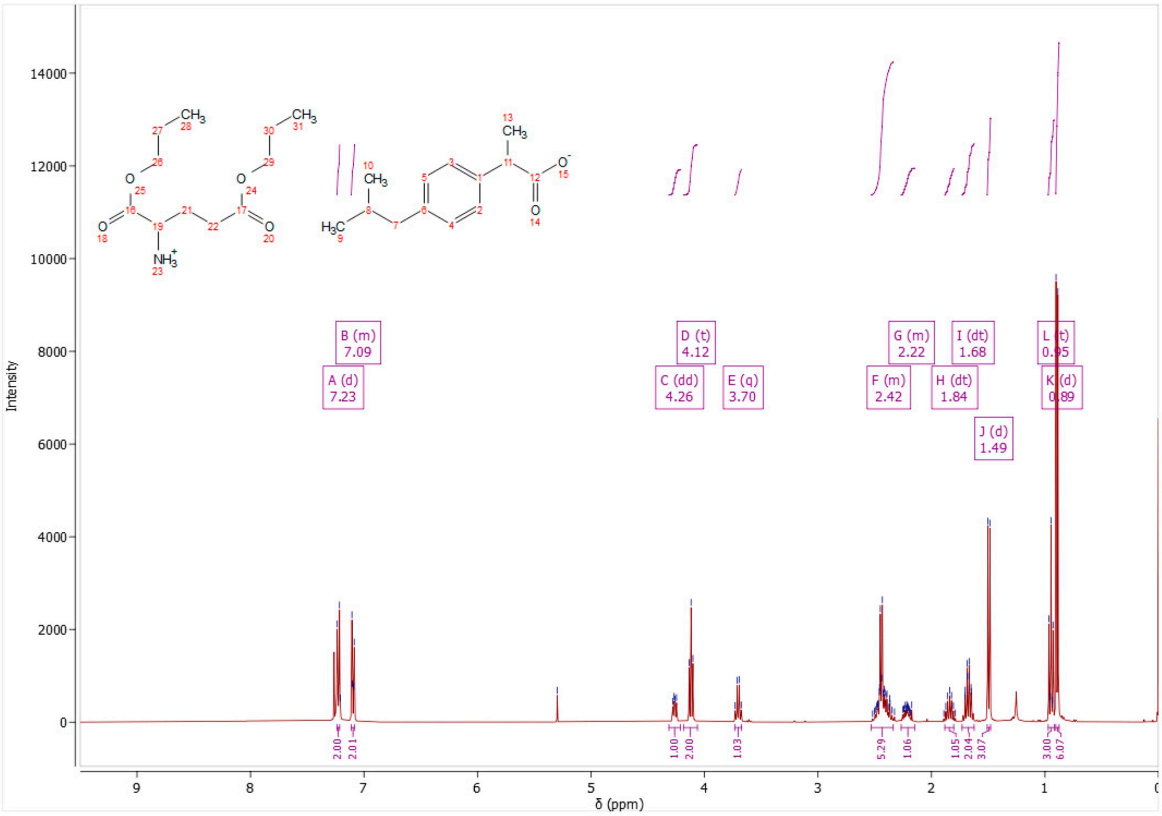

Figure S15. ^{1}H NMR spectrum of [Glu(OPr)][IBU].

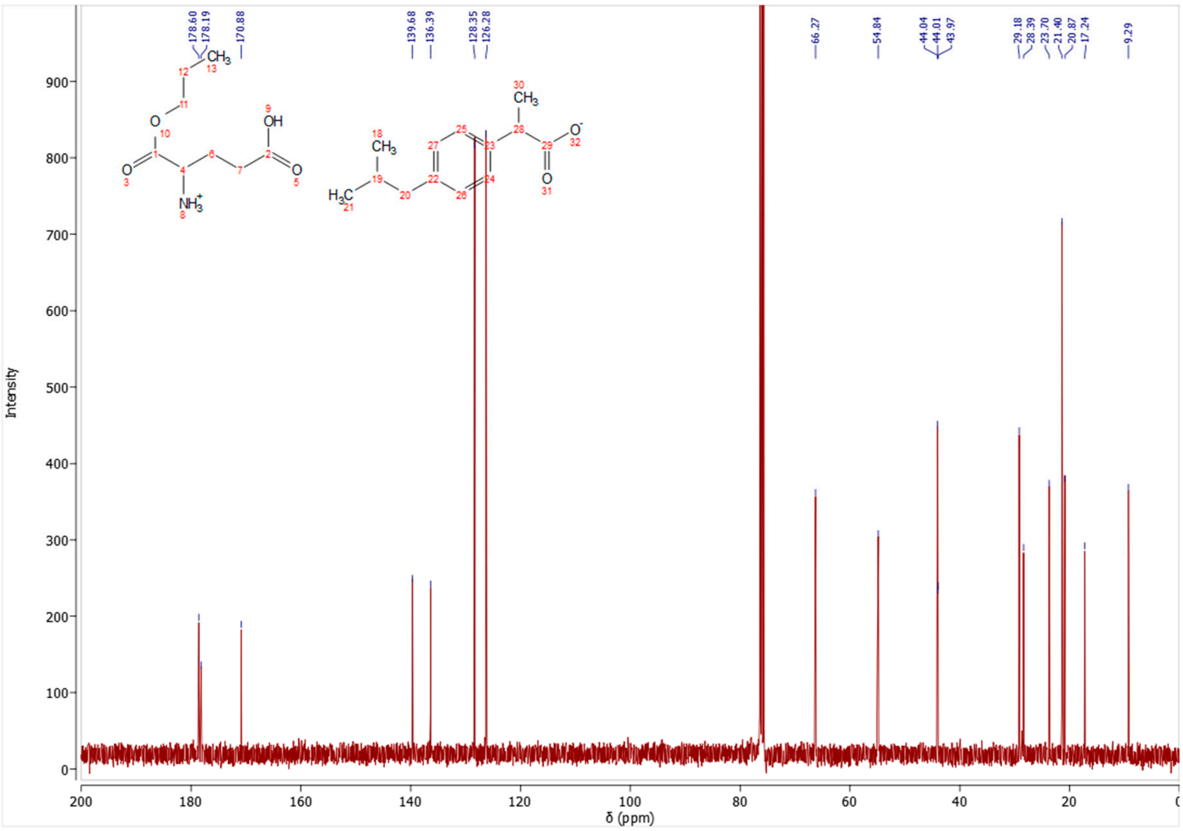

Figure S16. ^{13}C NMR spectrum of [Glu(OPr)][IBU].

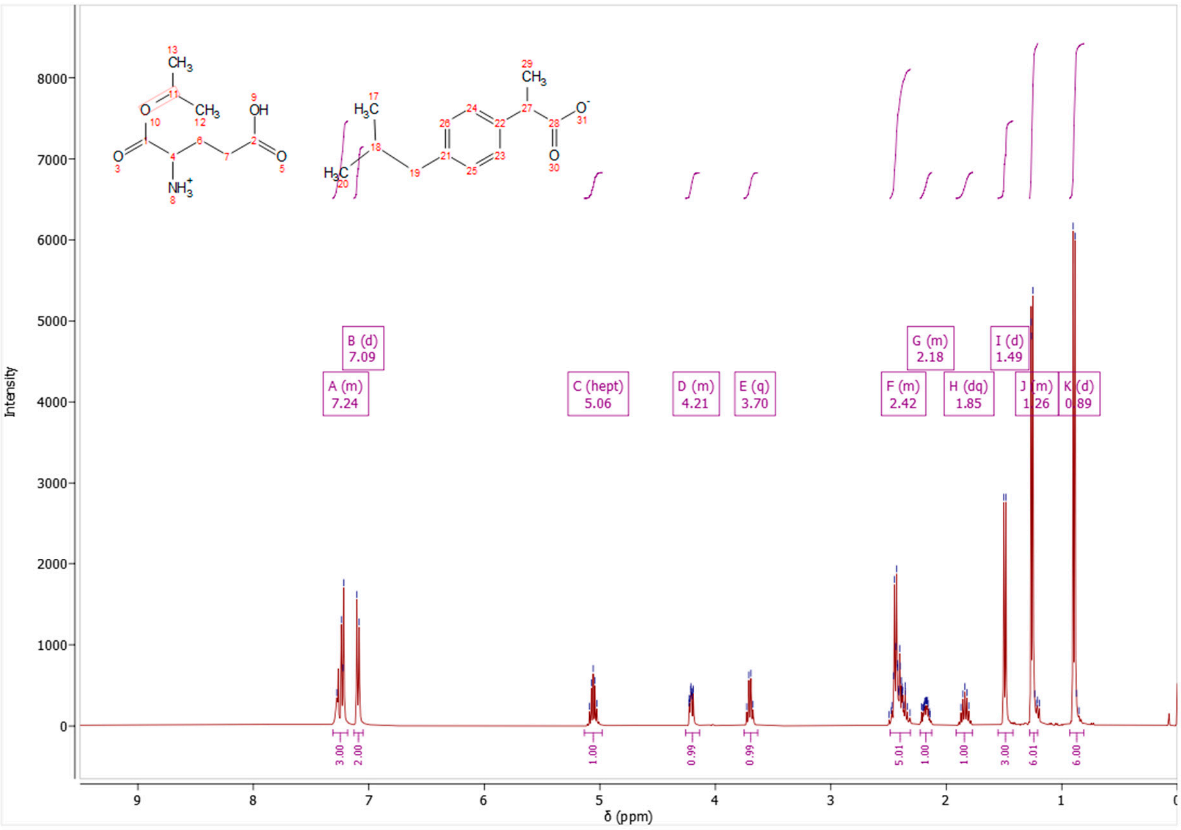

Figure S17. ^{1}H NMR spectrum of [Glu(OiPr)][IBU].

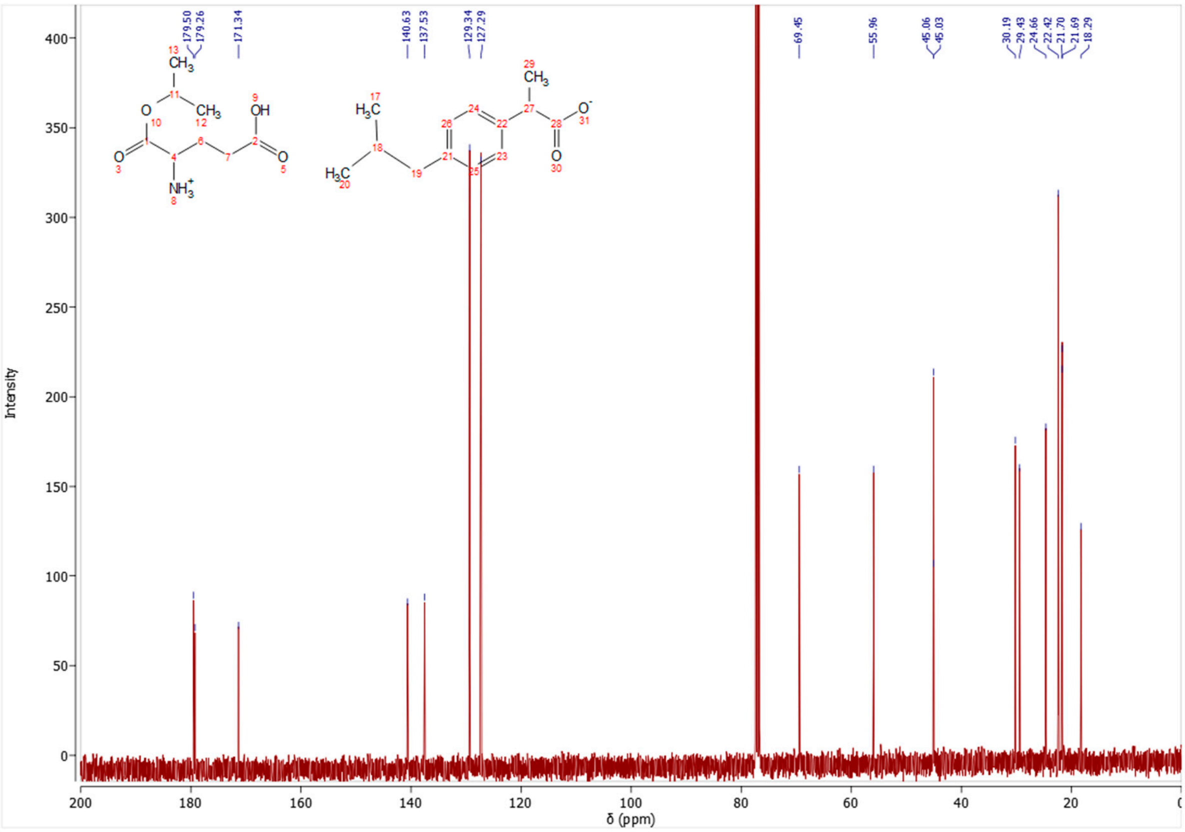

Figure S18. ^{13}C NMR spectrum of [Glu(OiPr)][IBU].

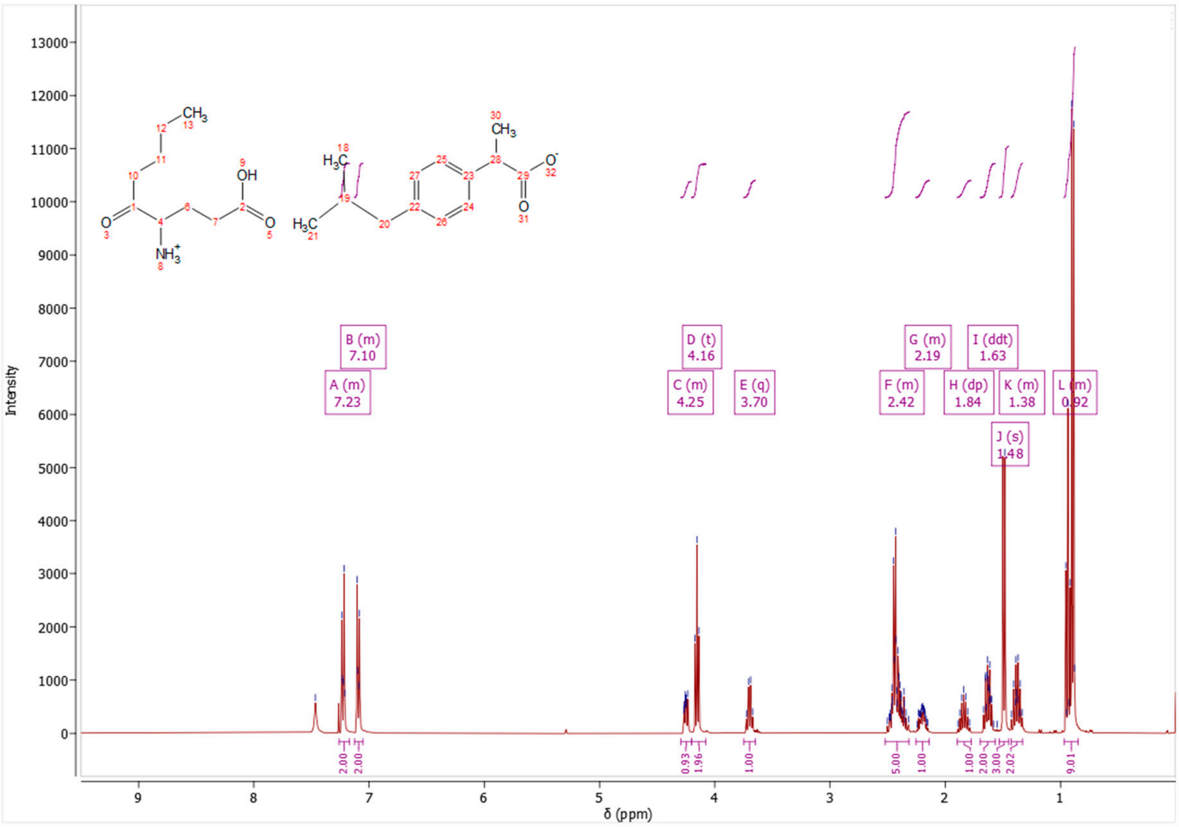

Figure S19. ¹H NMR spectrum of [Glu(OBu)][IBU].

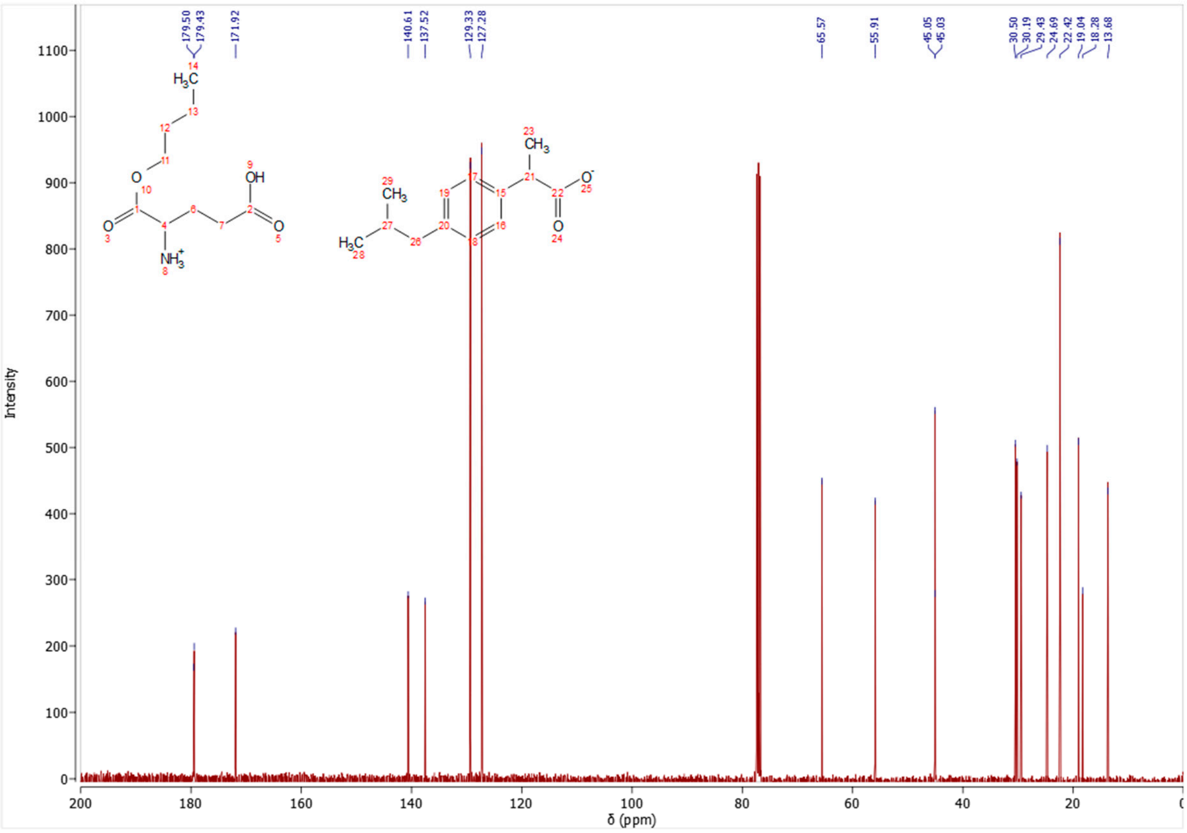

Figure S20. ¹³C NMR spectrum of [Glu(OBu)][IBU].

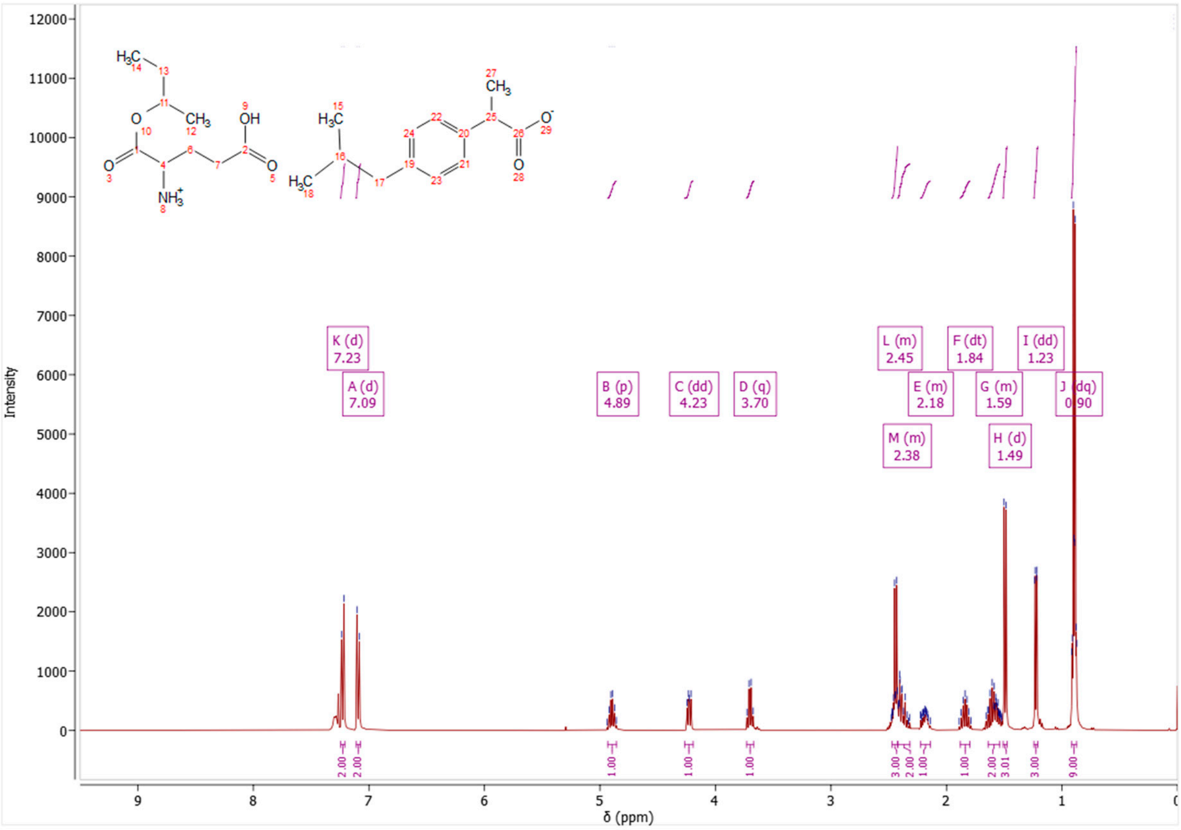

Figure S21. ^{1}H NMR spectrum of [Glu(Osec-Bu)][IBU].

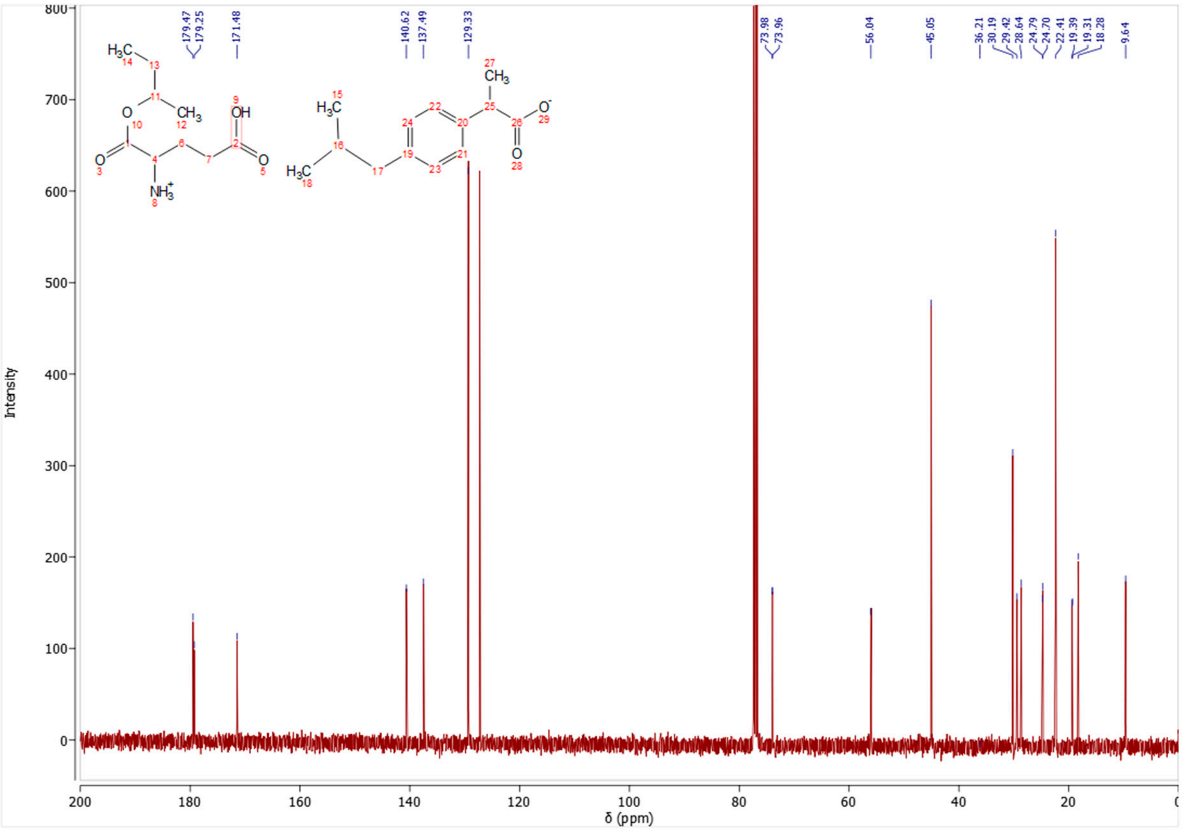

Figure S22. ^{13}C NMR spectrum of [Glu(Osec-Bu)][IBU].

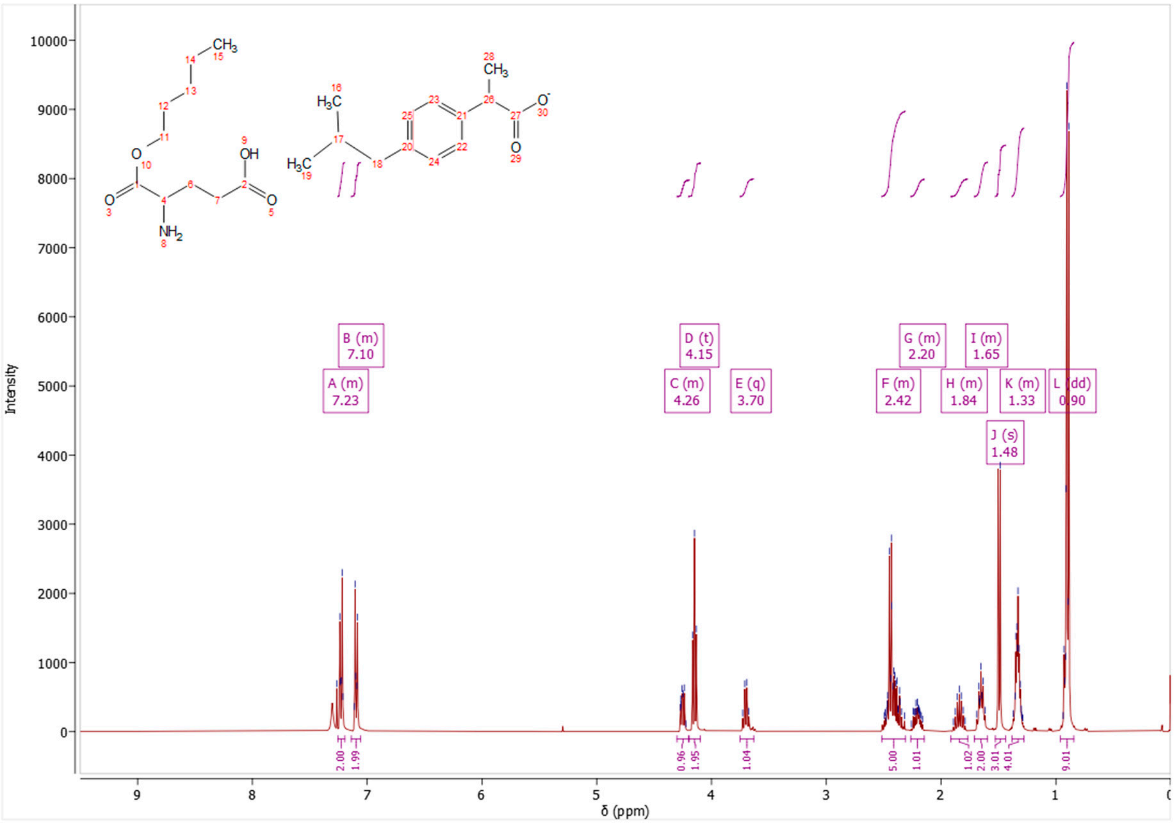

Figure S23. ^{1}H NMR spectrum of [Glu(OPent)][IBU].

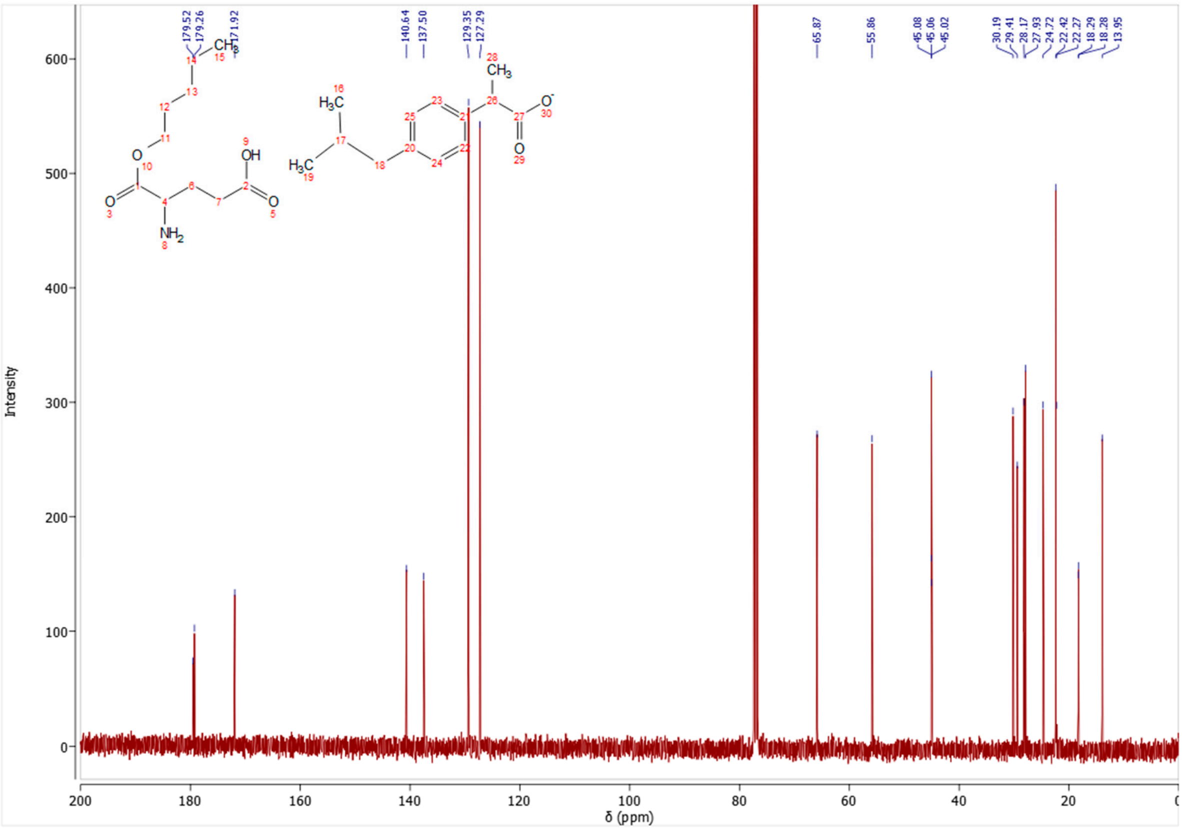

Figure S24. ^{13}C NMR spectrum of [Glu(OPent)][IBU].

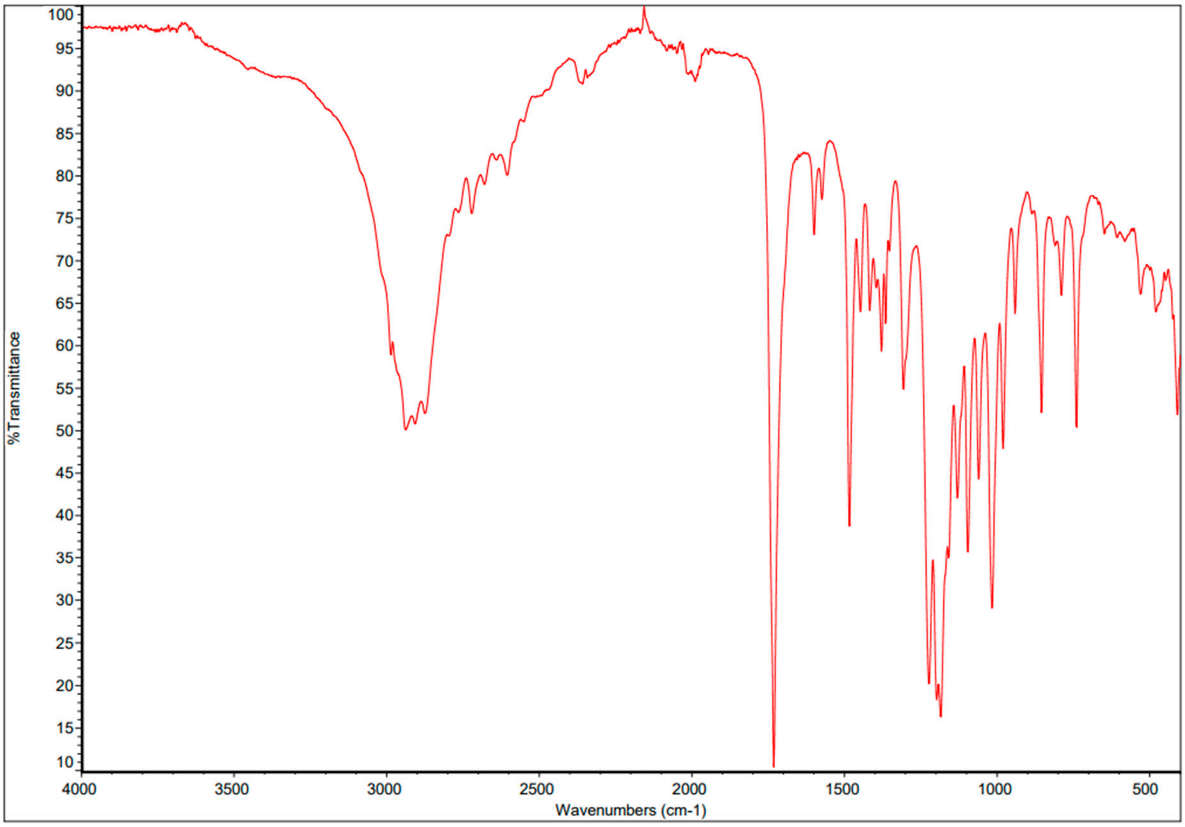

Figure S25. FTIR spectrum of [Glu(OEt)$_2$][HCl].

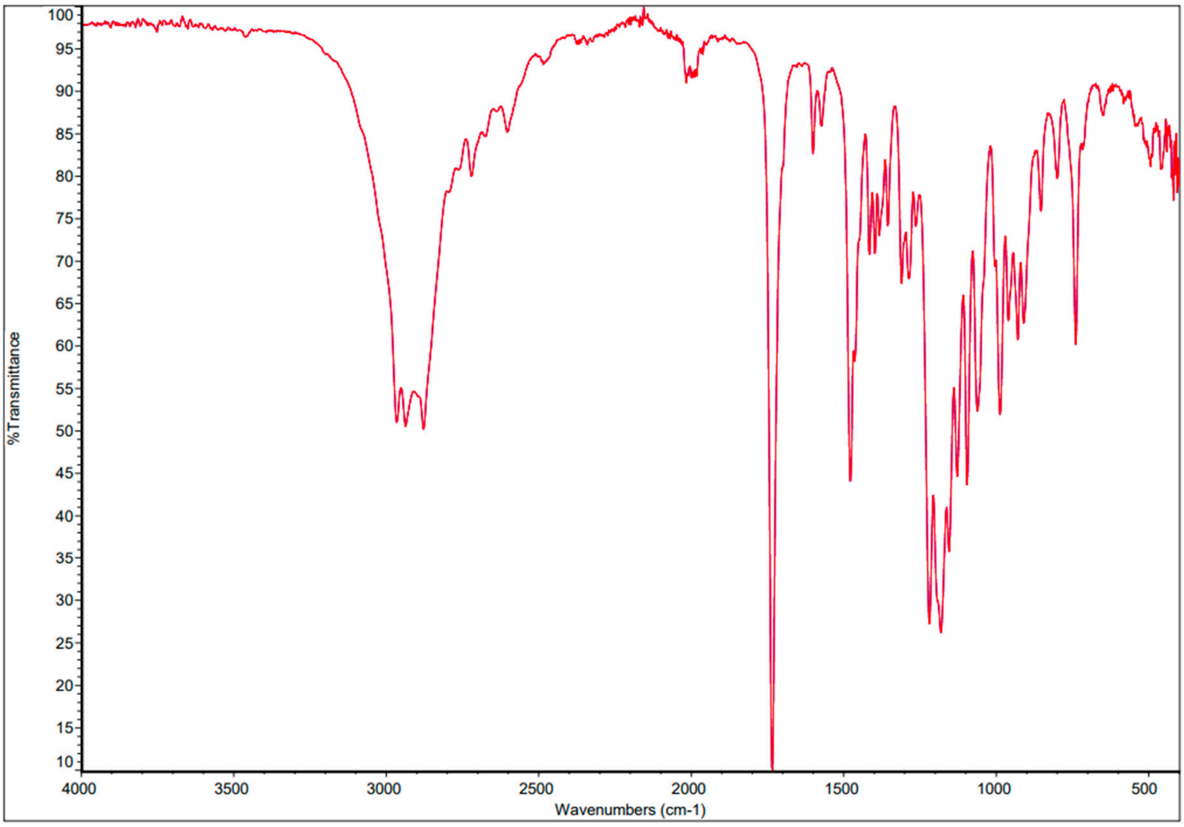

Figure S26. FTIR spectrum of [Glu(OPr)$_2$][HCl].

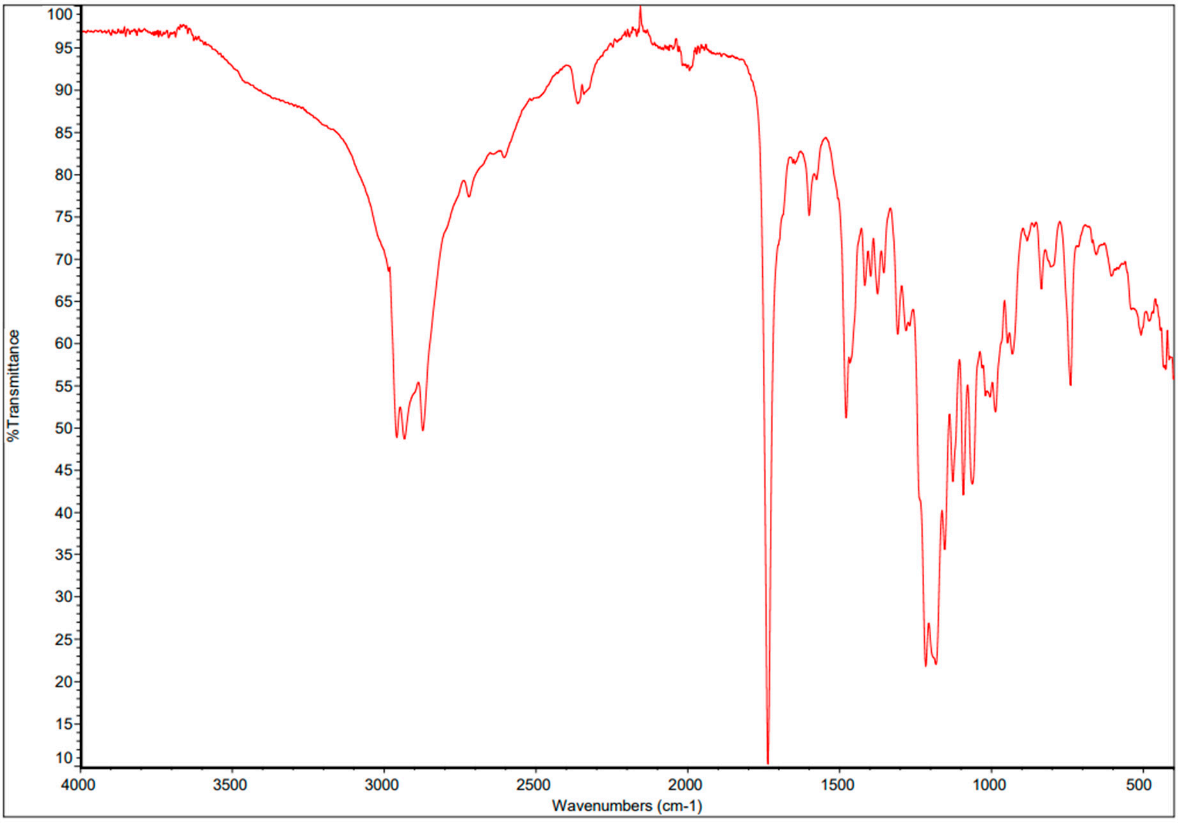

Figure S27. FTIR spectrum of [Glu(OBu)$_2$][HCl].

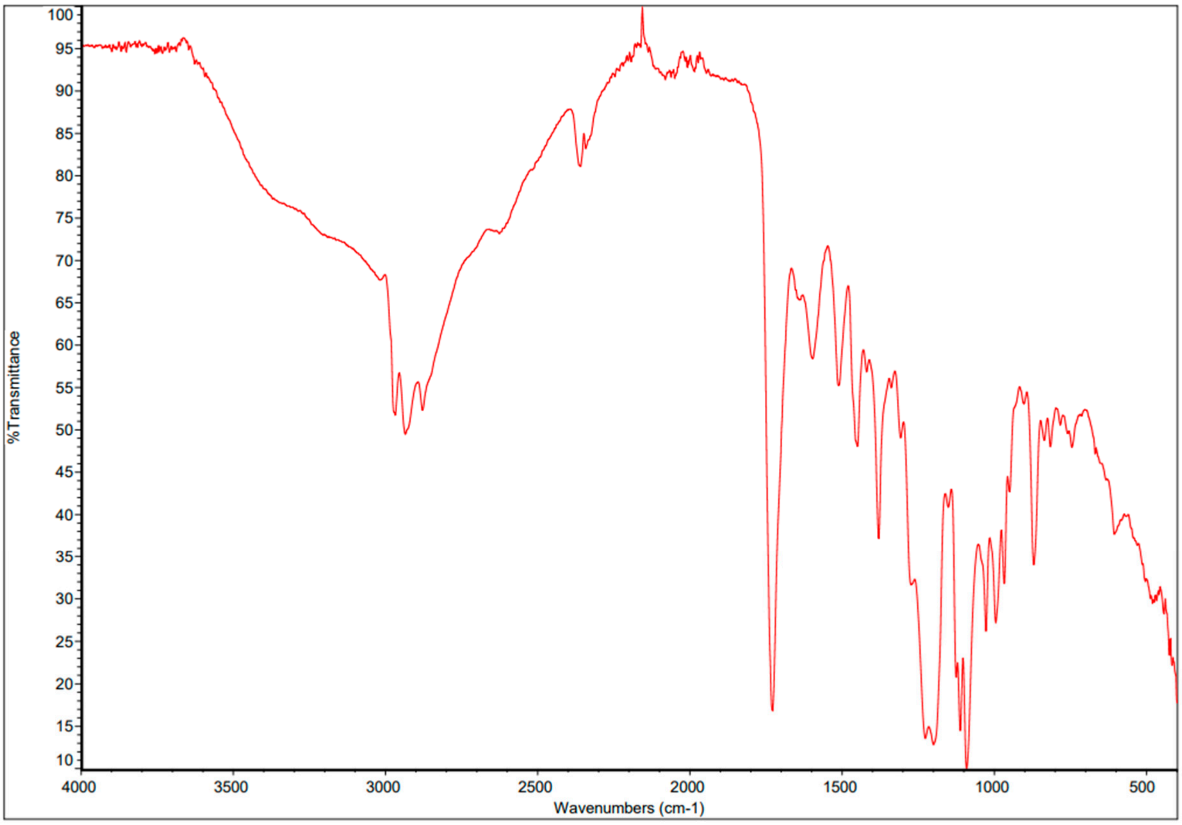

Figure S28. FTIR spectrum of [Glu(Osec-Bu)$_2$][HCl].

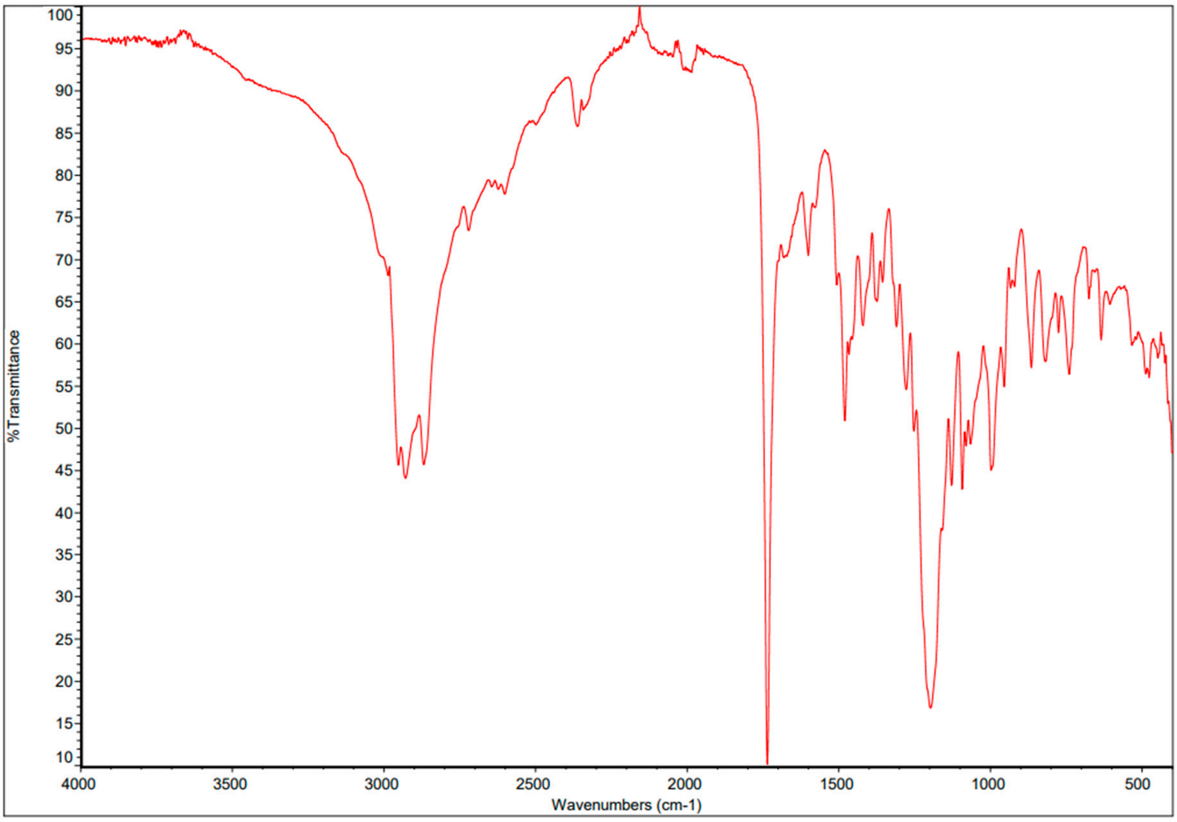

Figure S29. FTIR spectrum of [Glu(OPent)$_2$][HCl].

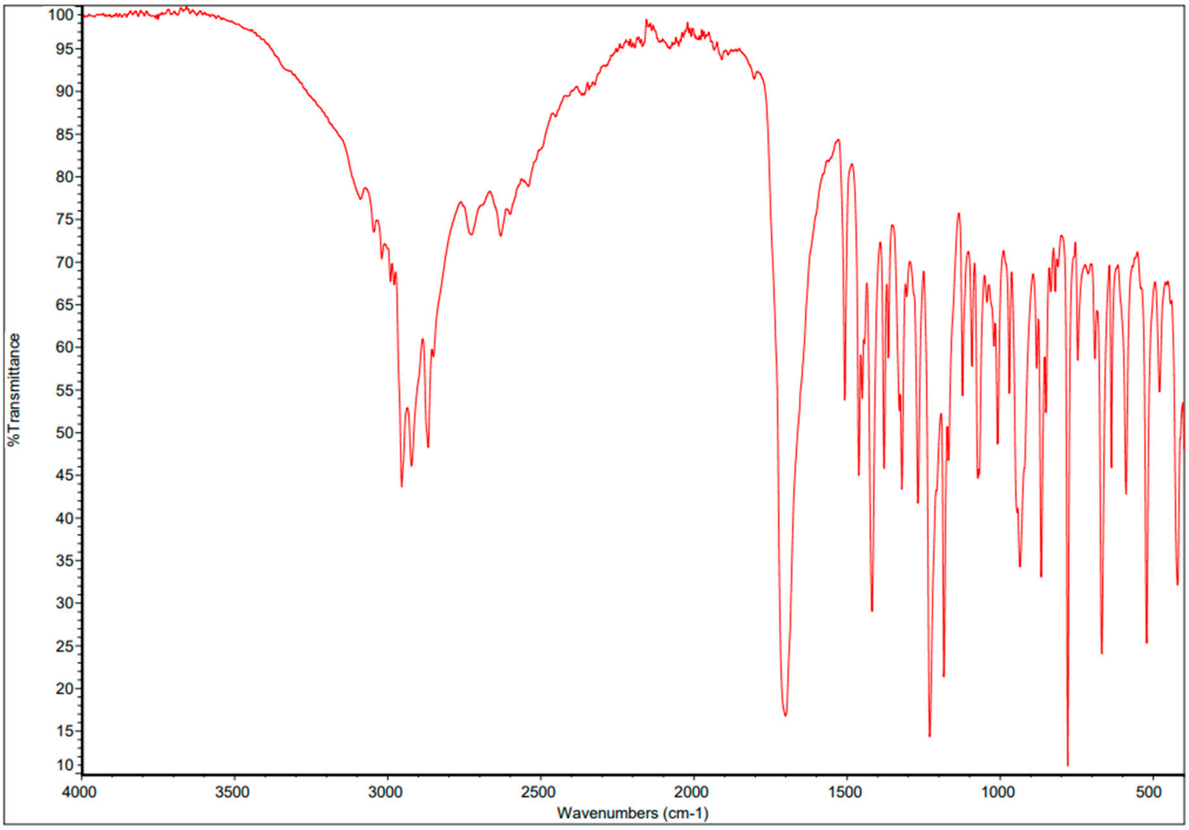

Figure S30. FTIR spectrum of [Glu(OEt)][IBU].

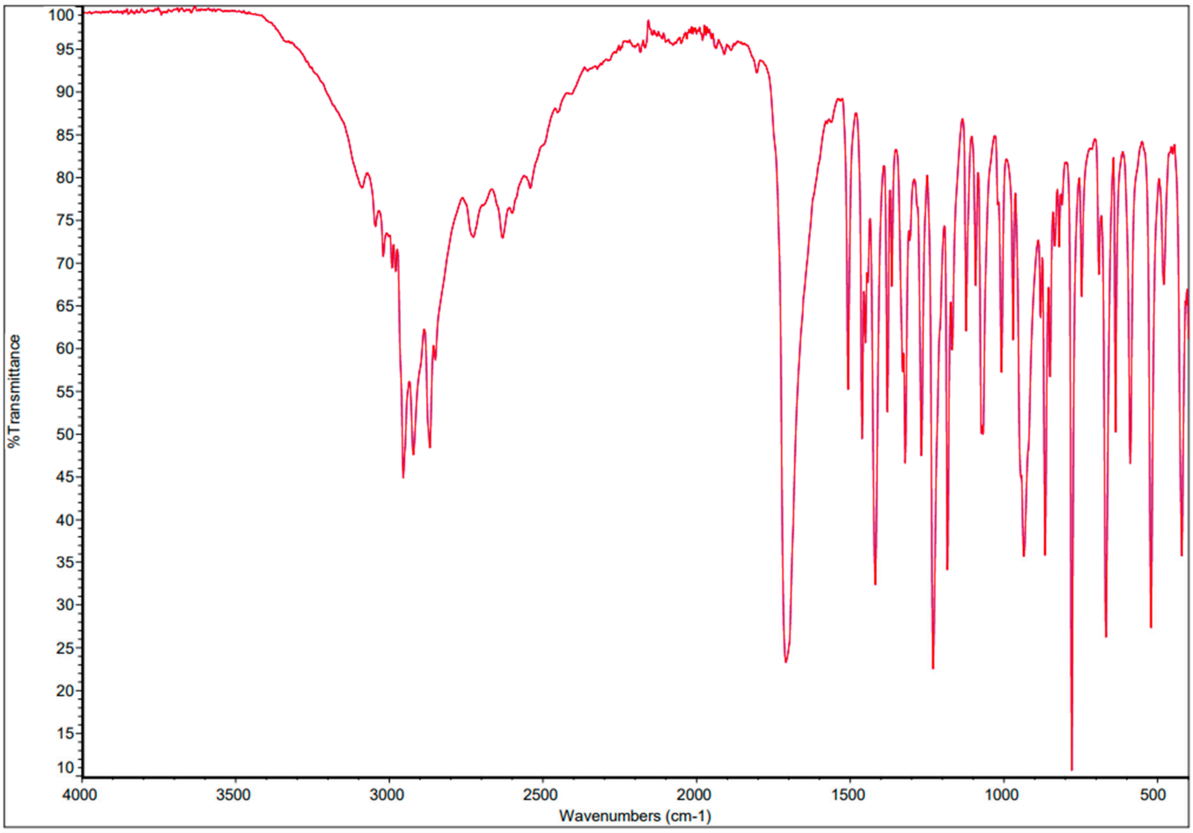

Figure S31. FTIR spectrum of [Glu(OPr)][IBU].

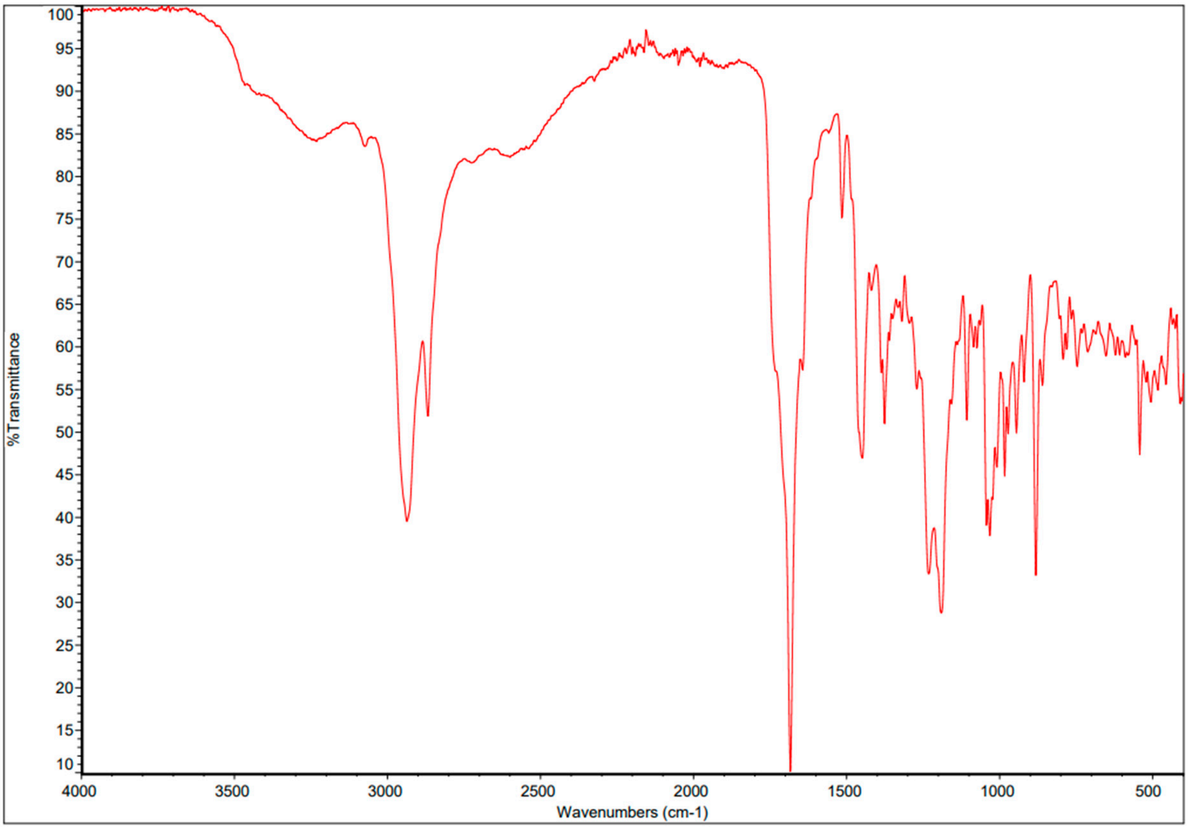

Figure S32. FTIR spectrum of [Glu(OBu)][IBU].

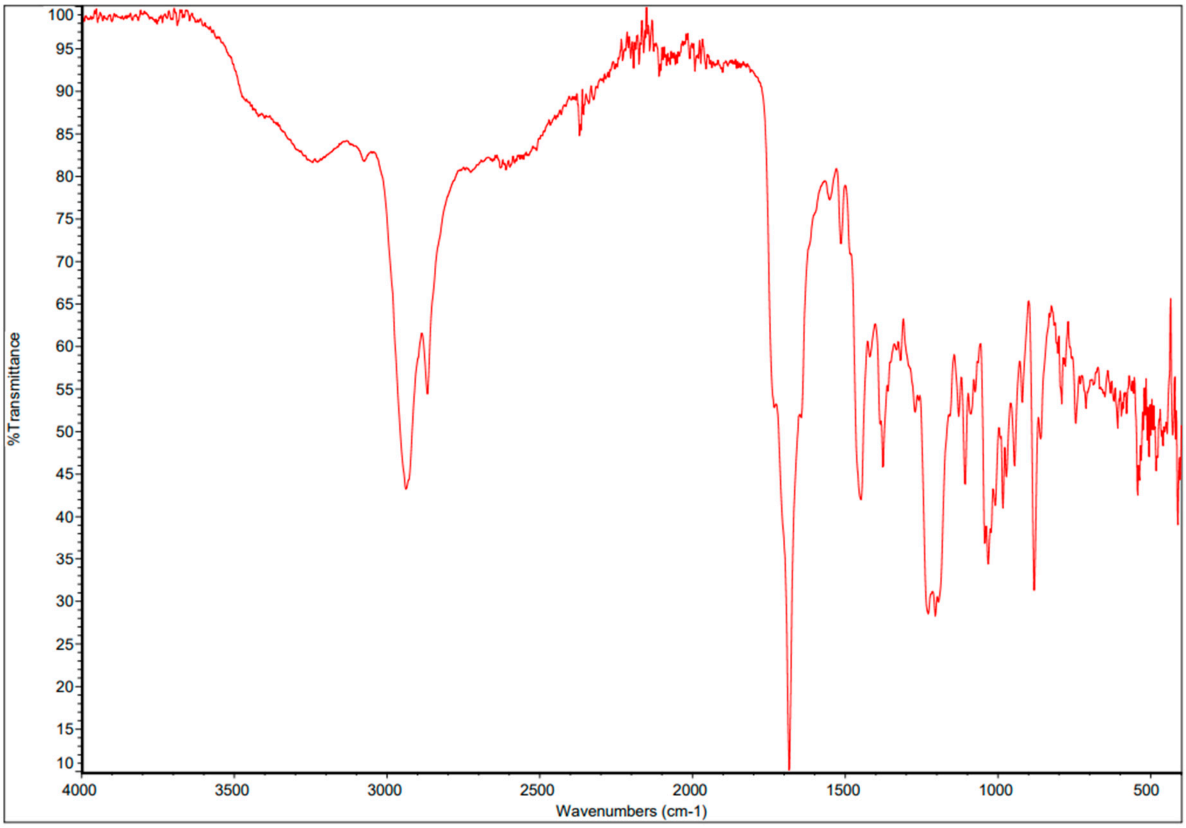

Figure S33. FTIR spectrum of [Glu(Osec-Bu)][IBU].

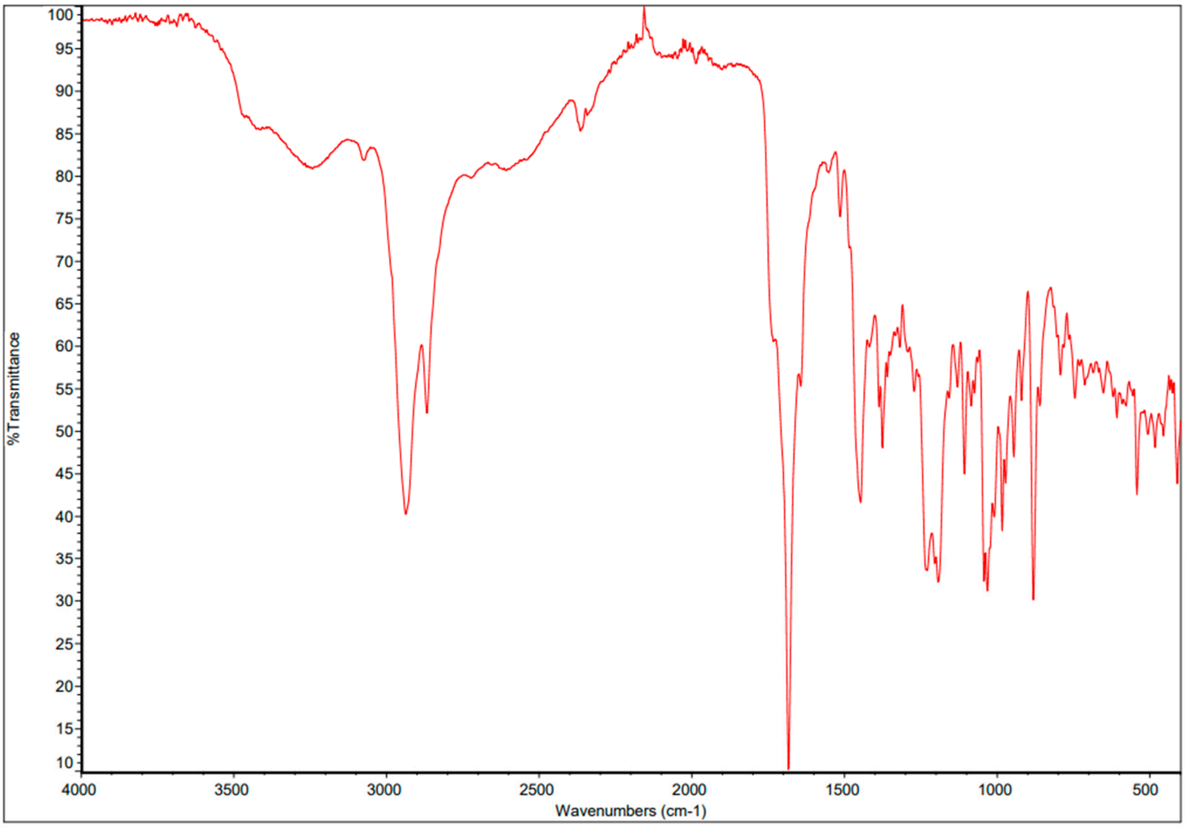

Figure S34. FTIR spectrum of [Glu(OPent)][IBU].

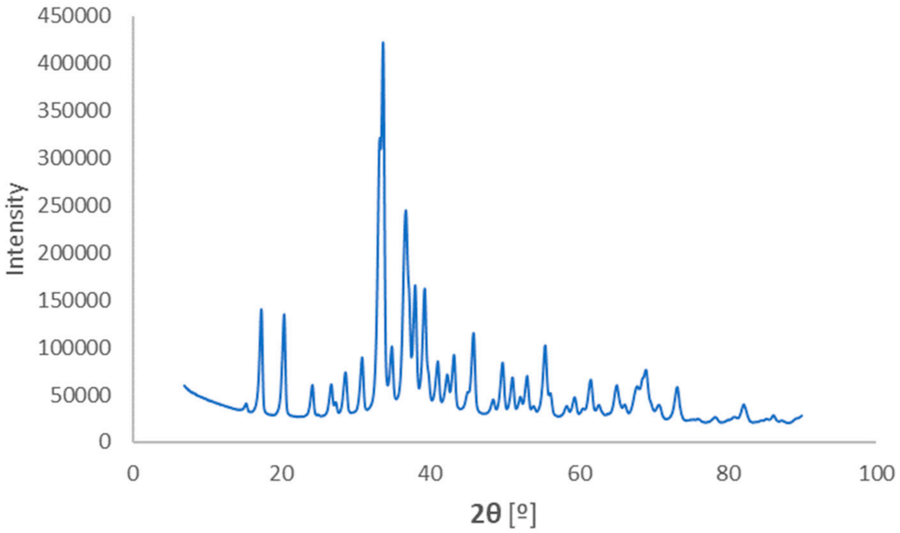

Figure S35. XRD pattern of [Glu(OEt)$_2$][HCl].

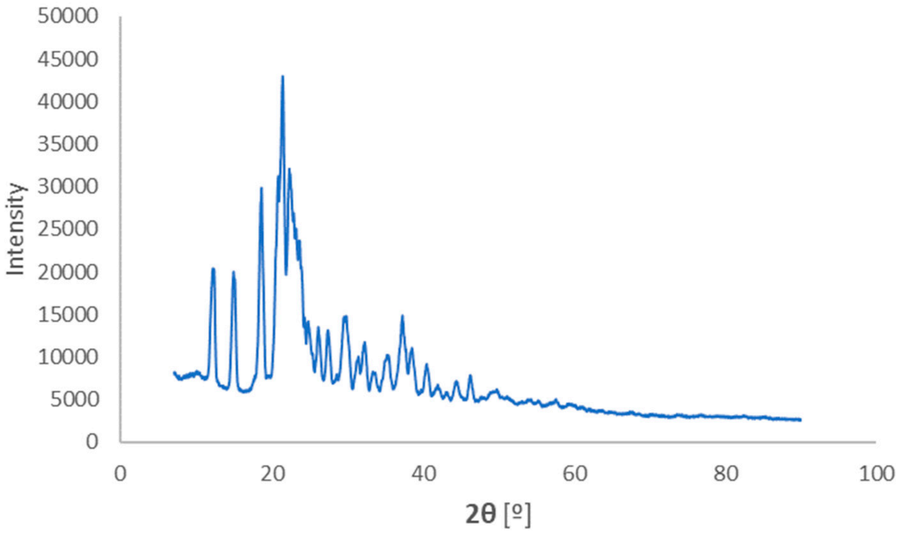

Figure S36. XRD pattern of [Glu(OPr)$_2$][HCl].

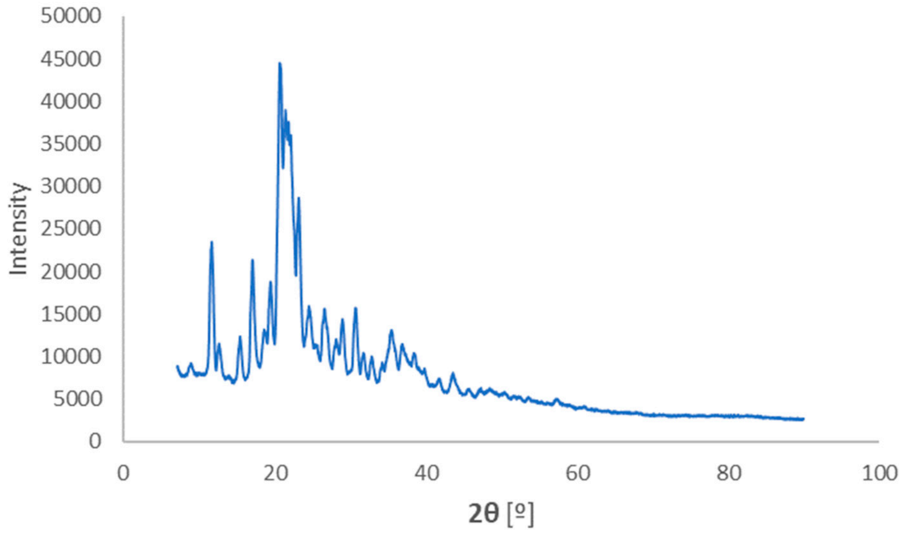

Figure S37. XRD pattern of [Glu(OBu)$_2$][HCl].

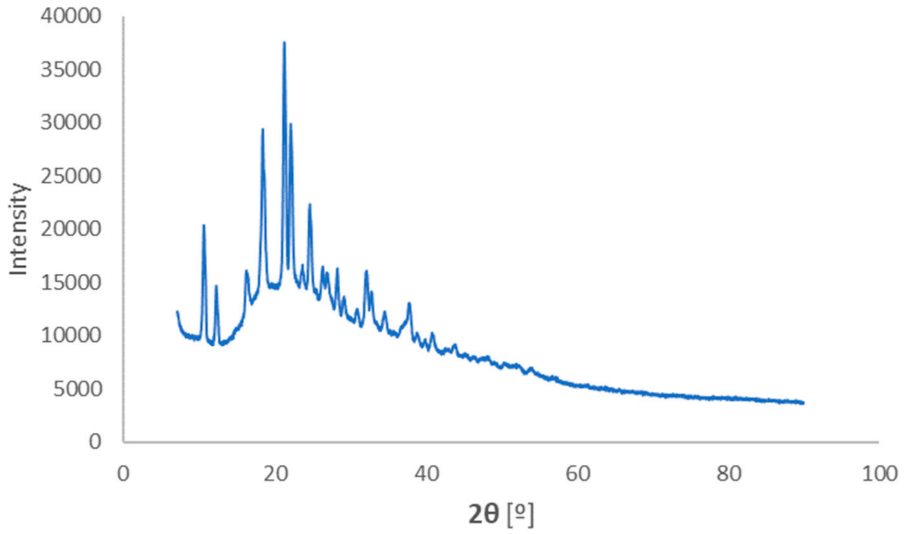

Figure S38. XRD pattern of [Glu(Osec-Bu)$_2$][HCl].

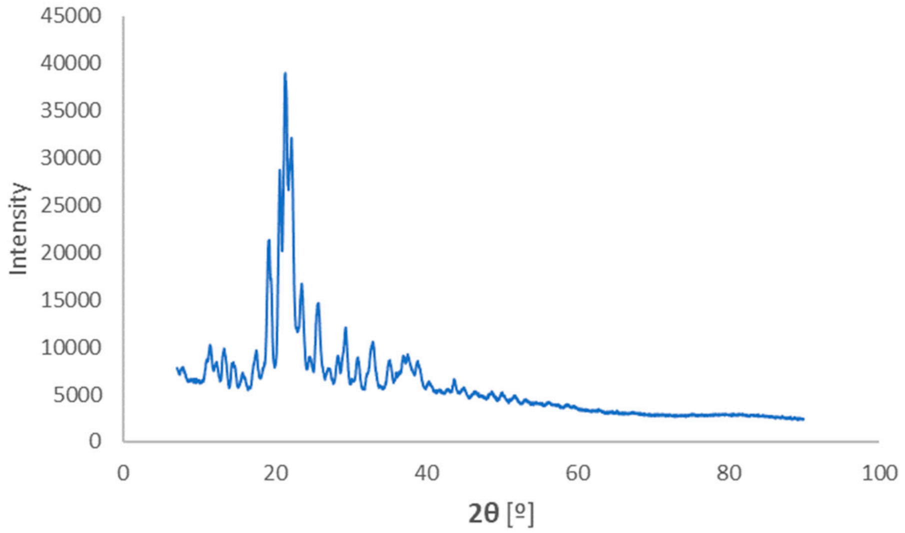

Figure S39. XRD pattern of [Glu(OPent)$_2$][HCl].

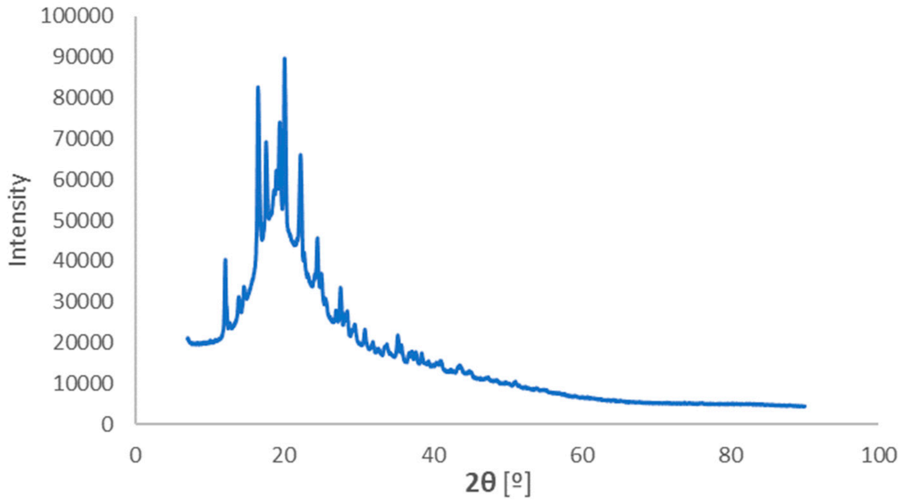

Figure S40. XRD pattern of [Glu(OEt)][IBU].

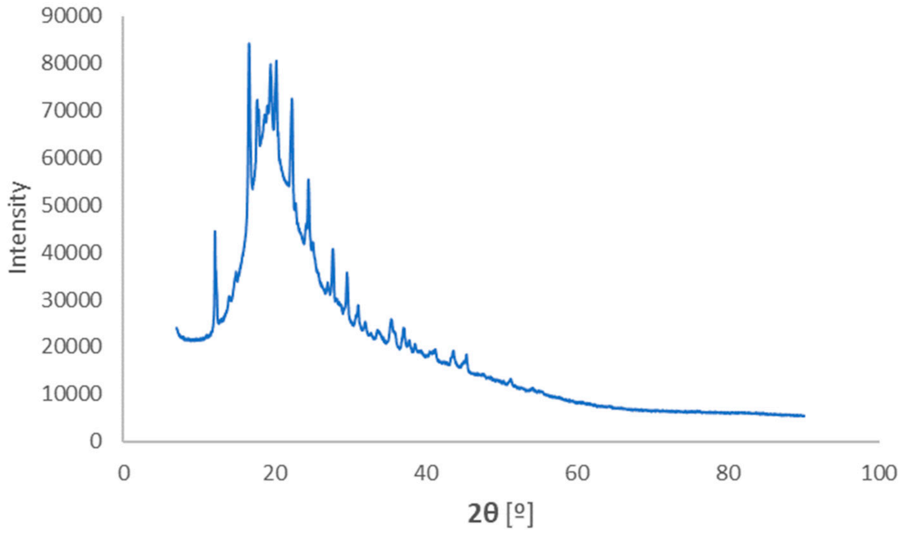

Figure S41. XRD pattern of [Glu(OPr)][IBU].

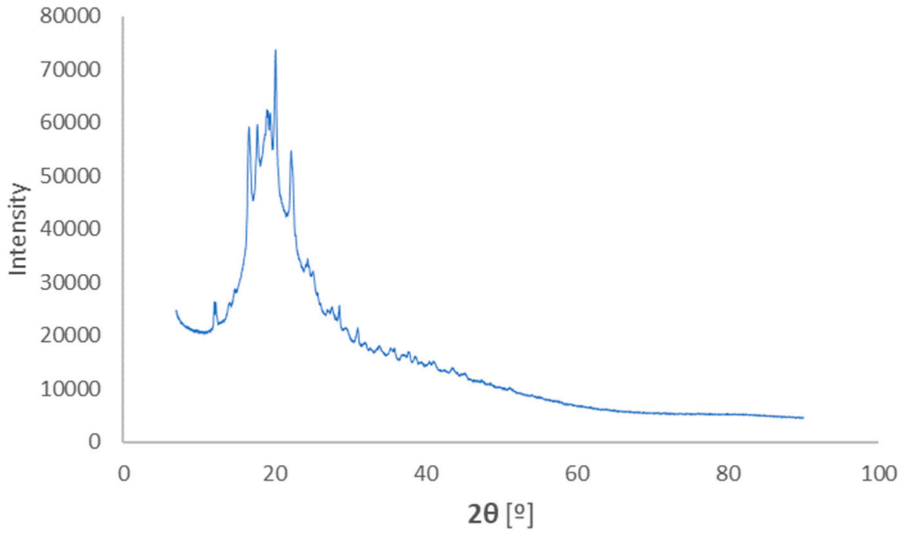

Figure S42. XRD pattern of [Glu(OBu)][IBU].

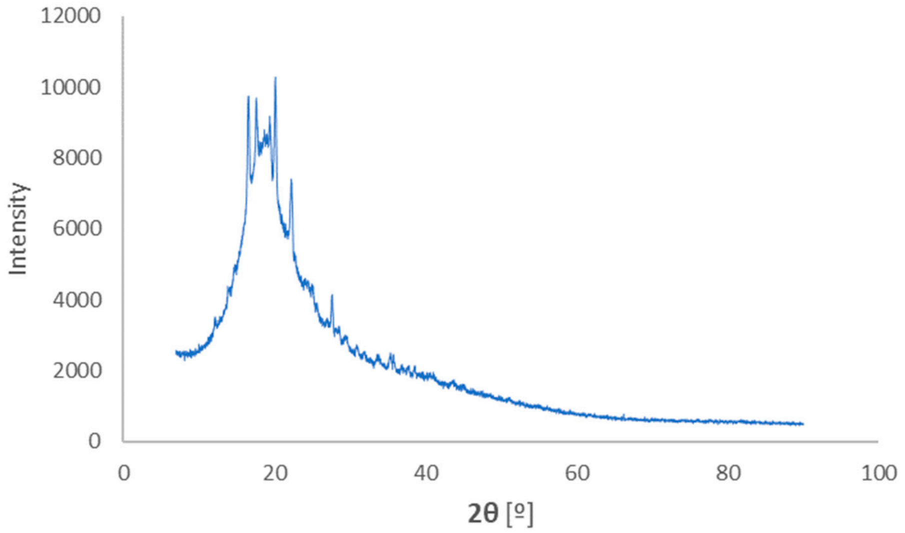

Figure S43. XRD pattern of [Glu(Osec-Bu)][IBU].

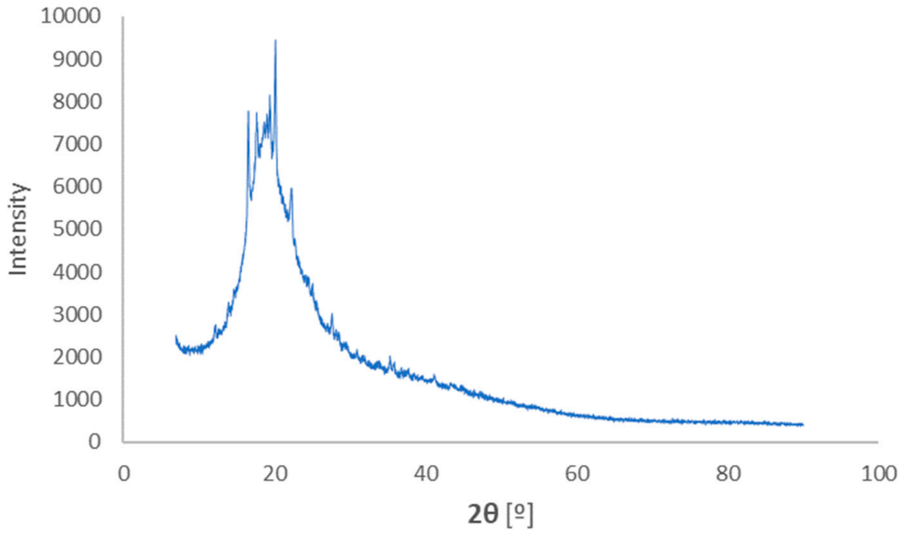

Figure S44. XRD pattern of [Glu(OPent)][IBU].

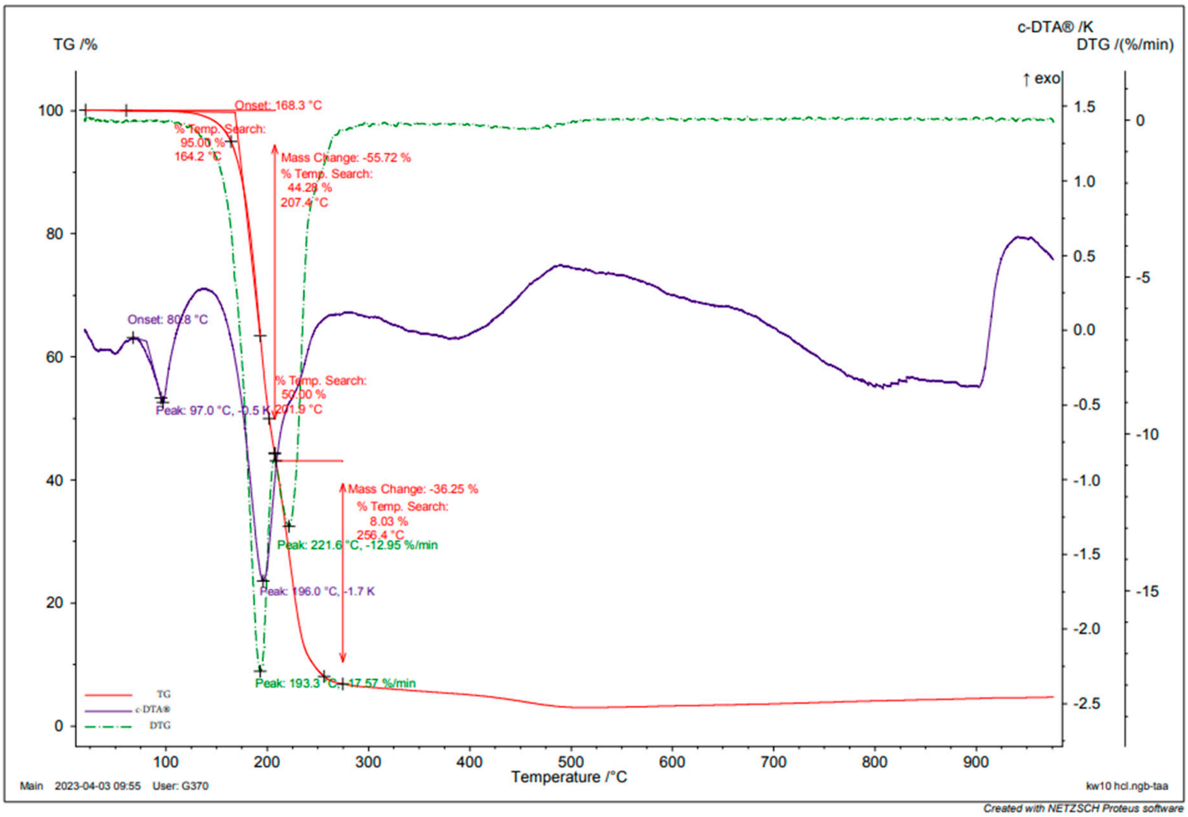

Figure S45. TG, DTG, c-DTA curves of [Glu(OEt)2][HCl].

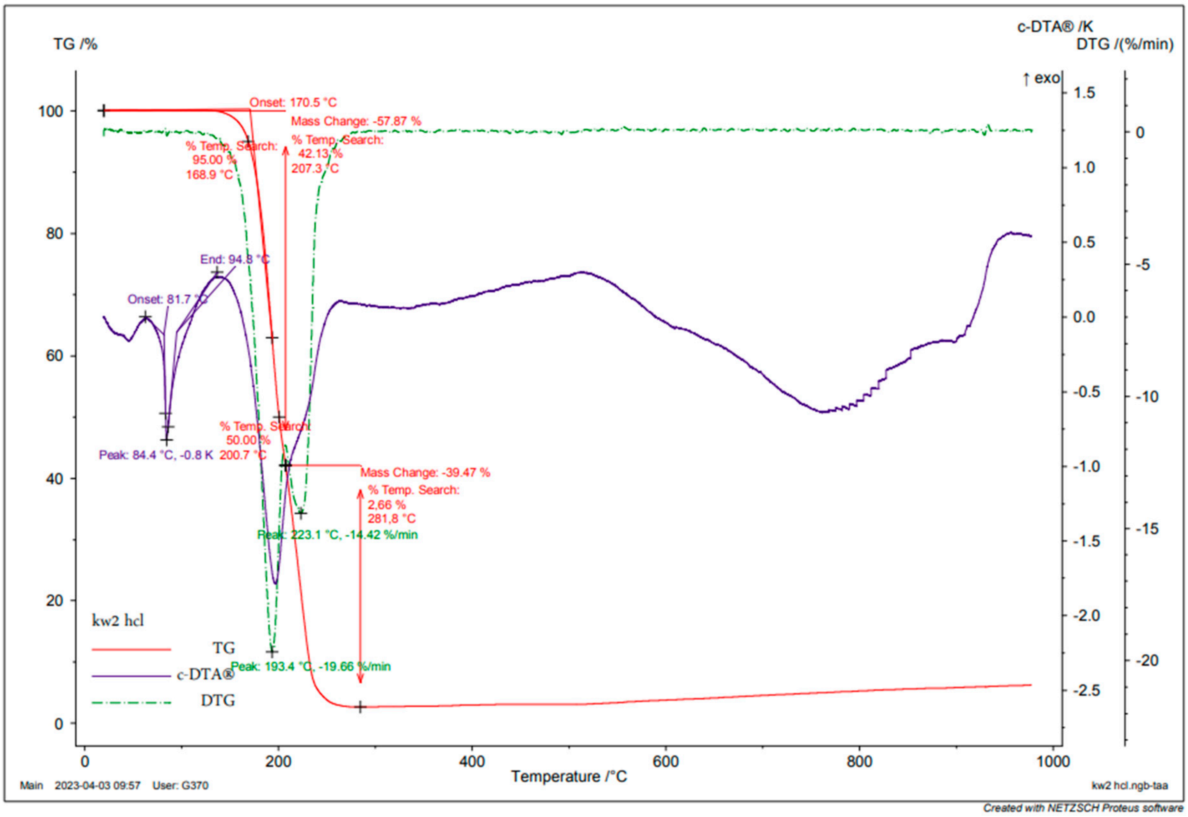

Figure S46. TG, DTG, c-DTA curves of [Glu(OPr)2][HCl].

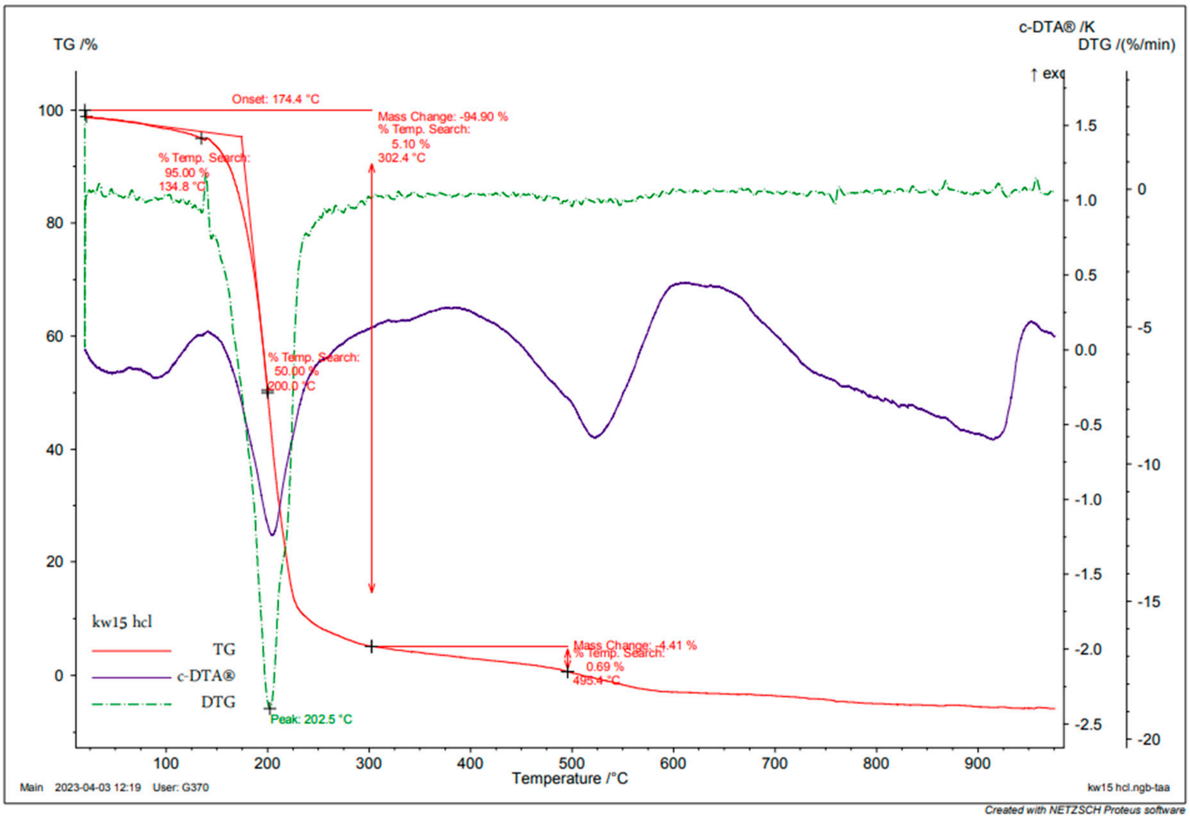

Figure S47. TG, DTG, c-DTA curves of [Glu(OiPr)2][HCl].

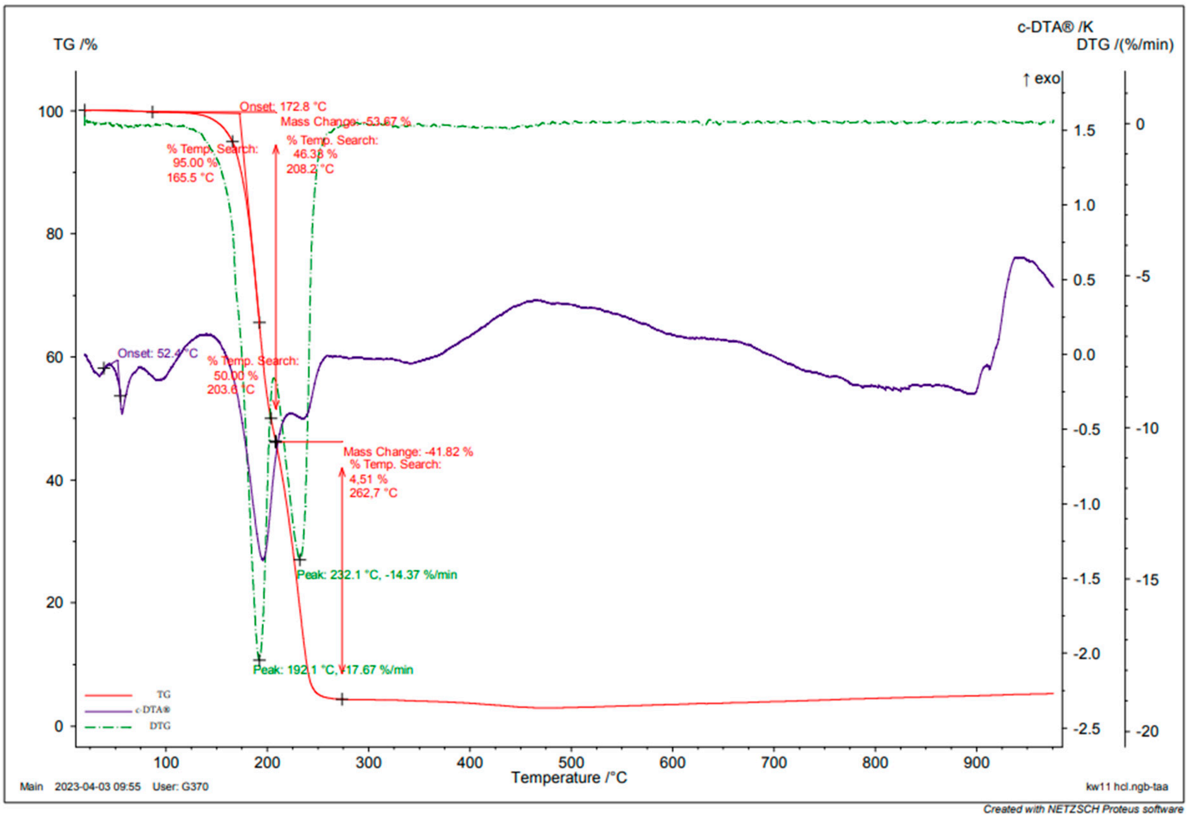

Figure S48. TG, DTG, c-DTA curves of [Glu(OBu)2][HCl].

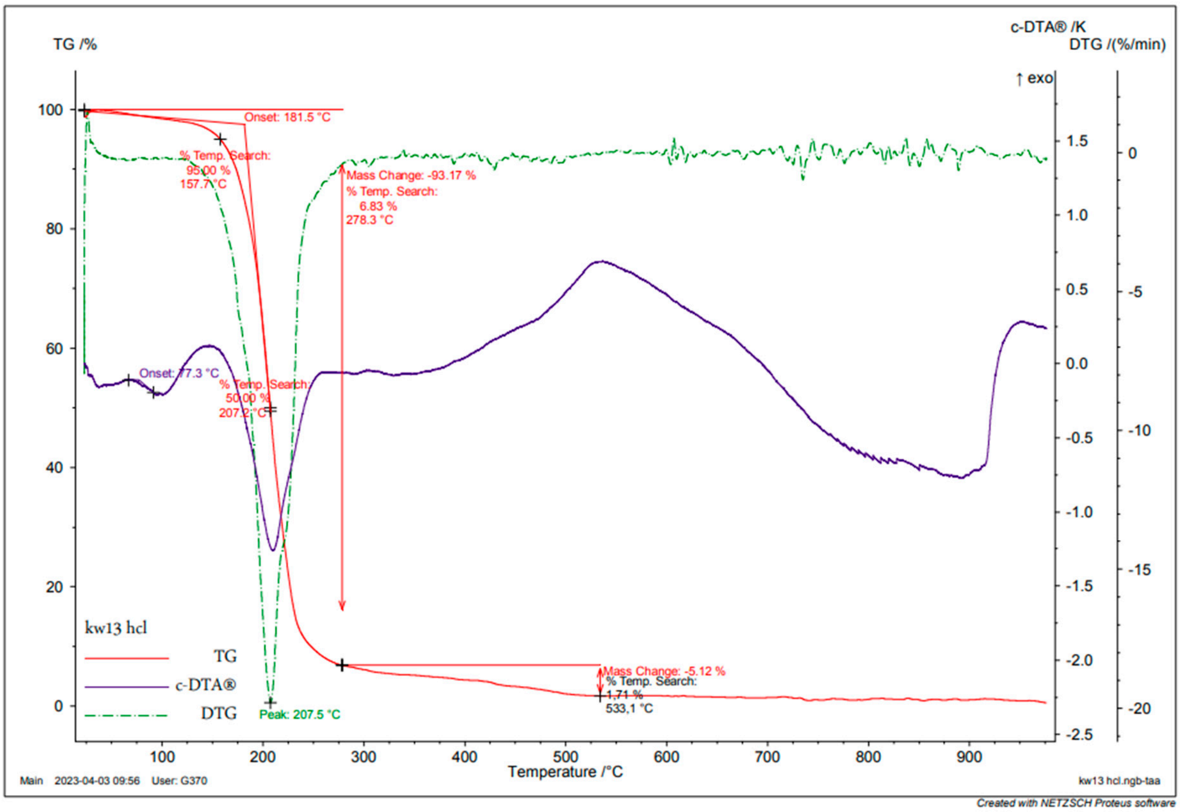

Figure S49. TG, DTG, c-DTA curves of [Glu(OBu)2][HCl].

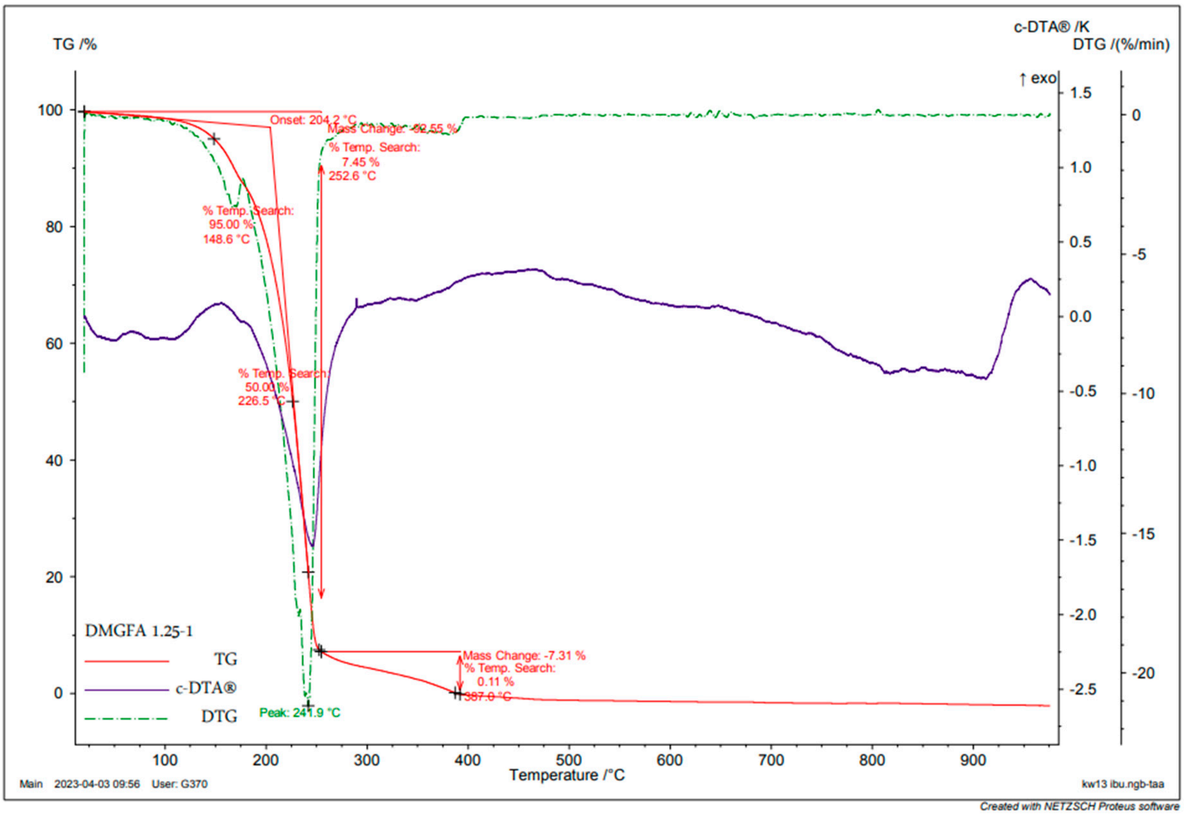

Figure S50. TG, DTG, c-DTA curves of [Glu(Osec-Bu)2][HCl].

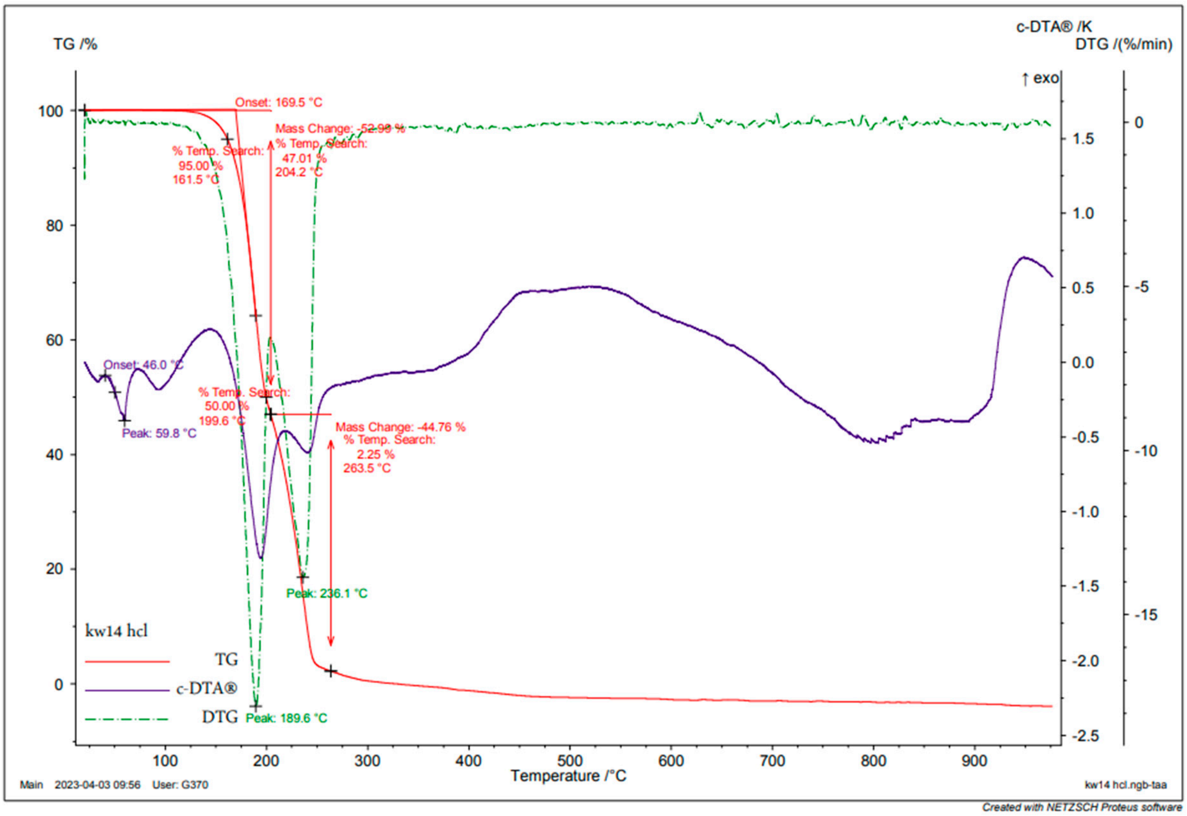

Figure S51. TG, DTG, c-DTA curves of [Glu(OPent)$_2$][HCl].

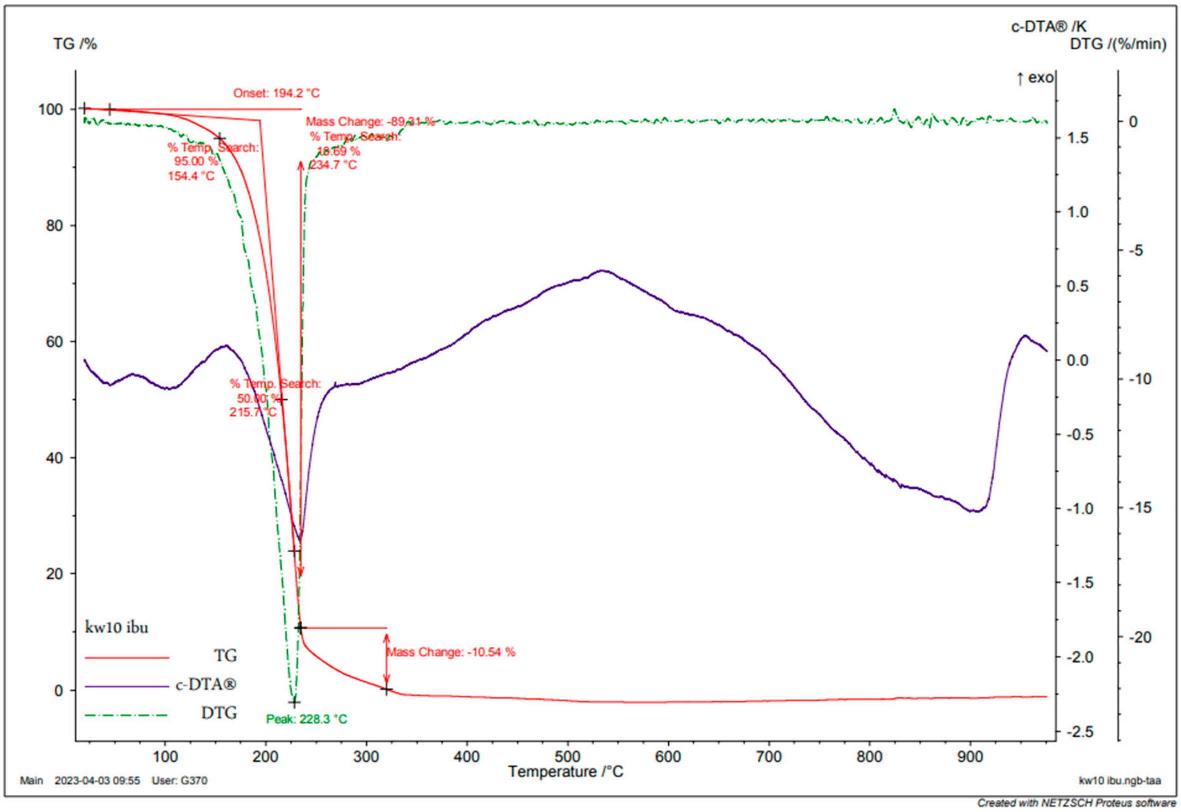

Figure S52. TG, DTG, c-DTA curves of [Glu(OEt)][IBU].

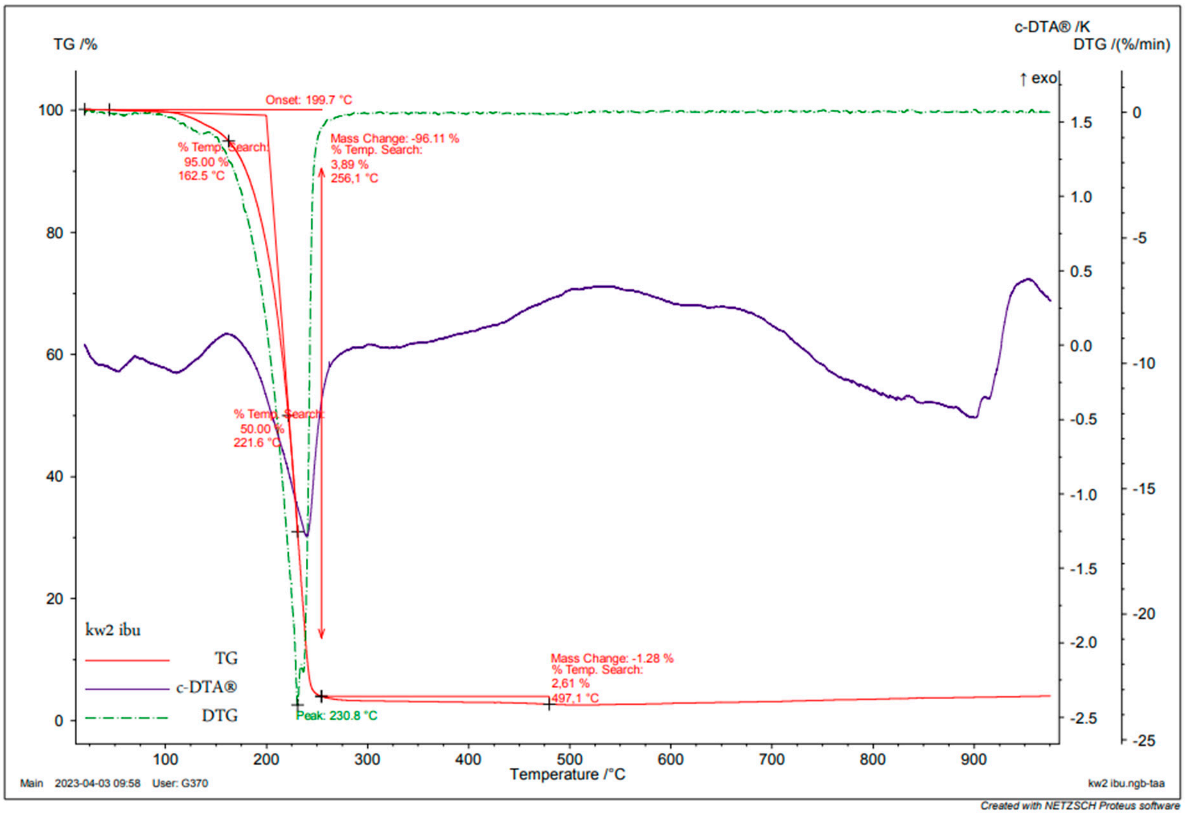

Figure S53. TG, DTG, c-DTA curves of [Glu(OEt)][IBU].

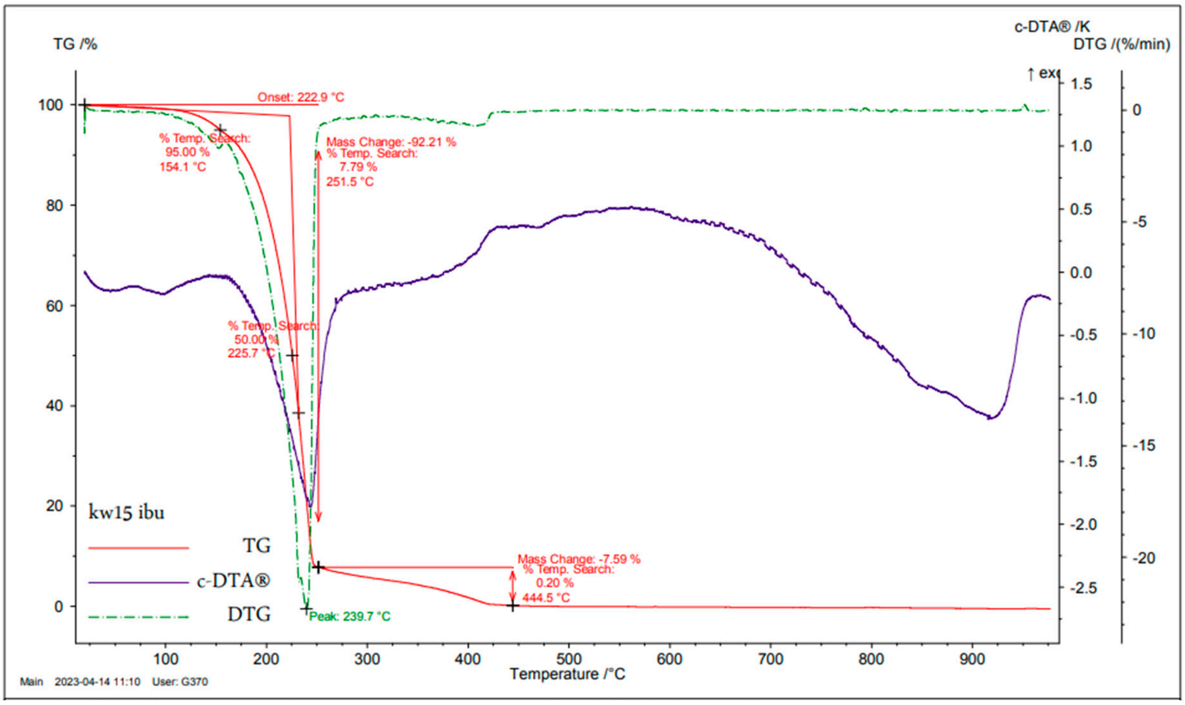

Figure S54. TG, DTG, c-DTA curves of [Glu(OiPr)][IBU].

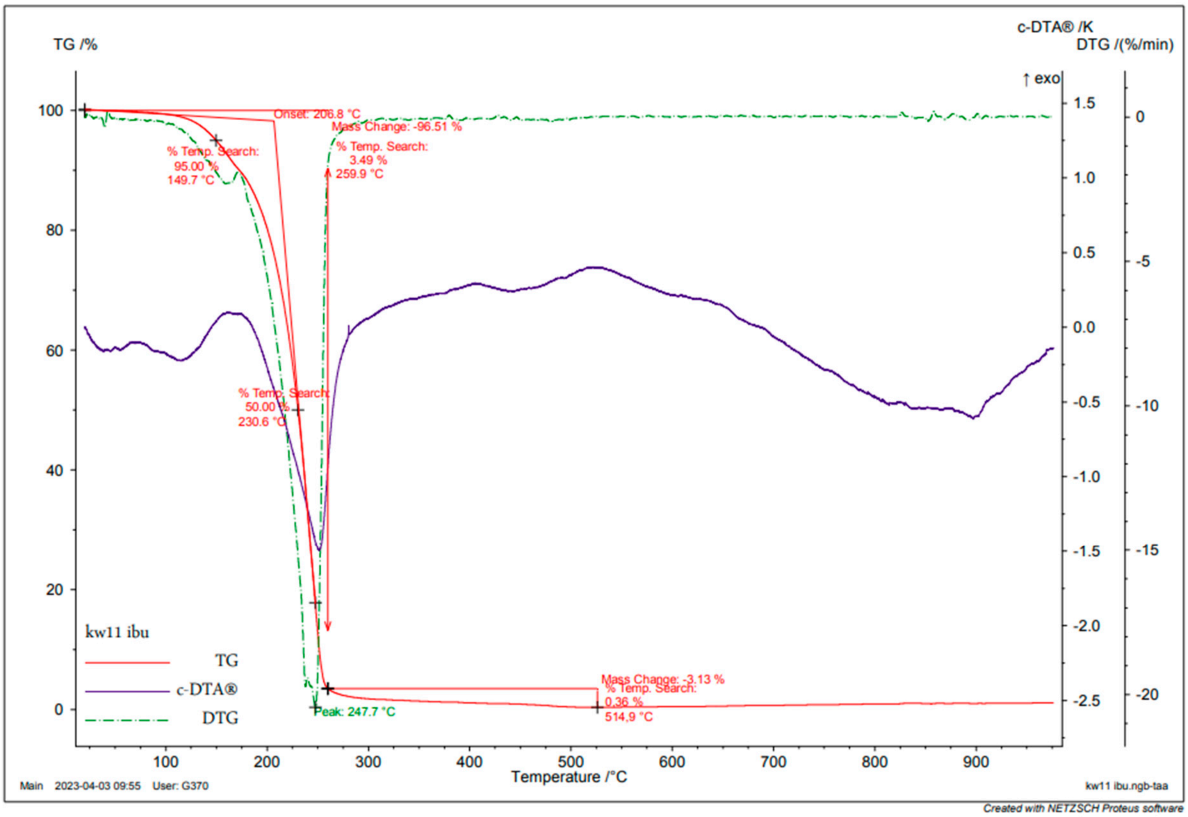

Figure S55. TG, DTG, c-DTA curves of [Glu(OBu)][IBU].

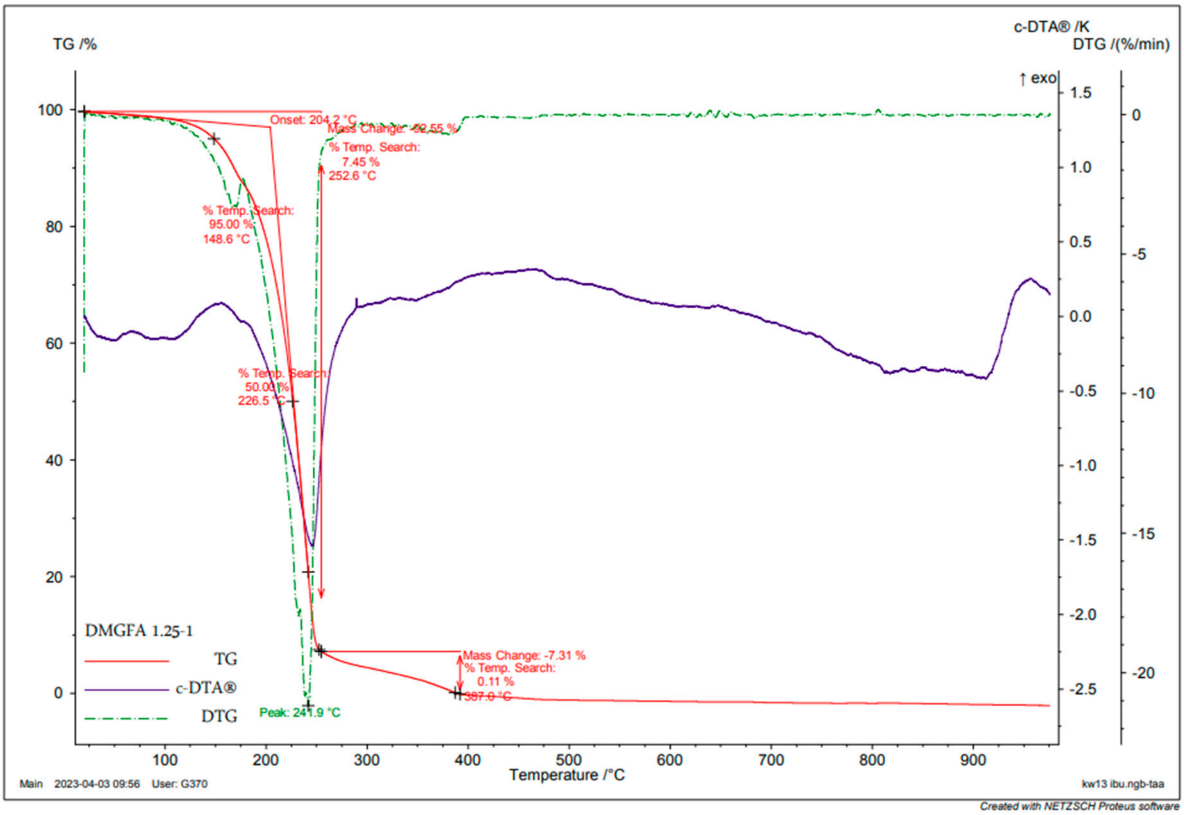

Figure S56. TG, DTG, c-DTA curves of [Glu(Osec-Bu)][IBU].

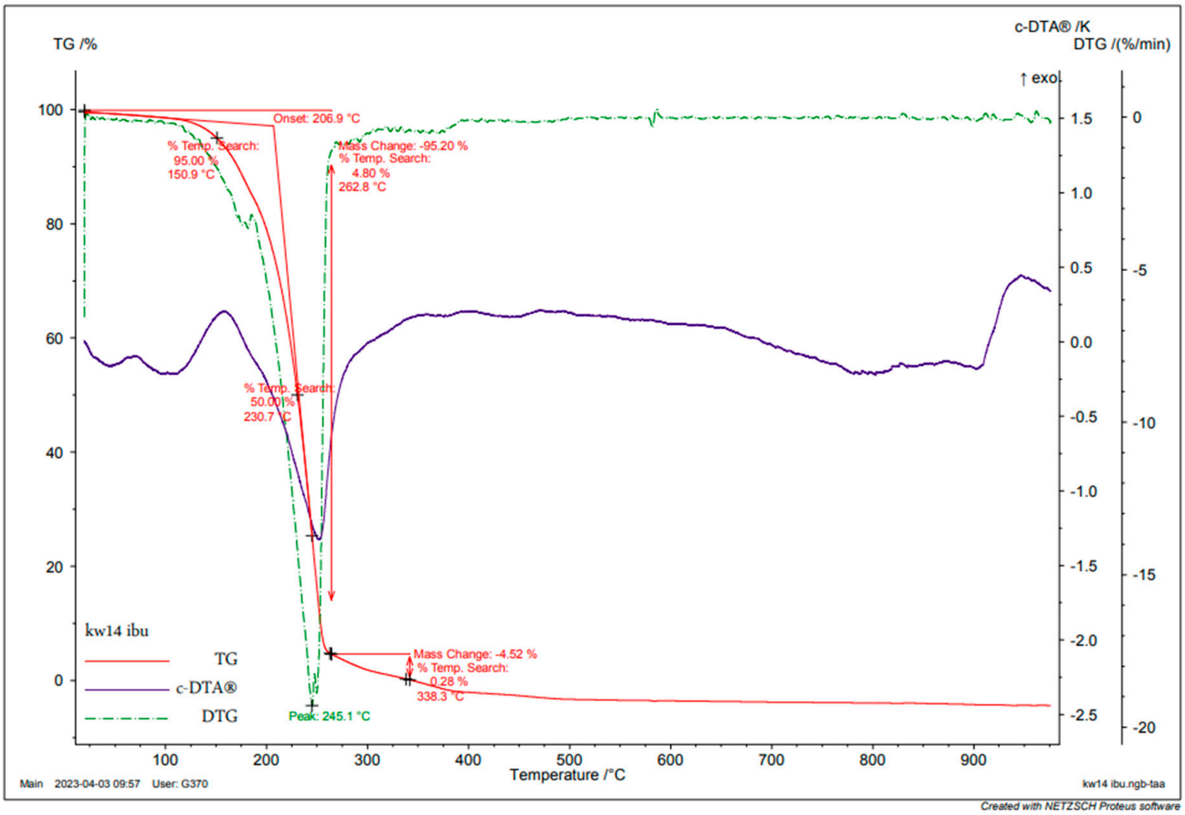

Figure S57. TG, DTG, c-DTA curves of [Glu(OPent)][IBU].

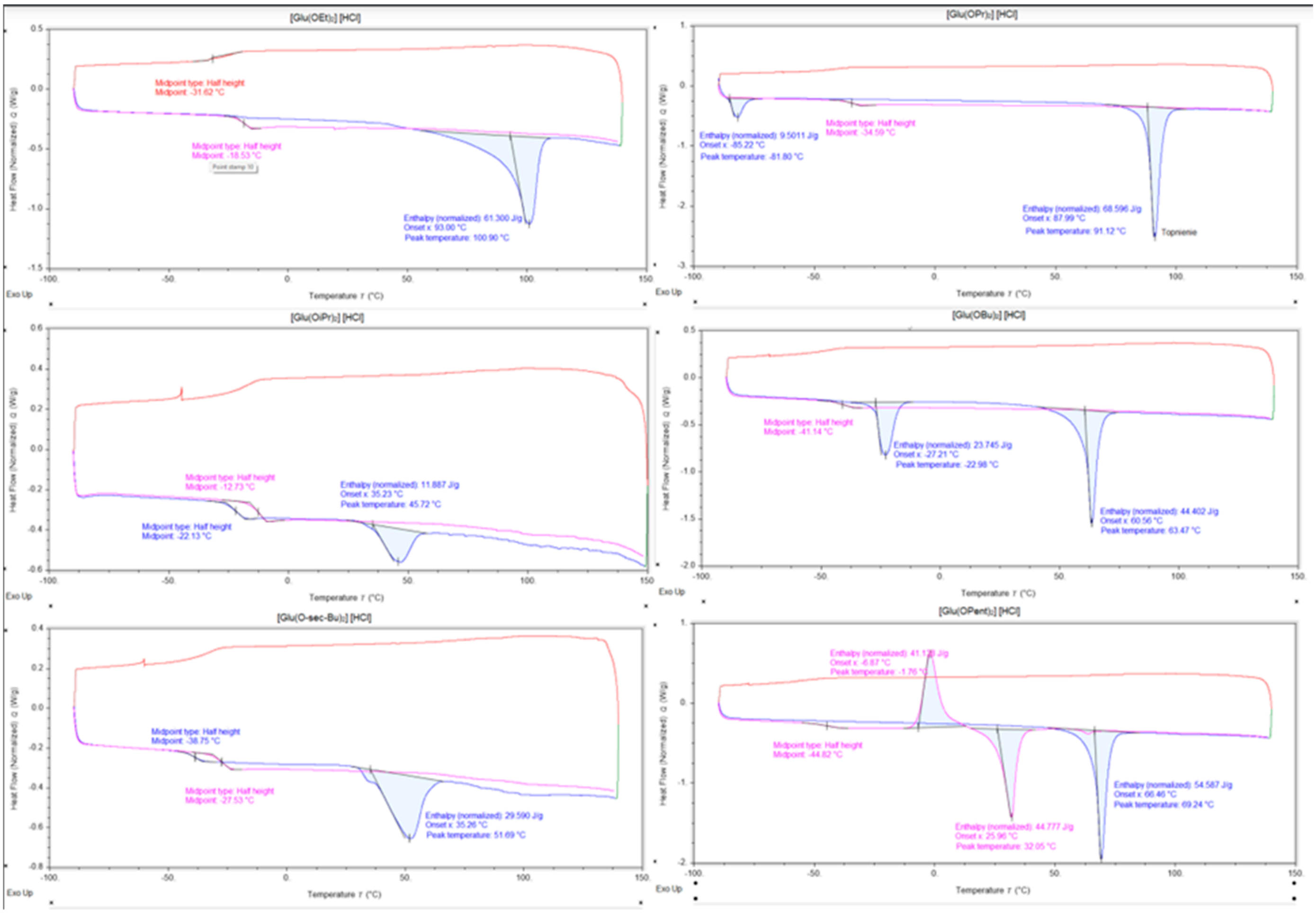

Figure S58. Summary of DSC thermograms for L-glutamic acid alkyl ester hydrochlorides [Glu(OR)$_2$][HCl].

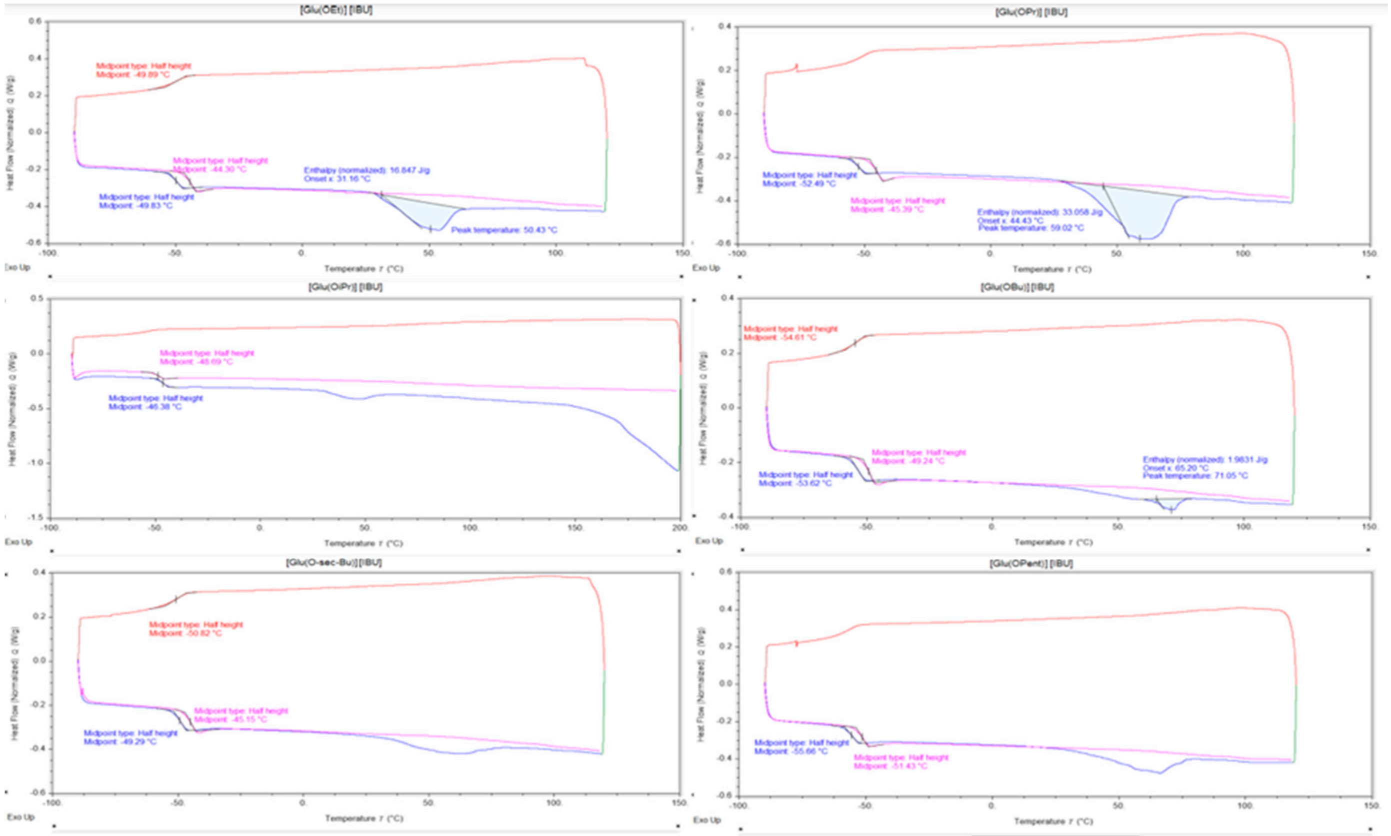

Figure S59. Summary of DSC thermograms for L-glutamic acid alkyl ester ibuprofenates [Glu(OR)][IBU].
